# Supplementary material for: Travel-related control measures to contain the COVID-19 pandemic: an evidence map
Source: BMJ Open. 2021 Apr 9;11(4):e041619. doi: 10.1136/bmjopen-2020-041619 (PMC8042592; doi:10.1136/bmjopen-2020-041619)
Supplement: Supplementary data [file bmjopen-2020-041619supp001.pdf]

## Supplementary file

## S1: Preferred Reporting Items for Systematic reviews and Meta-Analyses extension for Scoping Reviews (PRISMA-ScR) Checklist

| SECTION | ITEM | PRISMA-ScR CHECKLIST ITEM | REPORTED ON PAGE # 1 |
|---|---|---|---|
| **TITLE** | | | |
| Title | 1 | Identify the report as a scoping review. | 2 |
| **ABSTRACT** | | | |
| Structured summary | 2 | Provide a structured summary that includes (as applicable): background, objectives, eligibility criteria, sources of evidence, charting methods, results, and conclusions that relate to the review questions and objectives. | |
| **INTRODUCTION** | | | 4 |
| Rationale | 3 | Describe the rationale for the review in the context of what is already known. Explain why the review questions/objectives lend themselves to a scoping review approach. | 4 |
| Objectives | 4 | Provide an explicit statement of the questions and objectives being addressed with reference to their key elements (e.g., population or participants, concepts, and context) or other relevant key elements used to conceptualize the review questions and/or objectives. | |
| **METHODS** | | | |
| Protocol and registration | 5 | Indicate whether a review protocol exists; state if and where it can be accessed (e.g., a Web address); and if available, provide registration information, including the registration number. | N/A |
| Eligibility criteria | 6 | Specify characteristics of the sources of evidence used as eligibility criteria (e.g., years considered, language, and publication status), and provide a rationale. | 5-6 |
| Information sources* | 7 | Describe all information sources in the search (e.g., databases with dates of coverage and contact with authors to identify additional sources), as well as the date the most recent search was executed. | 5 |
| Search | 8 | Present the full electronic search strategy for at least 1 database, including any limits used, such that it could be repeated. | S2 |
| Selection of sources of evidence† | 9 | State the process for selecting sources of evidence (i.e., screening and eligibility) included in the scoping review. | 7 |
| Data charting process‡ | 10 | Describe the methods of charting data from the included sources of evidence (e.g., calibrated forms or forms that have been tested by the team before their use, and whether data charting was done independently or in duplicate) and any processes for obtaining and confirming data from investigators. | 7-8 |
| Data items | 11 | List and define all variables for which data were sought and any assumptions and simplifications made. | |
| Critical appraisal of individual sources of evidence§ | 12 | If done, provide a rationale for conducting a critical appraisal of included sources of evidence; describe the methods used and how this information was used in any data synthesis (if appropriate). | N/A<br>N/A |
| Synthesis of results | 13 | Describe the methods of handling and summarizing the data that were charted. | |

7-8

St. Michael's
Inspired Care.
Inspiring Science.

| SECTION | ITEM | PRISMA-ScR CHECKLIST ITEM | REPORTED ON PAGE # Figure 1 |
|---|---|---|---|
| **RESULTS** | | | |
| Selection of sources of evidence | 14 | Give numbers of sources of evidence screened, assessed for eligibility, and included in the review, with reasons for exclusions at each stage, ideally using a flow diagram. | Table 1 |
| Characteristics of sources of evidence | 15 | For each source of evidence, present characteristics for which data were charted and provide the citations. | N/A |
| Critical appraisal within sources of evidence | 16 | If done, present data on critical appraisal of included sources of evidence (see item 12). | 8, 23-25 |
| Results of individual sources of evidence | 17 | For each included source of evidence, present the relevant data that were charted that relate to the review questions and objectives. | 8, 23-25 |
| Synthesis of results | 18 | Summarize and/or present the charting results as they relate to the review questions and objectives. | |
| **DISCUSSION** | | | 25-26 |
| Summary of evidence | 19 | Summarize the main results (including an overview of concepts, themes, and types of evidence available), link to the review questions and objectives, and consider the relevance to key groups. | 26-27 |
| Limitations | 20 | Discuss the limitations of the scoping review process. | |
| Conclusions | 21 | Provide a general interpretation of the results with respect to the review questions and objectives, as well as potential implications and/or next steps. | 27 |
| **FUNDING** | | | |
| Funding | 22 | Describe sources of funding for the included sources of evidence, as well as sources of funding for the scoping review. Describe the role of the funders of the scoping review. | 28 |

JBI = Joanna Briggs Institute; PRISMA-ScR = Preferred Reporting Items for Systematic reviews and Meta-Analyses extension for Scoping Reviews.
* Where *sources of evidence* (see second footnote) are compiled from, such as bibliographic databases, social media platforms, and Web sites.
† A more inclusive/heterogeneous term used to account for the different types of evidence or data sources (e.g., quantitative and/or qualitative research, expert opinion, and policy documents) that may be eligible in a scoping review as opposed to only studies. This is not to be confused with *information sources* (see first footnote).
‡ The frameworks by Arksey and O'Malley (6) and Levac and colleagues (7) and the JBI guidance (4, 5) refer to the process of data extraction in a scoping review as data charting.
§ The process of systematically examining research evidence to assess its validity, results, and relevance before using it to inform a decision. This term is used for items 12 and 19 instead of "risk of bias" (which is more applicable to systematic reviews of interventions) to include and acknowledge the various sources of evidence that may be used in a scoping review (e.g., quantitative and/or qualitative research, expert opinion, and policy document).

*From:* Tricco AC, Lillie E, Zarin W, O'Brien KK, Colquhoun H, Levac D, et al. PRISMA Extension for Scoping Reviews (PRISMAScR): Checklist and Explanation. Ann Intern Med. 2018;169:467–473. doi: 10.7326/M18-0850.

St. Michael's
Inspired Care.
Inspiring Science.

**S2:** MEDLINE search strategy and results

| Ovid MEDLINE(R) ALL 1946 to May 01, 2020 | | |
|---|---|---|
| **#** | **Searches** | **Results** |
| 1 | exp Coronavirus/ | 12994 |
| 2 | exp Coronavirus Infections/ | 11480 |
| 3 | (coronavir* or coronovir*).ti,ab,kf. | 14864 |
| 4 | ((corona* or corono*) adj1 (virus* or viral* or virinae*)).ti,ab,kf. | 578 |
| 5 | (ncov or n-cov or 2019nCoV or nCoV2019 or CO?VID-19 or CO?VID19 or WN-CoV or WNCoV or HCoV-19 or HCoV19 or 2019 novel* or SARS-CoV-2 or SARSCoV-2 or SARSCoV2 or SARS-CoV2 or SARSCov19 or SARS-Cov19 or SARSCov-19 or SARS-Cov-19 or Ncovor or Ncorona* or Ncorono* or NcovWuhan* or NcovHubei* or NcovChina* or NcovChinese* or SARS2 or SARS-2 or SARScoron?virus2 or SARS-coron?virus-2 or SARScoron?virus 2 or SARS coron?virus2).ti,ab,kf. | 7647 |
| 6 | (Middle East Respiratory Syndrome or MERS or Severe Acute Respiratory Syndrome or SARS).ti,ab,kf. | 15761 |
| 7 | Influenza, Human/ | 48540 |
| 8 | exp influenzavirus a/ or exp influenzavirus b/ or exp influenzavirus c/ | 44601 |
| 9 | (influenza* not (h?em?phil* influenza* or "h influenza*")).ti,ab,kf. | 95326 |
| 10 | (flu or H1N1 or H2N2 or H3N2 or H1N12 or H5N1).ti,ab,kf. | 36447 |
| 11 | or/1-10 | 140838 |
| 12 | travel/ or air travel/ | 25098 |
| 13 | quarantine/ | 2164 |
| 14 | (travel* or border?).ti. | 25986 |
| 15 | (travel* adj3 (restrict* or reduc* or control* or limit* or lockdown? or ban*)).ab,kf. | 1461 |
| 16 | (travel adj4 (measure? or intervention? or NPI?)).ab,kf. | 401 |
| 17 | (border? adj3 (clos* or restrict* or control* or measure?)).ab,kf. | 1014 |
| 18 | ((screen* or surveil*) adj4 (traveller? or entry or exit or border? or airport?)).ti,ab,kf. | 1124 |
| 19 | visa?.ti,ab,kf. | 2044 |
| 20 | quarantin*.ti,ab,kf. | 4807 |
| 21 | (isolat* adj6 (exposed or suspected or travel* or airport? or border?)).ti,ab,kf. | 7659 |
| 22 | or/12-21 | 59213 |
| 23 | 11 and 22 | 2405 |
| 24 | limit 23 to "humans only (removes records about animals)" | 2247 |



Movsisyan A, *et al. BMJ Open* 2021; 11:e041619. doi: 10.1136/bmjopen-2020-041619

| | | |
|---|---|---|
| 25 | 24 and (armenian or english or french or german or italian or russian).lg. | 2160 |

**S3:** List of (systematic) reviews used for forward and backward citation tracking

1. Ahmad A, Krumkamp R, Reintjes R. Controlling SARS: a review on China's response compared with other SARS-affected countries. Tropical Medicine & International Health. 2009;14(s1):36-45.

2. Aledort JE, Lurie N, Wasserman J, Bozzette SA. Non-pharmaceutical public health interventions for pandemic influenza: an evaluation of the evidence base. BMC Public Health. 2007;7(1):208-.

3. Bitar D, Goubar A, Desenclos JC. International travels and fever screening during epidemics: a literature review on the effectiveness and potential use of non-contact infrared thermometers. Eurosurveillance. 2009;14(6):19115.

4. Bradt DA, Drummond CM. Avian influenza pandemic threat and health systems response. Emergency Medicine Australasia. 2006;18(5-6):430-43.

5. Cowling BJ, Lau LLH, Wu P, Wong HWC, Fang VJ, Riley S, et al. Entry screening to delay local transmission of 2009 pandemic influenza A (H1N1). BMC Infectious Diseases. 2010;10(1):82-.

6. Errett NA, Sauer LM, Rutkow L. An integrative review of the limited evidence on nternational travel bans as an emerging infectious disease disaster control measure. Journal of emergency management (Weston, Mass). 2020;18(1):7-14.

7. Gössling S, Scott D, Hall CM. Pandemics, tourism and global change: a rapid assessment of COVID-19. Journal of Sustainable Tourism. 2020:1-20.

8. Group WHOW, Bell D, Nicoll A, Fukuda K, Horby P, Monto A, et al. Non-pharmaceutical interventions for pandemic influenza, national and community measures. Emerging infectious diseases. 2006;12(1):88-94.

9. Huizer YL, Swaan CM, Leitmeyer KC, Timen A. Usefulness and applicability of infectious disease control measures in air travel: A review. Travel Medicine and Infectious Disease. 2015;13(1):19-30.

10. Inglesby TV, Nuzzo JB, O'Toole T, Henderson DA. Disease Mitigation Measures in the Control of Pandemic Influenza. Biosecurity and Bioterrorism: Biodefense Strategy, Practice, and Science. 2006;4(4):366-75.

11. Jefferson T, Del Mar CB, Dooley L, Ferroni E, Al-Ansary LA, Bawazeer GA, et al. Physical interventions to interrupt or reduce the spread of respiratory viruses. Cochrane Database of Systematic Reviews. 2011(7).

12. Juneau C-E, Pueyo T, Bell M, Gee G, Potvin L. Evidence-based, cost-effective interventions to suppress the COVID-19 pandemic: a rapid systematic review. medRxiv. 2020:2020.04.20.20054726-2020.04.20.

13. Kelly HA, Priest PC, Mercer GN, Dowse GK. We should not be complacent about our population-based public health response to the first influenza pandemic of the 21st century. BMC public health. 2011;11:78-.

14. Lee VJ, Lye DC, Wilder-Smith A. Combination strategies for pandemic influenza response - a systematic review of mathematical modeling studies. BMC Medicine. 2009;7(1):76-.

15. Lugnér AK, Postma MJ. Mitigation of pandemic influenza: review of cost–effectiveness studies. Expert Review of Pharmacoeconomics & Outcomes Research. 2009;9(6):547-58.

16. Mateus ALP, Otete HE, Beck CR, Dolan GP, Nguyen-Van-Tam JS. Effectiveness of travel restrictions in the rapid containment of human influenza: a systematic review. Bulletin of the World Health Organization. 2014;92(12):868-80D.

17. Mouchtouri VA, Christoforidou EP, An der Heiden M, Menel Lemos C, Fanos M, Rexroth U, et al. Exit and Entry Screening Practices for Infectious Diseases among Travelers at Points of Entry: Looking for Evidence on Public Health Impact. International journal of environmental research and public health. 2019;16(23):4638-.

18. Nicola M, Alsafi Z, Sohrabi C, Kerwan A, Al-Jabir A, Iosifidis C, et al. The Socio-Economic Implications of the Coronavirus and COVID-19 Pandemic: A Review. International journal of surgery (London, England). 2020:S1743-9191(20)30316-2.

19. Nussbaumer-Streit B, Mayr V, Dobrescu AI, Chapman A, Persad E, Klerings I, et al. Quarantine alone or in combination with other public health measures to control COVID-19: a rapid review. Cochrane Database of Systematic Reviews. 2020(4).

20. Paudel S, Dangal G, Chalise A, Bhandari TR, Dangal O. The Coronavirus Pandemic: What Does the Evidence Show? Journal of Nepal Health Research Council. 2020;18(1):1-9.

21. Rashid H, Ridda I, King C, Begun M, Tekin H, Wood JG, et al. Evidence compendium and advice on social distancing and other related measures for response to an influenza pandemic. Paediatric Respiratory Reviews. 2015;16(2):119-26.

22. Röhr S, Müller F, Jung F, Apfelbacher C, Seidler A, Riedel-Heller SG. [Psychosocial Impact of Quarantine Measures During Serious Coronavirus Outbreaks: A Rapid Review]. Psychiatrische Praxis. 2020;47(4):179-89.

23. Ryu S, Gao H, Wong JY, Shiu EYC, Xiao J, Fong MW, et al. Nonpharmaceutical Measures for Pandemic Influenza in Nonhealthcare Settings-International Travel- Related Measures. Emerging infectious diseases. 2020;26(5):961-6.

24. Selvey LA, Antão C, Hall R. Entry screening for infectious diseases in humans. Emerging infectious diseases. 2015;21(2):197-201.

25. Taghrir MH, Akbarialiabad H, Ahmadi Marzaleh M. Efficacy of Mass Quarantine as Leverage of Health System Governance During COVID-19 Outbreak: A Mini Policy Review. Archives of Iranian medicine. 2020;23(4):265-7.

26. Vaidya R, Herten-Crabb A, Spencer J, Moon S, Lillywhite L. Travel restrictions and infectious disease outbreaks. Journal of Travel Medicine. 2020.

27. Walters CE, Meslé MMI, Hall IM. Modeling the global spread of diseases: A review of current practice and capability. Epidemics. 2018;25:1-8.

28. Wilder-Smith A. The severe acute respiratory syndrome: impact on travel and tourism. Travel medicine and infectious disease. 2006;4(2):53-60.

29. Wilder-Smith A, Paton NI, Goh KT. Experience of severe acute respiratory syndrome in singapore: importation of cases, and defense strategies at the airport. Journal of travel medicine. 2003;10(5):259-62.

30. WHO. Handbook for public health capacity-building at ground crossings and cross-border collaboration. World Health Organization; 2020.

**S4:** Data extraction form

Study information:

- Study ID
- Study title
- Publication year
- Study source (journal, report, pre-print publication)
- For pre-print publication only: date of publication

Study design:

- Study type (e.g. modeling study, cross-sectional study, econometric study)
- Verbal summary of study (e.g., stochastic discrete event simulation model)
- Comments

Population/context:

- Country in which measure is implemented
- Study setting (e.g., Auckland international airport)
- Region protected by travel-related measure
- Region restricted by travel-related measure
- Short description of population studied (e.g., international travelers including flight passenger and crew members arriving between 27 April and 22 June 2009)
- Interconnectedness of the region
- Comments

Characteristics of pathogen/disease:

- Pathogen/disease (e.g., COVID-19, influenza)
- For influenza only: subtype (e.g. H1N1), year, endemic vs. pandemic
- Real vs. hypothetical disease outbreak
- Comments

Intervention:

- Broad measure category (e.g., entry screening)
- Verbal summary of specific travel-related measure(s) (e.g., in-flight questionnaire/symptoms)
- Real vs. hypothetical intervention
- Implementation of travel-related measure
- Phase of disease/epidemic spread (e.g. early-stage vs. late-stage)
- Co-interventions (assessed in the study)
- Co-interventions (not assessed in the study)
- Comments

Primary outcomes (repeated for each primary outcome):

- Outcome category (i.e. infectious-disease related health outcome, other health outcome, economic outcome, social outcome)
- Description of outcome
- Outcome attributable to travel-related measures (yes/no)
- Length of follow-up

- Comments

Secondary outcomes

- Description of any secondary (non-quantitative) outcomes



Movsisyan A, et al. BMJ Open 2021; 11:e041619. doi: 10.1136/bmjopen-2020-041619

**S5:** Studies included in the evidence map


1. Adekunle AI, Meehan M, Rojaz Alvarez D, Trauer J, McBryde E. Delaying the COVID-19 epidemic in Australia: Evaluating the effectiveness of international travel bans. medRxiv. 2020:2020.03.22.20041244.

2. Aleta A, Hu Q, Ye J, Ji P, Moreno Y. A data-driven assessment of early travel restrictions related to the spreading of the novel COVID-19 within mainland China. medRxiv. 2020:2020.03.05.20031740.

3. Anonymous. Thermal image scanners to detect fever in airline passengers, Vancouver and Toronto, 2003. Canada Communicable Disease Report.30(19):165-7.

4. Anzai A, Kobayashi T, Linton NM, Kinoshita R, Hayashi K, Suzuki A, et al. Assessing the Impact of Reduced Travel on Exportation Dynamics of Novel Coronavirus Infection (COVID-19). Journal of Clinical Medicine. 2020;9(2):24.

5. Arima Y, Shimada T, Suzuki M, Suzuki T, Kobayashi Y, Tsuchihashi Y, et al. Severe Acute Respiratory Syndrome Coronavirus 2 Infection among Returnees to Japan from Wuhan, China, 2020. Emerging Infectious Diseases. 2020;26(7):10.

6. Arino J, Jordan R, van den Driessche P. Quarantine in a multi-species epidemic model with spatial dynamics. Mathematical Biosciences. 2007;206(1):46-60.

7. Bajardi P, Poletto C, Ramasco JJ, Tizzoni M, Colizza V, Vespignani A. Human mobility networks, travel restrictions, and the global spread of 2009 H1N1 pandemic. PLoS ONE. 2011;6(1).

8. Banholzer N, van Weenen E, Kratzwald B, Seeliger A, Tschernutter D, Bottrighi P, et al. Estimating the impact of non-pharmaceutical interventions on documented infections with COVID-19: A cross-country analysis. medRxiv. 2020:2020.04.16.20062141.

9. Bolton KJ, McCaw JM, Moss R, Morris RS, Wang S, Burma A, et al. Likely effectiveness of pharmaceutical and non-pharmaceutical interventions for mitigating influenza virus transmission in Mongolia. Bull WHO. 2012;90(4):264-71.

10. Boyd M, Baker MG, Mansoor OD, Kvizhinadze G, Wilson N. Protecting an island nation from extreme pandemic threats: Proof-of-concept around border closure as an intervention. PLoS ONE [Electronic Resource]. 2017;12(6):e0178732.

11. Boyd M, Mansoor OD, Baker MG, Wilson N. Economic evaluation of border closure for a generic severe pandemic threat using New Zealand Treasury methods. Australian & New Zealand Journal of Public Health. 2018;42(5):444-6.

12. Caley P, Becker NG, Philip DJ. The waiting time for inter-country spread of pandemic influenza. PLoS ONE. 2007;2(1).

13. Chang M-C, Kahn R, Li Y-A, Lee C-S, Buckee CO, Chang H-H. Modeling the impact of human mobility and travel restrictions on the potential spread of SARS-CoV-2 in Taiwan. medRxiv. 2020:2020.04.07.20053439.

14. Cheng HY, Li SY, Yang CH. Initial rapid and proactive response for the COVID-19 outbreak - Taiwan's experience. Journal of the Formosan Medical Association. 2020;119(4):771-3.

15. Chinazzi M, Davis JT, Ajelli M, Gioannini C, Litvinova M, Merler S, et al. The effect of travel restrictions on the spread of the 2019 novel coronavirus (COVID-19) outbreak. Science. 2020;368(6489):395-400.

16. Chiyomaru K, Takemoto K. Global COVID-19 transmission rate is influenced by precipitation seasonality and the speed of climate temperature warming. medRxiv. 2020:2020.04.10.20060459.

17. Chong KC, Ying Zee BC. Modeling the impact of air, sea, and land travel restrictions supplemented by other interventions on the emergence of a new influenza pandemic virus. BMC Infect Dis. 2012;12.

18. Chung LH. Impact of pandemic control over airport economics: Reconciling public health with airport business through a streamlined approach in pandemic control. J Air Transp Manage. 2015;44-45:42-53.

19. Ciofi degli Atti ML, Merler S, Rizzo C, Ajelli M, Massari M, Manfredi P, et al. Mitigation measures for pandemic influenza in Italy: An individual based model considering different scenarios. PLoS ONE. 2008;3(3).

20. Clifford SJ, Pearson CAB, Klepac P, Z V, voort K, Quilty BJ, et al. Interventions targeting air travellers early in the pandemic may delay local outbreaks of SARS-CoV-2. medRxiv. 2020:2020.02.12.20022426.

21. Colizza V, Barrat A, Barthelemy M, Valleron AJ, Vespignani A. Modeling the worldwide spread of pandemic influenza: Baseline case and containment interventions. PLoS Med. 2007;4(1):0095-110.

22. Cooper BS, Pitman RJ, Edmunds WJ, Gay NJ. Delaying the international spread of pandemic influenza. PLoS Med. 2006;3(6):0845-55.

23. Costantino V, Heslop DJ, MacIntyre C, Raina i. The effectiveness of full and partial travel bans against COVID-19 spread in Australia for travellers from China. medRxiv. 2020:2020.03.09.20032045.

24. Cowling BJ, Ali ST, Ng TWY, Tsang TK, Li JCM, Fong MW, et al. Impact assessment of non-pharmaceutical interventions against coronavirus disease 2019 and influenza in Hong Kong: an observational study. The lancet Public Health. 2020;17:17.

25. Dandekar R, Barbastathis G. Quantifying the effect of quarantine control in Covid-19 infectious spread using machine learning. medRxiv. 2020:2020.04.03.20052084.

26. De Vlas SJ, Feng D, Cooper BS, Fang LQ, Cao WC, Richardus JH. The impact of public health control measures during the SARS epidemic in mainland China. Trop Med Int Health. 2009;14(SUPPL. 1):101-4.

27. Ediriweera DS, de Silva NR, Malavige NG, de Silva HJ. An epidemiological model to aid decision-making for COVID-19 control on Sri Lanka. medRxiv. 2020:2020.04.11.20061481.

28. Eichner M, Schwehm M, Wilson N, Baker MG. Small islands and pandemic influenza: Potential benefits and limitations of travel volume reduction as a border control measure. BMC Infect Dis. 2009;9:160.

29. Epstein JM, Goedecke DM, Yu F, Morris RJ, Wagener DK, Bobashev GV. Controlling pandemic flu: The value of international air travel restrictions. PLoS ONE. 2007;2(5).

30. Fang H, Wang L, Yang Y. Human Mobility Restrictions and the Spread of the Novel Coronavirus (2019-nCoV) in China. medRxiv. 2020:2020.03.24.20042424.

31. Ferguson NM, Cummings DAT, Fraser C, Cajka JC, Cooley PC, Burke DS. Strategies for mitigating an influenza pandemic. Nature. 2006;442(7101):448-52.

32. Flahault A, Vergu E, Coudeville L, Grais RF. Strategies for containing a global influenza pandemic. Vaccine. 2006;24(44-46):6751-5.

33. Fujita M, Sato H, Kaku K, Tokuno S, Kanatani Y, Suzuki S, et al. Airport quarantine inspection, follow-up observation, and the prevention of pandemic influenza. Aviat Space Environ Med. 2011;82(8):782-9.

34. Germann TC, Kadau K, Longini Jr IM, Macken CA. Mitigation strategies for pandemic influenza in the United States. Proc Natl Acad Sci U S A. 2006;103(15):5935-40.

35. Glass K, Becker NG. Evaluation of measures to reduce international spread of SARS. Epidemiol Infect. 2006;134(5):1092-101.

36. Gostic KM, Gomez ACR, Mummah RO, Kucharski AJ, Lloyd-Smith JO. Estimated effectiveness of symptom and risk screening to prevent the spread of COVID-19. eLife. 2020;9.

37. Gostic KM, Kucharski AJ, Lloyd-Smith JO. Effectiveness of traveller screening for emerging pathogens is shaped by epidemiology and natural history of infection. eLife. 2015;2015(4).

38. Goubar A, Bitar D, Cao WC, Feng D, Fang LQ, Desenclos JC. An approach to estimate the number of SARS cases imported by international air travel. Epidemiol Infect. 2009;137(7):1019-31.

39. Gunaratnam PJ, Tobin S, Seale H, Marich A, McAnulty J. Airport arrivals screening during pandemic (H1N1) 2009 influenza in New South Wales, Australia. Med J Aust. 2014;200(5):290-2.

40. Hale MJ, Hoskins RS, Baker MG. Screening for influenza A(H1N1)pdm09, Auckland International Airport, New Zealand. Emerging Infectious Diseases. 2012;18(5):866-8.

41. Hamidouche M. COVID-19 Epidemic in Algeria: Assessment of the implemented preventive strategy. medRxiv. 2020:2020.04.21.20074443.

42. He J, Chen G, Jiang Y, Jin R, He M, Shortridge A, et al. Comparative Analysis of COVID-19 Transmission Patterns in Three Chinese Regions vs. South Korea,Italy and Iran. medRxiv. 2020:2020.04.09.20053223.

43. Hien TT, Boni MF, Bryant JE, Ngan TT, Wolbers M, Nguyen TD, et al. Early pandemic influenza (2009 H1N1) in Ho Chi Minh City, Vietnam: a clinical virological and epidemiological analysis. PLoS Medicine / Public Library of Science.7(5):e1000277.

44. Hollingsworth TD, Ferguson NM, Anderson RM. Will travel restrictions control the international spread of pandemic influenza? [3]. Nat Med. 2006;12(5):497-9.

45. Hossain MP, Junus A, Zhu X, Jia P, Wen T-H, Pfeiffer D, et al. The effects of border control and quarantine measures on global spread of COVID-19. medRxiv. 2020:2020.03.13.20035261.

46. Hou J, Hong J, Ji B, Dong B, Chen Y, Ward MP, et al. Changing transmission dynamics of COVID-19 in China: a nationwide population-based piecewise mathematical modeling study. medRxiv. 2020:2020.03.27.20045757.

47. Hsieh YH, King CC, Chen CW, Ho MS, Hsu SB, Wu YC. Impact of quarantine on the 2003 SARS outbreak: a retrospective modeling study. Journal of Theoretical Biology. 2006;244(4):729-36.

48. Hsieh YH, Van Den Driessche P, Wang L. Impact of travel between patches for spatial spread of disease. Bulletin of Mathematical Biology. 2007;69(4):1355-75.

49. Jia JS, Lu X, Yuan Y, Xu G, Jia J, Christakis NA. Population flow drives spatio-temporal distribution of COVID-19 in China. Nature. 2020.

50. Jiang X, eng, Chang L, Shi Y. How does the outbreak of 2019-nCoV spread in mainland China? A retrospective analysis of the dynamic transmission routes. medRxiv. 2020:2020.03.01.20029645.

51. Kerneis S, Grais RF, Boelle PY, Flahault A, Vergu E. Does the effectiveness of control measures depend on the influenza pandemic profile? PLoS ONE [Electronic Resource]. 2008;3(1):e1478.

52. Khan K, Eckhardt R, Brownstein JS, Naqvi R, Hu W, Kossowsky D, et al. Entry and exit screening of airline travellers during the A(H1N1) 2009 pandemic: A retrospective evaluation. Bull WHO. 2013;91(5):368-76.

53. Kim S, Chang DE, editors. Border screening vs. community level disease control for infectious diseases: Timing and effectiveness2017: American Institute of Physics Inc.

54. Kong XS, Liu F, Wang HB, Yang RF, Chen DB, Wang XX, et al. Epidemic prevention and control measures in China significantly curbed the epidemic of COVID-19 and influenza. medRxiv. 2020:2020.04.09.20058859.

55. Kraemer MUG, Yang CH, Gutierrez B, Wu CH, Klein B, Pigott DM, et al. The effect of human mobility and control measures on the COVID-19 epidemic in China. Science. 2020;25:25.

56. Kuo JS, Lee YH, Heish JW, Lin MC, Yang SY. Initial evaluation on screening of novel influenza A (H1N1) at international ports in Taiwan. Taiwan Epidemiol Bull. 2009;25(9):254-67.

57. Lai S, Ruktanonchai NW, Zhou L, Prosper O, Luo W, Floyd JR, et al. Effect of non-pharmaceutical interventions for containing the COVID-19 outbreak in China. medRxiv. 2020:2020.03.03.20029843.

58. Lam EHY, Cowling BJ, Cook AR, Wong JYT, Lau MSY, Nishiura H. The feasibility of age-specific travel restrictions during influenza pandemics. Theor Biol Med Model. 2011;8(1).

59. Lau H et al.. Positive impact of lockdown in Wuhan on containing the COVID-19 outbreak in China ;Journal of Travel Medicine; Oxford Academic. Journal of Travel Medicine. 2020.

60. Lee JM, Choi D, Cho G, Kim Y. The effect of public health interventions on the spread of influenza among cities. Journal of Theoretical Biology. 2012;293:131-42.

61. Li D, Liu Z, Liu Q, Gao Z, Zhu J, Yang J, et al. Estimating the Efficacy of Traffic Blockage and Quarantine for the Epidemic Caused by 2019-nCoV (COVID-19). medRxiv. 2020:2020.02.14.20022913.

62. Lin H, Liu W, Gao H, Nie J, Fan Q. Trends in Transmissibility of 2019 Novel Coronavirus-infected Pneumonia in Wuhan and 29 Provinces in China. medRxiv. 2020:2020.02.21.20026468.

63. Linka K, Peirlinck M, Sahli Costabal F, Kuhl E. Outbreak dynamics of COVID-19 in Europe and the effect of travel restrictions. medRxiv. 2020:2020.04.18.20071035.

64. Liu H, Bai X, Shen H, Pang X, Liang Z, Liu Y. Synchronized travel restrictions across cities can be effective in COVID-19 control. medRxiv. 2020a:2020.04.02.20050781.

65. Liu K, Ai S, Song S, Zhu G, Tian F, Li H, et al. Population movement, city closure in Wuhan and geographical expansion of the 2019-nCoV pneumonia infection in China in January 2020. Clinical infectious diseases. 2020.

66. Liu XN, Takeuchi Y. Spread of disease with transport-related infection and entry screening. Journal of Theoretical Biology. 2006;242(2):517-28.

67. Malmberg H, Britton T. Inflow restrictions can prevent epidemics when contact tracing efforts are effective but have limited capacity. medRxiv. 2020:2020.04.01.20050401.

68. Malone JD, Brigantic R, Muller GA, Gadgil A, Delp W, McMahon BH, et al. U.S. airport entry screening in response to pandemic influenza: Modeling and analysis. Travel Med Infect Dis. 2009;7(4):181-91.

69. Mandal S, Bhatnagar T, Arinaminpathy N, Agarwal A, Chowdhury A, Murhekar M, et al. Prudent public health intervention strategies to control the coronavirus disease 2019 transmission in India: A mathematical model-based approach. Indian Journal of Medical Research. 2020;23:23.

70. Marcelino J, Kaiser M. Critical paths in a metapopulation model of H1N1: Efficiently delaying influenza spreading through flight cancellation. PLoS Currents. 2012.

71. Mbuvha R, Marwala T. Bayesian Inference of COVID-19 Spreading Rates in South Africa. medRxiv. 2020:2020.04.28.20083873.

72. Mondal S, Ghosh S. Searching the Sigmoid-type trend in Lock Down period covid19 data of India and its different states. medRxiv. 2020:2020.04.25.20079624.

73. Moriarty L. et al., Prevention, et al. Public Health Responses to COVID-19 Outbreaks on Cruise Ships — Worldwide, February–March 2020. MMWR Morb Mortal Wkly Rep. 2020.

74. Mummert A, Weiss H, Long LP, Amigo JM, Wan XF. A perspective on multiple waves of influenza pandemics. PLoS ONE [Electronic Resource]. 2013;8(4):e60343.

75. Muraduzzaman AKM, Khan MH, Parveen R, Sultana S, Alam AN, Akram A, et al. Event based surveillance of Middle East Respiratory Syndrome Coronavirus (MERS- CoV) in Bangladesh among pilgrims and travelers from the Middle East: An update for the period 2013-2016. PLoS ONE [Electronic Resource]. 2018;13(1):e0189914.

76. Nakata Y, Rost G. Global analysis for spread of infectious diseases via transportation networks. Journal of Mathematical Biology. 2015;70(6):1411-56.

77. Nigmatulina KR, Larson RC. Living with influenza: Impacts of government imposed and voluntarily selected interventions. European Journal of Operational Research. 2009;195(2):613-27.

78. Nishiura H, Wilson N, Baker MG. Quarantine for pandemic influenza control at the borders of small island nations. BMC Infect Dis. 2009;9.

79. Odendaal WG. A Method to Model Outbreaks of New Infectious Diseases with Pandemic Potential such as COVID-19. medRxiv. 2020:2020.03.11.20034512.

80. Pan J, Yao Y, Liu Z, Li M, Wang Y, Dong W, et al. Effectiveness of control strategies for Coronavirus Disease 2019: a SEIR dynamic modeling study. medRxiv. 2020:2020.02.19.20025387.

81. Pan X, Ojcius DM, Gao T, Li Z, Pan C, Pan C. Lessons learned from the 2019-nCoV epidemic on prevention of future infectious diseases. Microbes Infect. 2020a;22(2):86-91.

82. Pang X, Zhu Z, Xu F, Guo J, Gong X, Liu D, et al. Evaluation of Control Measures Implemented in the Severe Acute Respiratory Syndrome Outbreak in Beijing, 2003. J Am Med Assoc. 2003;290(24):3215-21.

83. Pinkas J, Jankowski M, Szumowski L, Lusawa A, Zgliczynski WS, Raciborski F, et al. Public Health Interventions to Mitigate Early Spread of SARS-CoV-2 in Poland. Medical Science Monitor. 2020;26:e924730.

84. Pitman RJ, Cooper BS, Trotter CL, Gay NJ, Edmunds WJ. Entry screening for severe acute respiratory syndrome (SARS) or influenza: Policy evaluation. Br Med J. 2005;331(7527):1242-3.

85. Priest PC, Jennings LC, Duncan AR, Brunton CR, Baker MG. Effectiveness of border screening for detecting influenza in arriving airline travelers. American Journal of Public Health. 2013;103(8):1412-8.

86. Pullano G, Pinotti F, Valdano E, Boelle PY, Poletto C, Colizza V. Novel coronavirus (2019-nCoV) early-stage importation risk to Europe, January 2020. Euro Surveillance: Bulletin Europeen sur les Maladies Transmissibles = European Communicable Disease Bulletin.25(4):01.

87. Qiu Y, Chen X, Shi W. Impacts of social and economic factors on the transmission of coronavirus disease (COVID-19) in China. medRxiv. 2020:2020.03.13.20035238.

88. Quilty BJ, Diamond C, Liu Y, Gibbs H, Russell TW, Jarvis CI, et al. The effect of inter-city travel restrictions on geographical spread of COVID-19: Evidence from Wuhan, China. medRxiv. 2020:2020.04.16.20067504.

89. Ray D, Salvatore M, Bhattacharyya R, Wang L, Mohammed S, Purkayastha S, et al. Predictions, role of interventions and effects of a historic national lockdown in India's response to the COVID-19 pandemic: data science call to arms. medRxiv. 2020:2020.04.15.20067256.

90. Sakaguchi H, Tsunoda M, Wada K, Ohta H, Kawashima M, Yoshino Y, et al. Assessment of border control measures and community containment measures used in japan during the early stages of Pandemic (H1N1) 2009. PLoS ONE. 2012;7(2).

91. Samaan G, Patel M, Spencer J, Roberts L. Border screening for SARS in Australia: What has been learnt? Med J Aust. 2004;180(5):220-4.

92. Sang Z, Qiu ZP, Yan XF, Zou Y. Assessing the effect of non-pharmaceutical interventions on containing an emerging disease.. Mathematical Biosciences and Engineering.9(1):147-64.

93. Scala A, Flori A, Spelta A, ro, Brugnoli E, Cinelli M, et al. Between Geography and Demography: Key Interdependencies and Exit Mechanisms for Covid-19. medRxiv. 2020:2020.04.09.20059592.

94. Scalia Tomba G, Wallinga J. A simple explanation for the low impact of border control as a countermeasure to the spread of an infectious disease. Mathematical Biosciences.214(1):70-2.

95. Shi Z, Fang Y. Temporal relationship between outbound traffic from Wuhan and the 2019 coronavirus disease (COVID-19) incidence in China. medRxiv. 2020:2020.03.15.20034199.

96. Song PX, Wang L, Zhou Y, He J, Zhu B, Wang F, et al. An epidemiological forecast model and software assessing interventions on COVID-19 epidemic in China. medRxiv. 2020:2020.02.29.20029421.

97. St. John RK, King A, De Jong D, Bodie-Collins M, Squires SG, Tam TWS. Border screening for SARS. Emerging Infectious Diseases. 2005;11(1):6-10.

98. Su L, Hong N, Zhou X, He J, Ma Y, Jiang H, et al. Evaluation of the secondary transmission pattern and epidemic prediction of COVID-19 in the four metropolitan areas of China. medRxiv. 2020.

99. Tang B, Wang X, Li Q, Bragazzi NL, Tang S, Xiao Y, et al. Estimation of the Transmission Risk of the 2019-nCoV and Its Implication for Public Health Interventions. Journal of Clinical Medicine.9(2):07.

100. Tian H, Liu Y, Li Y, Wu C-H, Chen B, Kraemer MUG, et al. The impact of transmission control measures during the first 50 days of the COVID-19 epidemic in China. medRxiv. 2020:2020.01.30.20019844.

101. Tsuboi M, Hachiya M, Noda S, Iso H, Umeda T. Epidemiology and quarantine measures during COVID-19 outbreak on the cruise ship Diamond Princess docked at Yokohama, Japan in 2020: a descriptive analysis. Global Health & Medicine. 2020:2020.01037-2020.

102. Wang F, Li Y, Tang D, Li Q, Liu G, Wang J, et al. Trend of the Coronavirus Disease-2019 Epidemic in China After the Lockdown of Wuhan City on January 23, 2020. SSRN. 2020.

103. Wang L, Zhang Y, Huang T, Li X. Estimating the value of containment strategies in delaying the arrival time of an influenza pandemic: a case study of travel restriction and patient isolation. Physical Review E Statistical, Nonlinear, & Soft Matter Physics. 2012;86(3):032901.

104. Wang TH, Wei KC, Hsiung CA, Maloney SA, Eidex RB, Posey DL, et al. Optimizing severe acute respiratory syndrome response strategies: lessons learned from quarantine. American Journal of Public Health. 2007;97:S98-100.

105. Wang X, Liu S, Wang L, Zhang W. An Epidemic Patchy Model with Entry-Exit Screening. Bulletin of Mathematical Biology. 2015;77(7):1237-55.

106. Wells CR, Sah P, Moghadas SM, Pandey A, Shoukat A, Wang Y, et al. Impact of international travel and border control measures on the global spread of the novel 2019 coronavirus outbreak. Proc Natl Acad Sci U S A. 2020;117(13):7504-9.

107. Weng W, Ni S. Evaluation of containment and mitigation strategies for an influenza A pandemic in China. Simulation. 2015;91(5):407-16.

108. Wilder-Smith A, Goh KT, Paton NI. Experience of Severe Acute Respiratory Syndrome in Singapore: Importation of Cases, and Defense Strategies at the Airport. Journal of Travel Medicine. 2003;10(5):259-62.

109. Wood JG, Zamani N, Raina MacIntyre C, Becker NG. Effects of internal border control on spread of pandemic influenza. Emerging Infectious Diseases. 2007;13(7):1038-45.

110. Yang Z, Zeng Z, Wang K, Wong SS, Liang W, Zanin M, et al. Modified SEIR and AI prediction of the epidemics trend of COVID-19 in China under public health interventions. Journal of Thoracic Disease. 2020;12(3):165-74.

111. Ying S, Li F, Geng X, Li Z, Du X, Chen H, et al. Spread and control of COVID-19 in China and their associations with population movement, public health emergency measures, and medical resources. medRxiv. 2020:2020.02.24.20027623.

112. Yu H, Cauchemez S, Donnelly CA, Zhou L, Feng L, Xiang N, et al. Transmission dynamics, border entry screening, and school holidays during the 2009 influenza A (H1N1) pandemic, China. Emerging Infectious Diseases. 2012;18(5):758-66.

113. Yuan H-Y, Hossain MP, Tsegaye MM, Zhu X, Jia P, Wen T-H, et al. Estimating the risk on outbreak spreading of 2019-nCoV in China using transportation data. medRxiv. 2020:2020.02.01.20019984.

114. Yuan Z, Xiao Y, Dai Z, Huang J, Chen Y. A simple model to assess Wuhan lock-down effect and region efforts during COVID-19 epidemic in China Mainland. medRxiv. 2020a;2020a:2020.02.29.20029561.

115. Zhang B, Zhou H, Zhou F. Study on SARS-COV-2 transmission and the effects of control measures in China. medRxiv. 2020a:2020.02.16.20023770.

116. Zhang C, Chen C, Shen W, Tang F, Lei H, Xie Y, et al. Impact of population movement on the spread of 2019-nCoV in China. Emerging Microbes & Infections. 2020:1-28.

117. Zhang N, Zhao PC, Li YG. Increased infection severity in downstream cities in infectious disease transmission and tourists surveillance analysis. Journal of Theoretical Biology. 2019;470:20-9.

118. Zhang WD, Zu ZH, Xu Q, Xu ZJ, Liu JJ, Zheng T. Optimized Strategy for the Control and Prevention of Newly Emerging Influenza Revealed by the Spread Dynamics Model. PLoS ONE. 2014;9(1):11.

119. Zhang Y, Yang P, Liyanage S, Seale H, Deng Y, Pang X, et al. The characteristics of imported cases and the effectiveness of outbreak control strategies of pandemic influenza A (H1N1) in China. Asia-Pac J Public Health. 2012;24(6):932-9.

120. Zhao S, Chen H. Modeling the epidemic dynamics and control of COVID-19 outbreak in China. Quantitative Biology. 2020:1-9.

121. Zhou X, Wu Z, Yu R, Cao S, Fang W, Jiang Z, et al. Modeling-based evaluation of the effect of quarantine control by the Chinese government in the coronavirus disease 2019 outbreak. medRxiv. 2020:2020.03.03.20030445.

122. Zlojutro A, Rey D, Gardner L. A decision-support framework to optimize border control for global outbreak mitigation. Sci Rep. 2019;9(1).

**S6:** Interventions considered in the included studies

| Study ID | Short description of intervention | Phase of implementation |
|---|---|---|
| **Border closure** | | |
| **Studies assessing COVID-19** | | |
| Banholzer 2020 | Closure of national borders for individuals | Local transmission phase |
| **Studies assessing a hypothetical disease (COVID-19 relevant)** | | |
| Boyd 2018 | n.r. | Early phase |
| Boyd 2017 | Closure of national borders (ending the arrival of all commercial passenger planes, private planes, cruise ships, and yachts) | Early phase |
| **Entry/exit screening** | | |
| **Studies assessing COVID-19** | | |
| Clifford 2020 | · Entry screening (at departure)<br>· Exit screening (on arrival)<br>· Follow-up quarantine of positive cases<br>· Follow-up information/handouts to all arriving passengers | Early phase |
| Gostic 2020 | · Entry screening (all arriving passengers; temperature screening followed by a questionnaire on exposure risk)<br>· Exit screening (all departing passengers; temperature screening followed by a questionnaire on exposure risk)<br>· Follow up detainment of potentially infected passengers | Unclear phase |

| Study ID | Short description of intervention | Phase of implementation |
|---|---|---|
| Mandal 2020 | · Entry screening (passengers from China, Hong Kong, Singapore, Thailand, Japan, South Korea, Iran, and Italy; focus on temperature screening; advise for symptomatic passengers to volunteer for screening)<br>· Follow-up quarantine of positive cases | Early phase |
| **Studies assessing influenza** | | |
| Fujita 2011 | · Entry screening (on-board screening at Narita International Airport; questionnaire and thermography)<br>· Follow-up medical assessment (rapid tests followed by RT-PCR)<br>· Follow-up quarantine of positive cases and those sitting nearby<br>· Follow-up daily monitoring of travelers with negative screening results | Unclear phase |
| Gunaratnam 2014 | · Entry screening (all international arrivals at Sydney Airport; health declaration and thermal scanning)<br>· Follow-up medical assessment (swab taken) | Unclear phase |
| Hale 2012 | · Entry screening (all international arrivals at Auckland International Airport; health card, self-presentation)<br>· Follow up medical assessment (ILI case definition, RT-PCR)<br>· Quarantine of positive cases | Unclear phase |
| Hien 2010 | · Entry screening (all international arrivals; temperature screening; symptom questionnaire)<br>· Follow up quarantine of suspected cases (in hospitals) | Local transmission phase |
| Khan 2013 | · Entry screening (targeted: passengers arriving at international airports after flying from Mexico on a direct flight or indiscriminate entry screening on a direct or a connecting flight)<br>· Exit screening (passengers departing from any international airport in Mexico) | Unclear phase |
| Kuo 2009 | · Entry screening (on-board screening of passengers arriving from epidemic affected areas, such as Mexico, USA, and Canada before passengers leave their seats; the procedure soon adjusted for only those airplanes reporting ill passengers on board)<br>· Follow-up quarantine of positive cases (hospital) | Unclear phase |

| Study ID | Short description of intervention | Phase of implementation |
|---|---|---|
| Malone 2009 | · Entry screening (all international passengers arriving at 18 US airports from Asia, Europe, South America and Canada: includes pre-arrival, primary active, and secondary active surveillance measures)<br>· Follow up quarantine of positive cases | Local transmission phase |
| Mummert 2013 | · Entry screening (thermal scanners)<br>· Follow-up quarantine of suspected cases and those seating nearby | Local transmission phase |
| Priest 2013 | · Entry screening (for all passengers arriving from Australia; all completed a health questionnaire, randomly chosen passengers (50%) screened for temperature and had throat and nasal swabs taken ) | Unclear phase |
| Sakaguchi 2012 | · Entry screening (all passengers arriving from other countries; infrared thermos-scanner, a health declaration form)<br>· Follow-up medical assessment (rapid influenza test, RT-PCR)<br>· Follow-up quarantine of positive cases and close contacts (seating next to them in the airplane)<br>· Follow-up information/handouts (all asymptomatic passengers)<br>· Follow-up health monitoring for fellow travelers of infected individuals identified during entry screening | Unclear phase |
| Wang 2015 | Different scenarios were considered in the model:<br>· Indiscriminate entry and exit screening in each patch<br>· Indiscriminate entry screening only in each patch<br>· Targeted entry screening (travel to and from high risk patch only)<br>· Selective entry screening (only the high risk patch and those closely connected patches)<br>· One-way entry screening in low-risk patches only | Local transmission phase |
| Yu 2012 | · Entry screening (all passengers at any point of entry into China from another country or from a neighboring region)<br>· Follow-up quarantine of suspected cases (in designated hospitals) | Local transmission phase;<br>Unclear phase |
| Zhang 2014 | · Entry screening (temperature screening at ports of entry) | Early phase |
| Zhang 2012 | · Entry screening (temperature screening, questionnaire)<br>· Follow-up medical assessment (designated hospital)<br>· Quarantine of positive cases and close contacts | Unclear phase |

| Study ID | Short description of intervention | Phase of implementation |
|---|---|---|
| | · Medical follow-up of all travelers from overseas (daily contacts for 7 days)<br>· Follow-up ILI screening in hospitals | |
| Zlojutro 2018 | · Entry screening (in airports)<br>· Follow up quarantine of positive cases | Early phase |
| **Studies assessing SARS** | | |
| Anonymous | · Entry screening (all passengers arriving from affected areas; questionnaire, thermal image scanners)<br>· Exit screening (all departing passengers; thermal image scanners)<br>· Follow-up medical assessment (oral temperature screening, standardized questionnaire for SARS) | Local transmission phase;<br>Unclear phase |
| Glass 2006 | · Border screening (health declaration cards)<br>· Follow-up Information/handouts to travelers | Early phase;<br>Unclear phase |
| Goubar 2008 | · Entry screening (for symptomatic travelers only) | Early phase;<br>Unclear phase |
| Samaan 2004 | · Entry screening (identification through self-referral, detection by airline staff or airport custom staff; Australian Quarantine and Inspection Service conducting basic screening)<br>· Follow up screening (temperature screening by nursing staff)<br>· Follow-up screening (chief quarantine officer applies case definition)<br>· Follow-up medical assessment (in a hospital) | Early phase;<br>Local transmission phase |
| Pang 2003 | · Border screening (at the Beijing airport, major train stations, and 71 roads connecting Beijing to other areas) | Local transmission phase |
| Sang 2012 | · Entry screening | Unclear phase |
| St. John 2005 | · Entry screening (all arriving passengers; questionnaire, thermal scanners)<br>· Exit screening (all departing passengers; questionnaire, thermal scanners)<br>· Follow-up: Information/handouts to all arriving passengers<br>· Follow-up medical assessment<br>· Detainment and further medical assessment (in a hospital) | Unclear phase |

| Study ID | Short description of intervention | Phase of implementation |
|---|---|---|
| Wilder-Smith 2003 | Different entry and exit screening procedures rolled out at different times:<br>· Entry screening (all passengers arriving from SARS-affected areas; visual screening and further temperature screening; health declaration)<br>· Entry screening (all arriving passengers in the airport and road entry points and further ferry terminals; temperature screening)<br>· Exit screening (all departing passengers in the airport and road entry points and further ferry terminals; temperature screening)<br>· Entry screening (all arriving passengers screened for SARS)<br>· Follow-up medical assessment | Early phase; (or post-peak phase) |
| **Studies assessing MERS** | | |
| Muraduzzaman, 2017 | · Entry screening (Bangladeshi travelers/pilgrims returning from the Middle East)<br>· Follow-up medical assessment<br>· Follow up quarantine of suspected cases | Unclear phase |
| **Studies assessing multiple diseases (SARS, MERS, and/or Influenza)** | | |
| Gostic 2015 | · Entry screening (all arriving passengers; temperature screening followed by a questionnaire on exposure risk)<br>· Exit screening (all departing passengers; temperature screening followed by a questionnaire on exposure risk)<br>· Follow up detainment of potentially infected passengers | Unclear phase |
| Pitman 2005 | · Entry screening | Unclear phase |
| **Studies assessing a hypothetical disease (COVID-19 relevant)** | | |
| Kim 2017 | n.r. | Unclear phase |
| Liu 2006 | · Entry screening<br>· Exit screening<br>· Follow-up quarantine of positive cases | Local transmission phase |
| **Travel ban** | | |

| Study ID | Short description of intervention | Phase of implementation |
|---|---|---|
| **Studies assessing COVID-19** | | |
| Adekunle 2020 | Ban on China, Iran, South Korea, and Italy | Local transmission phase |
| Chiyomaru 2020 | Three scenarios considered:<br>· Global ban<br>· Global quarantine<br>· Non-global restrictions | Local transmission phase |
| Costantino 2020 | Three scenarios modelled:<br>· No ban<br>· Current ban followed by a full lifting<br>· Partial lifting of the current ban (allowing over 100,000 university students to enter Australia, but not tourists) | Local transmission phase;<br>Post-peak phase |
| Mbuvha 2020 | n.r. | Local transmission phase |
| Mondal 2020 | Shutting of airlines and rail transport in India | Local transmission phase |
| Pullano 2020 | Ban on international flights in Hubei (as an example) | Early phase |
| Ray 2020 | · Main scenario: national lockdown<br>· Alternative scenario A: mild action: social distancing and travel ban<br>· Alternative scenario B: no action | Local transmission phase |
| **Studies assessing influenza** | | |
| Hsieh 2007 | n.r. | Local transmission phase |
| **Travel-related quarantine** | | |
| **Studies assessing COVID-19** | | |

| Study ID | Short description of intervention | Phase of implementation |
|---|---|---|
| Moriatry 2020 | Quarantine of travelers (14 days either on the ship or on the land (port of Oakland) before disembarkation or onward travel) | Local transmission phase |
| Tsuboi 2020 | Quarantine of all travelers in their cabins (followed by targeted screening for COVID-19) | Unclear phase |
| **Studies assessing influenza** | | |
| Nishiura 2009 | Quarantine of travelers (all individuals arriving from the country in which the outbreak occurred) | Early phase |
| **Studies assessing SARS** | | |
| de Vlas 2009 | Quarantine of travelers (in airports and stations) | Early phase;<br>Local transmission phase;<br>Post-peak phase |
| Hsieh 2006 | Home quarantine of travelers (anyone who entered the country from affected countries) | Local transmission phase |
| Wang 2007 | Quarantine of travelers (those sitting on the same flight within 3 rows of a person infected with SARS or those returning from WHO–designated SARS-affected areas) | Unclear phase |
| **Travel restrictions** | | |
| **Studies assessing COVID-19** | | |
| Chang 2020 | n.r. | Early phase;<br>Local transmission phase |
| Odendaal 2020 | n.r. | Early phase |

| Study ID | Short description of intervention | Phase of implementation |
|---|---|---|
| Scala 2020 | n.r. | Early phase;<br>Local transmission phase;<br>Post-peak phase |
| **Studies assessing influenza** | | |
| Bolton 2012 | n.r. | Local transmission phase |
| Chong 2012 | Restricting air, land, and maritime travel | Local transmission phase |
| Ciofi 2008 | Restricting air travel | Early phase |
| Colizza 2007 | Restricting air travel | Early phase |
| Cooper 2005 | Restricting air travel | Local transmission phase |
| Eichner 2009 | Restricting air travel | Early phase |
| Epstein 2007 | Restricting air travel | Early phase |
| Flahault 2006 | Restricting air travel | Early phase |
| Germann 2006 | Restricting air travel | Local transmission phase |
| Kerneis 2008 | Restricting air travel | Early phase |
| Lam 2011 | n.r. | Early phase |
| Lee 2012 | n.r. | Early phase |
| Marcelino 2013 | Restricting air travel | Early phase;<br>Local transmission phase |
| Nigmatulina 2009 | n.r. | Early phase;<br>Local transmission phase |

| Study ID | Short description of intervention | Phase of implementation |
|---|---|---|
| Scalia Tomba 2008 | n.r. | Early phase |
| Wang 2012 | Cancellation of non-essential trips | Early phase |
| Weng 2015 | n.r. | Early phase;<br>Local transmission phase |
| Wood 2007 | Restricting air travel | Early phase |
| **Studies assessing multiple diseases (SARS and influenza)** | | |
| Chung 2015 | Restricting (control measures on) air travel | Unclear phase |
| Hollingsworth 2006 | Restricting air travel | Early phase |
| **Studies assessing a hypothetical disease (COVID-19 relevant)** | | |
| Arino 2007 | n.r. | Early phase |
| Nakata 2015 | n.r. | Early phase;<br>Local transmission phase |
| **Multiple travel-related control measures** | | |
| **Studies assessing COVID-19** | | |
| Aleta 2020 | Lockdown of Wuhan (referred in the study as "Wuhan's quarantine*")* | Local transmission phase |
| Anzai 2020 | "Travel restriction and border control have been implemented by various countries, either as: (i) complete travel bans, (ii) travel restriction and quarantine—which allows for restriction of healthy individuals, (iii) entry screening for all incoming travelers, or some combination thereof" | Local transmission phase |
| Arima 2020 | Entry/exit screening | Unclear phase |

| Study ID | Short description of intervention | Phase of implementation |
|---|---|---|
| | · Entry screening (repatriation of nationals from China and oropharyngeal swab sample taken for all)<br>· Follow-up quarantine of positive cases (in a hospital)<br>Travel-related quarantine (all returning from China for 14 days) | |
| Cheng 2020 | Entry/exit screening<br>· Entry screening (on-board temperature screening for all travelers from Wuhan)<br>· Follow-up medical assessment (in designated hospitals)<br>Travel-related quarantine (all returning from Wuhan, Guangdong, Zheijiang, China, Hong Kong, Macau, and later from South Korea and Italy) | Local transmission phase |
| Chinazzi 2020 | n.r. | Local transmission phase |
| Cowling 2020 | Travel ban<br>· Ban on Hubei residents and visitors to Hubei province in the past 14 days<br>· Ban on flights (between Hong Kong and Wuhan)<br>· Ban on entry of non-Hong Kong residents from overseas countries and territories<br>Travel restrictions<br>· Restricting air travel<br>· Restricting land travel<br>Border closure<br>Travel-related quarantine (for travelers from Hubei, then China, South Korea, Iran, Italy, Germany, Japan and Spain, all Schengen area, all overseas countries, Macau and Taiwan). | Local transmission phase |
| Dandekar 2020 | n.r. | Local transmission phase |
| Ediriweera 2020 | · Lockdown (island-wide)<br>· Travel-related quarantine (all inbound passengers) | Local transmission phase |
| Fang 2020 | Lockdown of Wuhan | Local transmission phase |
| Hamidouche 2020 | Travel ban<br>· Ban: air travel (China, Italy, Morocco, France)<br>· Ban: local public transport including rail traffic | Local transmission phase |



Movsisyan A, et al. BMJ Open 2021; 11:e041619. doi: 10.1136/bmjopen-2020-041619

| Study ID | Short description of intervention | Phase of implementation |
|---|---|---|
| | Closure of all land borders and air routes<br>Travel-related quarantine (14 days) | |
| He 2020 | n.r. | Local transmission phase |
| Hossain 2020 | Lockdown of Wuhan (referred in the study as "lockdown, border control, or quarantine measures"). | Local transmission phase |
| Hou 2020 | Lockdown of Wuhan | Local transmission phase |
| Jia 2020 | Lockdown of Wuhan (referred in the study as "quarantine of Wuhan") | Early phase;<br>Local transmission phase |
| Jiang 2020 | n.r. (referred in the study as “travel restrictions in Hubei”) | Local transmission phase |
| Kraemer 2020 | Lockdown of Wuhan (referred in the study as "cordon sanitaire imposed on Wuhan") | Local transmission phase |
| Lai 2020 | Lockdown of Wuhan | Early phase;<br>Local transmission phase |
| Lau 2020 | Lockdown of Wuhan | Early phase;<br>Local transmission phase |
| Li 2020 | Lockdown of Wuhan (referred in the study as "quarantine and traffic blockage") | Early phase;<br>Local transmission phase |
| Lin 2020 | Lockdown of Wuhan (referred in the study as "travel bans into and from Wuhan") | Local transmission phase |
| Linka 2020 | n.r. | Early phase;<br>Local transmission phase |
| Liu 2020 | Lockdown of Wuhan (referred in the study as "city closure") | Early phase;<br>Local transmission phase |

| Study ID | Short description of intervention | Phase of implementation |
|---|---|---|
| Liu 2020a | Lockdown of Wuhan and travel restrictions in other cities | Early phase;<br>Local transmission phase |
| Pan 2020 | Various interventions strategies in China, including travel restrictions and voluntary quarantine | Local transmission phase |
| Pan 2020a | “Strict traffic restrictions” implemented by the Chinese government. | Local transmission phase |
| Pinkas 2020 | · Border screening (passengers location cards, temperature screening)<br>· Closure of borders to non-citizens<br>· Travel ban: all non-essential travel; suspension of all international air and rail services<br>· Travel-related quarantine | Local transmission phase |
| Qiu 2020 | · Lockdown of Wuhan and other cities in Hubei<br>· Imposed travel restrictions, ranging from "canceling public events, stopping public transportation to limiting how often residents can leave home" | Early phase;<br>Local transmission phase |
| Quilty 2020 | · Lockdown of Wuhan (referred in the study as "cordon sanitaire on the city of Wuhan")<br>· Travel restrictions extended to the whole of Hubei | Local transmission phase |
| Shi 2020 | Lockdown of Wuhan (referred in the study as "Wuhan travel ban") | Local transmission phase |
| Song 2020 | Lockdown of Wuhan (referred in the study as "community-level quarantines such as hospitalization for infected cases, city blockade, traffic control and restricted social activities, and so on") | Local transmission phase |
| Su 2020 | “Official quarantine regulations and travel restrictions in China” | Local transmission phase |
| Tang 2020 | Lockdown of Wuhan and other cities in China (referred in the study as "travel restrictions affecting five cities (Wuhan, Huanggang, Ezhou, Chibi and Zhijiang), effectively shutting down the movement of more than 40 million people") | Local transmission phase |
| Tian 2020 | Lockdown of Wuhan and other cities in China ("China banned travel to and from Wuhan city on 23 January and implemented a national emergency response") | Local transmission phase |

| Study ID | Short description of intervention | Phase of implementation |
|---|---|---|
| Wang 2020 | Lockdown of Wuhan (referred in the study as "quarantine of Wuhan city") | Local transmission phase |
| Wells 2020 | · Lockdown of Wuhan and some other cities in China<br>· Entry/exit screening in airports (questionnaire, self-reporting) | Local transmission phase |
| Yang 2020 | Lockdown of Wuhan and other cities in China (referred in the study as "large-scale quarantine, strict controls on travel and extensive monitoring of suspected cases) | Local transmission phase |
| Ying 2020 | Lockdown of Wuhan and other cities in Hubei | Local transmission phase |
| Yuan 2020 | Lockdown of Wuhan<br>Two scenarios were considered:<br>· Border control measures without any other control (no mask wearing, isolation, or contact tracing)<br>· Border control with some other control measures | Early phase;<br>Local transmission phase |
| Yuan 2020a | Lockdown of Wuhan "combined with nationwide traffic restrictions" | Local transmission |
| **Studies assessing influenza** | | |
| Bajardi 2011 | Multiple scenarios modelled:<br>· No travel restrictions<br>· Travel ban: air travel (country-specific)<br>· Travel restrictions: land travel | Early phase |
| Caley 2007 | Entry/exit screening<br>· Exit screening (all passengers at departure)<br>· Follow up detainment (those positive are detained and prevented from traveling)<br>· Entry screening (all passengers at arrival)<br>· Follow-up quarantine of all travelers if a positive case is detected<br>Travel restriction: air travel | Early phase |
| Ferguson 2006 | Border controls<br>Travel restriction | Local transmission phase |
| Zhang 2019 | Entry/exit screening | Early phase |

| Study ID | Short description of intervention | Phase of implementation |
|---|---|---|
| | · Entry screening (city-entry surveillance)<br>· Exit screening (city-exit surveillance)<br>· Follow up quarantine of positive cases<br>Travel reduction across cities (decrease in human mobility) | |
| Zhang 2020 | Lockdown of Wuhan and other cities in Hubei (referred in the study as "citywide quarantine of Wuhan and several nearby cities") | Local transmission phase |
| Zhang 2020a | Lockdown of Wuhan and other cities in Hubei ("Chinese government and people moved quickly to take measures to control the sources of infection and block the routes of transmission") | Early phase;<br>Local transmission phase |
| Zhao 2020 | Lockdown of Wuhan (referred to in the study as "a quarantine") | Early phase;<br>Local transmission phase;<br>Post-peak phase |
| Zhou 2020 | Lockdown of Wuhan (referred in the study as "strict control policies, including lockdown of Wuhan city") | Local transmission phase |
| **Studies assessing multiple diseases (COVID-19 and influenza)** | | |
| Kong 2020 | Lockdown of Wuhan ("Wuhan was locked down and China raised the national public health response to the first state of emergency") | Local transmission phase |
| **Studies assessing a hypothetical disease (COVID-19 relevant)** | | |
| Malmberg 2020 | · Inflow travel restrictions<br>· Quarantine of travelers (all those arriving) | Local transmission phase;<br>Post-peak phase |



Movsisyan A, *et al. BMJ Open* 2021; 11:e041619. doi: 10.1136/bmjopen-2020-041619

**S7:** Outcome categories, types and specific outcomes assessed in studies

| Type | Outcomes included | Number of studies |
|---|---|---|
| **Category 1. Infectious disease outcomes** | | |
| Number or proportion of cases | Number of cases (predicted, reported, detected) | 58 |
| | Number of cases as rates per e.g. 1000, 100000 population | 3 |
| | Number of imported/exported cases | 4 |
| | Number of cases at epidemic peak | 1 |
| | Attack rate | 7 |
| Number or proportion of deaths | Number of deaths | 6 |
| | Case fatality rate | 1 |
| Healthcare resources | Number of cases requiring ICU treatment | 1 |
| | Time until ICU capacity is reached | 1 |
| Reproduction number | Effective reproduction number | 12 |
| | Basic reproduction number | 5 |
| Temporal development of epidemic | Time to epidemic arrival | 22 |
| | Time to epidemic peak | 9 |
| | Time to 1000 cases | 2 |
| | Doubling rate | 1 |
| Probability of epidemic | Probability of epidemic | 4 |
| | Probability of avoiding epidemic | 1 |
| | Probability of sustained transmission | 1 |
| | Probability of releasing infected individuals | 1 |
| | Probability of developing symptoms | 1 |
| Other infectious disease outcomes | Transmission risk index | 1 |
| | Transmission routes | 1 |
| | Number of infected cities | 1 |
| **Category 2. Screening outcomes** | | |
| Detection of high risk persons or cases | Number or proportion screened that are identified as high risk | 10 |
| | Number or proportion screened that are diagnosed | 12 |
| | Number or proportion quarantined that are diagnosed | 3 |
| | Positive predictive value of screening measures/tests | 2 |
| | Probability of missing cases | 4 |
| **Category 3. Economic outcomes** | | |
| Costs | Value of measures | 2 |
| | Cost of measures as percentage of Gross Domestic Product | 1 |
| | Cost of preventing cases | 1 |
| Industry impact | Air traffic passenger volumes | 1 |
| **Category 4. Social outcomes** | | |
| Acceptability of measures | Attitudes towards travel restrictions | 1 |



Movsisyan A, *et al. BMJ Open* 2021; 11:e041619. doi: 10.1136/bmjopen-2020-041619

**S8:** Overview of types of studies included in the evidence map

### Inferential studies

**Compartmental models**

Common in infectious disease research, these include, for example the SIR model (comprising three compartments, S: susceptible, I: infectious, R: recovered). Several included studies applied such models, and extended the SIR model by adding other compartments, such as E: exposed, NS: non-susceptible, L: latent, I: isolated, D: dead

Examples from included studies: Kim et al. 2017; Mandal et al. 2020; Mbuvha et al. 2020

**Spatial models**

Several included studies applied models to explore how disease transmission moves spatially. Such models use assumptions and/or data about how humans move to predict disease transmission. Included studies examined, for example, mobility data from smartphone usage or global flight patterns.

Examples from included studies: Bajardi et al. 2011; Chinazzi et al. 2020; Clifford et al. 2020; Colizza 2020; Kraemer et al. 2020; Pullano et al. 2020

**Epidemiological time-series models**

Many included studies investigated the temporal nature of the disease data, and applied time-series approaches common to epidemiology. They applied a range of approaches, including simple time-series analysis, auto-regressive integrated moving average models, and change-point analysis to explore changes over time.

Examples from included studies: Chung et al. 2015; Jiang et al. 2020; Lin 2020_trends; Yuan 2020a

**Other epidemiological techniques**

Included studies also used more simple epidemiological designs and and statistical methods, such as correlation analysis, linear regression and statistical testing (e.g. chi-square test).

Examples from included studies: Fujita et al. 2011; Lau et al. 2020; Wang et al. 2020d

**Mixed approaches**

Several included studies used a range of approaches; combinations included compartmental models and spatial models, compartmental models and time-series models.

Examples from included studies: Epstein et al. 2007; Linka et al. 2020; Longxiang et al. 2020; Marcelino et al. 2013; Wang et al. 2020

### Descriptive studies

**Observational studies**

Several included studies described the impact of control measures through summary statistics; these comprised, for example, the number or proportion of screened individuals that were classified as high risk and the number or proportion of screened individuals that were diagnosed.

Examples from included studies: Arima et al. 2020; Hale et al. 2020; Kuo et al. 2009; Sakaguchi et al. 2012

**Graphical summary studies**
A small number of included studies used graphical summary techniques to describe the impact of control measures. These illustrated, for example, how the number of cases developed in time relative to the introduction of control measures.

Examples from included studies: Cheng et al. 2020; de Vlas et al. 2009; Pinkas et al. 2020



Movsisyan A, *et al. BMJ Open* 2021; 11:e041619. doi: 10.1136/bmjopen-2020-041619
