# Supplementary material for: Travel-related control measures to contain the COVID-19 pandemic: an evidence map
Source: BMJ Open. 2021 Apr 9;11(4):e041619. doi: 10.1136/bmjopen-2020-041619 (PMC8042592; doi:10.1136/bmjopen-2020-041619)
Supplement: Author's manuscript [file bmjopen-2020-041619.draft_revisions.pdf]

BMJ Open is committed to open peer review. As part of this commitment we make the peer review history of every article we publish publicly available.

When an article is published we post the peer reviewers' comments and the authors' responses online. We also post the versions of the paper that were used during peer review. These are the versions that the peer review comments apply to.

The versions of the paper that follow are the versions that were submitted during the peer review process. They are not the versions of record or the final published versions. They should not be cited or distributed as the published version of this manuscript.

BMJ Open is an open access journal and the full, final, typeset and author-corrected version of record of the manuscript is available on our site with no access controls, subscription charges or pay-per-view fees (http://bmjopen.bmj.com).

If you have any questions on BMJ Open's open peer review process please email info.bmjopen@bmj.com

BMJ Open

# Travel-related control measures to contain the COVID-19 pandemic: an evidence map



| | |
|---|---|
| Journal: | *BMJ Open* |
| Manuscript ID | bmjopen-2020-041619 |
| Article Type: | Original research |
| Date Submitted by the Author: | 13-Jun-2020 |





| | |
|---|---|
| Complete List of Authors: | Burns, Jacob; Ludwig Maximilians University Munich, Institute for Medical Information Processing, Biometry and Epidemiology; Ludwig Maximilians University Munich, Pettenkofer School of Public Health<br>Movsisyan, Ani; Ludwig Maximilians University Munich, Institute for Medical Information Processing, Biometry and Epidemiology; Ludwig Maximilians University Munich, Pettenkofer School of Public Health<br>Biallas, Renke; Ludwig Maximilians University Munich, Institute for Medical Information Processing, Biometry and Epidemiology; Ludwig Maximilians University Munich, Pettenkofer School of Public Health<br>Coenen, Michaela; Ludwig Maximilians University Munich, Institute for Medical Information Processing, Biometry and Epidemiology; Ludwig Maximilians University Munich, Pettenkofer School of Public Health<br>Geffert, Karin; Ludwig Maximilians University Munich, Institute for Medical Information Processing, Biometry and Epidemiology; Ludwig Maximilians University Munich, Pettenkofer School of Public Health<br>Horstick, Olaf; Heidelberg University, Heidelberg Institute of Global Health<br>Klerings, Irma; Danube University Krems, Department for Evidence-based Medicine and Evaluation<br>Pfadenhauer, Lisa; Ludwig Maximilians University Munich, Institute for Medical Information Processing, Biometry and Epidemiology; Ludwig Maximilians University Munich, Pettenkofer School of Public Health<br>von Philipsborn, Peter; Ludwig Maximilians University Munich, Institute for Medical Information Processing, Biometry and Epidemiology; Ludwig Maximilians University Munich, Pettenkofer School of Public Health<br>Sell, Kerstin; Ludwig Maximilians University Munich, Institute for Medical Information Processing, Biometry and Epidemiology; Ludwig Maximilians University Munich, Pettenkofer School of Public Health<br>Strahwald, Brigitte; Ludwig Maximilians University Munich, Institute for Medical Information Processing, Biometry and Epidemiology; Ludwig Maximilians University Munich, Pettenkofer School of Public Health<br>Stratil, Jan; Ludwig Maximilians University Munich, Institute for Medical Information Processing, Biometry and Epidemiology; Ludwig Maximilians University Munich, Pettenkofer School of Public Health<br>Voss, Stephan; Ludwig Maximilians University Munich, Institute for Medical Information Processing, Biometry and Epidemiology; Ludwig Maximilians University Munich, Pettenkofer School of Public Health<br>Rehfuess, Eva; Ludwig Maximilians University Munich, Institute for Medical Information Processing, Biometry and Epidemiology, Pettenkofer School of Public Health; Ludwig Maximilians University Munich, Pettenkofer School of Public Health |

| | |
|---|---|
| Keywords: | Public health < INFECTIOUS DISEASES, INFECTIOUS DISEASES, Diabetic foot < DIABETES & ENDOCRINOLOGY |
| | |

SCHOLARONE™ Manuscripts

*I, the Submitting Author has the right to grant and does grant on behalf of all authors of the Work (as defined in the below author licence), an exclusive licence and/or a non-exclusive licence for contributions from authors who are: i) UK Crown employees; ii) where BMJ has agreed a CC-BY licence shall apply, and/or iii) in accordance with the terms applicable for US Federal Government officers or employees acting as part of their official duties; on a worldwide, perpetual, irrevocable, royalty-free basis to BMJ Publishing Group Ltd ("BMJ") its licensees and where the relevant Journal is co-owned by BMJ to the co-owners of the Journal, to publish the Work in this journal and any other BMJ products and to exploit all rights, as set out in our licence.*

*The Submitting Author accepts and understands that any supply made under these terms is made by BMJ to the Submitting Author unless you are acting as an employee on behalf of your employer or a postgraduate student of an affiliated institution which is paying any applicable article publishing charge ("APC") for Open Access articles. Where the Submitting Author wishes to make the Work available on an Open Access basis (and intends to pay the relevant APC), the terms of reuse of such Open Access shall be governed by a Creative Commons licence – details of these licences and which Creative Commons licence will apply to this Work are set out in our licence referred to above.*

*Other than as permitted in any relevant BMJ Author's Self Archiving Policies, I confirm this Work has not been accepted for publication elsewhere, is not being considered for publication elsewhere and does not duplicate material already published. I confirm all authors consent to publication of this Work and authorise the granting of this licence.*

For peer review only

# Travel-related control measures to contain the COVID-19 pandemic: an evidence map

Jabob Burns*[1,2], Ani Movsisyan*[1,2], Renke Biallas[1,2], Michaela Coenen[1,2], Karin Geffert[1,2], Olaf Horstick[3], Irma Klerings[4], Lisa Maria Pfadenhauer[1,2], Peter von Philipsborn [1,2], Kerstin Sell[1,2], Brigitte Strahwald[1,2], Jan M Stratil[1,2], Stephan Voss[1,2], Eva Rehfuess [1,2]

* These authors have contributed equally.

[1] Institute for Medical Information Processing, Biometry and Epidemiology; Ludwig-Maximilians-University Munich, Germany

[2] Pettenkofer School of Public Health; Ludwig-Maximilians-University, Munich, Germany

[3] Heidelberg Institute of Global Health, Heidelberg University, Germany

[4] Department for Evidence-based Medicine and Evaluation, Danube University Krems, Austria

Corresponding author:
Ani Movsisyan
Institute of Medical Information Processing, Biometry, and Epidemiology
Pettenkofer School of Public Health
LMU Munich, Marchioninistrasse 17, 81377 Munich, Germany
ani.movsisyan@ibe.med.uni-muenchen.de
+49 17685245374

Keywords: Travel restrictions, SARS-CoV-2, COVID-19, pandemic, evidence map.

Word count: 4716

For peer review only

## Abstract

**Objectives**: To comprehensively map the existing evidence assessing the impact of travel-related control measures for containment of the SARS-CoV-2/COVID-19 pandemic.

**Design**: Rapid systematic evidence map.

**Data sources**: MEDLINE, Embase and Web of Science, and COVID-19-specific databases offered by the US Centers for Disease Control and Prevention and the World Health Organization.

**Eligibility criteria**: We included studies in human populations susceptible to SARS-CoV-2/COVID-19, SARS-CoV-1/SARS, MERS-CoV/MERS, or influenza. Interventions of interest were travel-related control measures affecting human travel across national or sub-national borders. Outcomes of interest included infectious disease, screening, other health, economic and social outcomes. We considered all types of empirical studies which quantitatively evaluate impact. We considered studies in Armenian, English, French, German, Italian, and Russian, based on the language capacity of the team.

**Data extraction and synthesis**: We extracted data from included studies in a standardized manner, and mapped them to a priori and (one) post-hoc defined categories.

**Results**: We included 122 studies assessing travel-related control measures. These studies were undertaken across the globe, with most from the Western Pacific Region (n=71). A large proportion of studies focused on COVID-19 (n=59), but a number of studies also examined SARS, MERS, and influenza. We identified studies on border closures (n=3), entry/exit screening (n=31), travel-related quarantine (n=6), travel bans (n=8), and travel restrictions (n=25). Many addressed a bundle of travel-related control measures (n=49). Most studies assessed infectious disease (n=98) and/or screening-related (n=25) outcomes; we found only limited evidence on economic and social outcomes. Studies applied numerous methods, both inferential and descriptive in nature, ranging from simple observational methods to complex modeling techniques.

**Conclusions**: We identified a broad, heterogeneous and complex evidence base on travel-related control measures. Decisions and future research on these measures will need to attend to these challenges, as well as the differences in study methodology, quality, and reporting.



For peer review only - http://bmjopen.bmj.com/site/about/guidelines.xhtml
For peer review only

**Strengths and limitations of this study**

- We applied systematic and standardized methods at all stages, and have produced a comprehensive evidence map of travel-related control measures for containment of the SARS-CoV-2/COVID-19 pandemic.
- For title/abstract and full text screening, we developed guidance documents and conducted piloting exercises to ensure consistency among review authors; we additionally collected and clarified all uncertainties on a rolling basis through daily team calls.
- We conducted searches in three major health-related databases and two COVID-specific databases; however, it is likely that most studies assessing economic and social outcomes are found in other databases.
- The unspecific and inconsistent reporting of primary studies with regard to interventions, especially when a package of control measures was investigated, meant that determining eligibility, as well as summarizing and mapping these studies was challenging.
- We did not include travel warning or travel advice in the evidence map, which limits the scope of this map.

For peer review only


For peer review only - http://bmjopen.bmj.com/site/about/guidelines.xhtml

## Introduction

In December 2019 the occurrence of the novel coronavirus, SARS-CoV-2, was reported in Wuhan, China. Over the next weeks the virus and the associated respiratory disease referred to as COVID-19 spread further into China and other parts of Asia including Japan, South Korea, and Thailand (1). By mid-March 2020, when the World Health Organization (WHO) declared COVID-19 a global pandemic, cases had been observed in over 100 countries and territories across the globe (2).

According to WHO, various travel-related control measures, such as entry/exit screening, travel bans to and/or from specific areas, and quarantine of travelers have since been implemented by most countries around the world to contain and mitigate the spread of SARS-CoV-2 and COVID-19 (3). Following the early-stage responses in Asian countries, strict measures, such as border closures and drastic reductions in airline travel, have been put into place in most countries around the world, starting in February 2020 and continuing into May 2020. As this pandemic develops further, especially given the current lack of a vaccination or effective treatment, it is important that decision-makers in varying contexts have access to the best-available evidence regarding such travel-related control measures. This will help inform whether the currently implemented measures should be sustained, adapted, or lifted. Such decisions will need to be based on evidence regarding the effectiveness of these measures in contributing to the control of the pandemic, as well as regarding the associated economic and social impacts. They will also need to take into account short- and longer-term costs, acceptability, and feasibility of such measures.

Travel-related control measures have been assessed through systematic or narrative reviews in the context of previous epidemics and pandemics, such as influenza, severe acute respiratory syndrome (SARS-CoV-1/SARS), and Middle East Respiratory Syndrome (MERS-CoV/MERS). Regarding the containment of influenza, these reviews have examined the effectiveness of a broad set of measures (4-6), as well as the effectiveness of specific measures, such as entry/exit screening (6, 7); they have also assessed the economic implications of various pharmaceutical and non-pharmaceutical interventions during influenza pandemics (8, 9). Effectiveness of measures, such as international travel bans and entry/exit screening have also been examined in reviews of SARS and MERS (7, 10). To date and to the best of our knowledge, the evidence on travel-related control measures for the control of the current pandemic has not been systematically assessed.

In this view, the World Health Organization commissioned us to comprehensively map the existing evidence assessing the impact of travel-related control measures (i.e. border closures, travel restrictions and bans, entry and exit screening, quarantine/isolation of travelers crossing borders, multiple interventions combined) for containment of the SARS-CoV-2/COVID-19 pandemic, drawing on evidence from the current pandemic, as well as on evidence in relation to SARS, MERS, and influenza. This map can serve as an important resource for researchers and policy-makers in providing an overview of the currently available evidence in relation to various travel-related control measures to contain the COVID-19 pandemic. In itself, the evidence map is not sufficient to assess the effectiveness of different measures. Instead, it provides an important basis for decisions regarding the need for and possibility to conduct more specific evidence synthesis (e.g. regarding a specific category of control measures or a specific type of studies).


For peer review only - http://bmjopen.bmj.com/site/about/guidelines.xhtml

# Methods

## Search strategy

We designed the evidence map in accordance with the Preferred Reporting Items for Systematic Reviews and Meta-Analyses (PRISMA) guideline, and implemented the entire project within ten days (see Supplementary file S1 for the completed PRISMA checklist). We searched the following databases: (i) Ovid MEDLINE(R) ALL (1946-present), (ii) Embase.com (Elsevier), (iii) Science Citation Index Expanded (1900-present), Social Sciences Citation Index (1900-present), Emerging Sources Citation Index (2015-present) (Web of Science), (iv) the US Centers for Disease Control and Prevention's COVID-19 Research Articles Downloadable Database (includes published articles, as well as grey literature, such as preprints), and (v) WHO COVID-19 Database (includes published articles only).

The initial search strategy was developed for MEDLINE (see online supplementary file S2) and further adapted for the other databases. All database searches were conducted up to 3 May 2020. The search strategies were designed and conducted by an experienced information specialist. The searches were conducted in English. Where database functionality allowed for it, we limited the search results to Armenian, English, French, German, Italian, and Russian, based on the language capacity of the research team, and considered studies for inclusion published in all of these languages. We also conducted forward and backward citation searches of all relevant (systematic) reviews identified through the database searches (see online supplementary file S3) up to 6 May 2020 in Scopus (Elsevier).

## Eligibility criteria

We determined eligibility of studies based on the investigated population/context, intervention, outcome, and study type.

Regarding the population/context, this evidence map draws on direct evidence from human populations susceptible to the current SARS-CoV-2/COVID-19, as well as indirect evidence from a set of other relevant respiratory infectious diseases. To help define a set of diseases that is most similar and relevant to COVID-19, we used the following criteria: (i) diseases of viral origin; (ii) mode of transmission primarily airborne via droplets/aerosols (as well as person-to-person); (iii) acute disease with the potential to cause an epidemic/pandemic; (iv) similar clinical features (i.e. nonspecific febrile illness with the potential to develop into pneumonia and acute respiratory distress syndrome; difficult diagnosis based on clinical features during transmission-relevant phase, including the period prior to symptom development and during early-stage symptoms); (v) unavailability of a vaccine and/or difficulty to contain an outbreak through vaccination. As a result, we considered:

- SARS-CoV-2/COVID-19
- SARS-CoV-1/SARS
- MERS-CoV/MERS
- Influenza



For peer review only - http://bmjopen.bmj.com/site/about/guidelines.xhtml

Studies in all other populations/contexts, including evidence on infectious diseases less relevant to the current SARS-CoV-2/COVID-19 pandemic (e.g. avian influenza, Ebola, meningitis, HIV/AIDS, tuberculosis, dengue, plague, cholera, fever, smallpox, measles, or zika virus) were excluded.

Regarding the intervention, we considered travel-related control measures affecting human travel across national or sub-national borders, specifically:

- Closure of borders (borders closed to entry and/or exit)
- Travel bans (suspension of flights, ground crossing, ship itineraries, refusal of entry or travel, visa suspension/denial) between countries, and between regions and large cities within countries
- Travel restrictions (i.e., varying levels of travel reductions) between countries, and between regions and large cities within countries)
- Entry/exit screening (e.g. temperature measurement, health questionnaire, thermography, physical examination, laboratory tests, passive observation and/or follow-up quarantine) at airports, ports, land borders, and train stations
- Quarantine/isolation of travelers from affected regions (at borders, at designated institutions or at home)
- Any combination of the above measures and/or with other control measures (e.g. combination of border closure and school closure).

All other interventions were excluded. This comprised those not directly related to travel (e.g. community-based quarantine, hygiene measures, bans on mass gatherings), those related to the movement of animals or goods, travel warnings or travel advice issued by the WHO or national governments, situations affecting travel but not representing travel-related control measures (e.g. school holidays), and those solely concerned with the effectiveness of laboratory tests rather than their implementation as part of an entry/exit screening procedure.

Regarding outcomes, we considered those assessing the quantitative impacts of the interventions on:

- Infectious disease outcomes (e.g. number/proportion of cases, number/proportion of deaths, time to/delay in epidemic arrival or peak, reproduction number, healthcare demand and utilization)
- Screening outcomes (e.g. number/proportion of persons screened, number/proportion of those screened identified as cases)
- Other health outcomes (e.g. psychosocial impact)
- Economic outcomes (e.g. travel volumes, costs of measures implemented, losses to different economic sectors)
- Social outcomes (e.g. stigmatization/discrimination of foreigners, xenophobia, migration volumes)

All other outcomes, such as those on the human rights and legal implications of interventions, were excluded.

Regarding study types, we considered all types of empirical studies which quantitatively evaluate impact (e.g. epidemiologic, modeling, simulations, and econometric studies). All other study types, including qualitative studies, diagnostic studies focused on test performance and non-empirical studies (e.g. commentaries, narrative and systematic reviews), were excluded.



For peer review only - http://bmjopen.bmj.com/site/about/guidelines.xhtml

## Study selection

After de-duplication, all titles and abstracts were screened by one reviewer (shared among several team members), excluding only those studies which were clearly irrelevant. For all studies deemed potentially relevant or unclear at the title/abstract screening stage, one reviewer screened the full text. At this stage, a final decision regarding eligibility was made. We adopted a very inclusive approach: any unclear cases were discussed with a second reviewer, and remaining uncertainties were resolved with involvement of a third reviewer and/or the whole review team. In addition, all studies excluded at the full text screening stage based on the intervention (i.e. not addressing travel-related control measures) were double-checked by a second reviewer to make sure that no relevant studies were excluded.

We used Endnote to manage collection and de-duplication of records. For title and abstract screening, we used Rayyan, a web-based application designed for citation screening for systematic reviews (11). We documented reasons for the exclusion of full texts and reported those using Microsoft Excel.

For both the title/abstract and full text screening stages, we developed screening guidance forms to ensure that all reviewers screen similarly and consistently. We discussed inconsistencies and challenges encountered within the review team, after having screened approximately 300 titles/abstracts and 50 full texts, and subsequently refined the screening guidance. We additionally collected and clarified all uncertainties in screening on a rolling basis. These were discussed in daily online meetings to ensure consistency in screening across multiple reviewers and that any questions and comments were addressed.

## Data extraction

One reviewer extracted study characteristics and data into the pre-defined categories of the data extraction form in Microsoft Excel (see online supplementary file S4). The extraction form was pilot-tested in the review team. The following categories were covered by the extraction form: population, setting, and context; characterization of the respiratory pathogen/disease (i.e. SARS CoV-2/COVID-19, SARS CoV-1/SARS, MERS CoV/MERS, influenza), types of interventions (e.g. entry/exit screening, border closure, quarantine of travelers, and travel bans), comparisons (where available), outcomes of interest (e.g. health, economic, and social impact), study designs (e.g. epidemiologic study, modeling study).

## Mapping

We charted the extracted data based on categories and present findings in a tabular, narrative, or graphical manner. Most of these categories were defined a priori, while one category, namely, geographical setting was adapted post hoc. Specifically, we sought to define, summarize, and present clusters of studies based on the pathogen/disease (i.e. SARS-CoV-2/COVID-19, SARS-CoV-1/SARS, MERS-CoV/MERS, influenza), type of intervention (border closure, entry/exist screening including follow-up measures, such as isolation of positive cases, travel ban, travel-related quarantine in the absence of entry/exit screening, travel restrictions, and multiple travel-related control measures combined), timing of the intervention (i.e. early phase, local transmission phase, post-peak phase, unclear phase), outcomes of interest (i.e. infectious disease outcomes, screening outcomes, other


For peer review only - http://bmjopen.bmj.com/site/about/guidelines.xhtml

health outcomes, economic and social outcomes) and study designs (e.g. epidemiologic study, modeling study). All data presented in the tables, text and graphics were double-checked by a second reviewer with an emphasis on accuracy in reporting on populations, interventions and outcomes and in relation to consistency of presentation.

## Patient and public involvement

Considering the time constraints and the nature of this research (i.e., a rapid systematic evidence map), it was conducted without patient and public involvement.

# Results

Database searches yielded a total of 4,928 unique records. Through snowballing of reviews identified through the database searches, we identified an additional 1,700 studies. During the title/abstract screening stage, we excluded 4,445 records as clearly irrelevant. We subsequently assessed the full texts of 483 records and excluded another 361 records. Overall, we included 122 studies in this evidence map (see online supplementary file S5 for the full list). Of these, 80 were journal articles, 41 were pre-prints and 1 was a report. Figure 1 provides an overview of our searching and screening procedures.

## Characteristics of included studies

The 122 included studies were characterized by substantial heterogeneity in relation to the countries and populations targeted, the diseases addressed, the types of travel-related control measures examined (sometimes assessed against a range of other interventions to contain an epidemic/pandemic), and the numerous outcomes assessed. They comprise both studies that are inferential in nature and studies that are descriptive in nature, varying greatly in the specific methods applied; notably, many of the studies addressing COVID-19 were pre-prints. We summarize these aspects below, and provide a description of each study in Table 1.

**Countries and settings**

Included studies assessed interventions across the globe, including, according to WHO world regions, from the Western Pacific Region (WPR) (n=71), the South-East Asia region (SEAR) (n=5), the Region of the Americas (AMR) (n=12), the European region (EUR) (n=8), and the African Region (AFR) (n=2). None of the studies included in this evidence map was conducted in a country of the Eastern Mediterranean Region (EMR). The specific countries in which interventions were implemented are listed in Table 1 (under “regions protected by travel related measure”). Several modeling studies looked at hypothetical regions (n=16).

Of the identified interventions, some were implemented in island states (n=24) or in what we define quasi-island states (n=9), such as the UK or Hong Kong, which have limited land borders connected through, for example, a tunnel. Most interventions were implemented in non-island states (n=74). The remaining interventions assessed hypothetical regions, some of which were framed as comparable to island states, while others were more general in their framing and assumptions.



For peer review only - http://bmjopen.bmj.com/site/about/guidelines.xhtml

**Table 1**: Characteristics of included studies

| Study[1] | Study type | WHO Region | Geographical setting | Region protected by travel-related measure | Region restricted by travel-related measure | Disease | Intervention category | Outcome category |
|---|---|---|---|---|---|---|---|---|
| Adekunle 2020 (pre-print) | Inferential | WPR | Island state | Australia | China, Iran, South Korea, Italy | COVID-19 | Travel ban | Number or proportion of cases |
| Aleta 2020 (pre-print) | Inferential | WPR | Non-island state | Regions within China | Wuhan | COVID-19 | Multiple travel-related control measures | Number or proportion of cases |
| Anonymous | Descriptive | AMR | Non-island state | Vancouver, Toronto, other parts of Canada | All other countries | SARS | Entry/exit screening | Detection of high risk persons or cases |
| Anzai 2020 | Inferential | WPR | Non-island state | Multiple countries | China | COVID-19 | Multiple travel-related control measures | Number or proportion of cases; Temporal development of epidemic |
| Arima 2020 | Descriptive | WPR | Island state | Japan | China | COVID-19 | Entry/exit screening; Travel-related quarantine | Detection of high risk persons or cases |
| Arino 2007 | Inferential | n.a. | Hypothetical | Hypothetical | Hypothetical | Hypothetical infectious disease | Travel restriction | Number or proportion of cases |
| Bajardi 2011 | Inferential | All | Non-island state | All countries | Mexico | Influenza (H1N1 2009 pandemic) | Travel ban; Travel restriction: land | Temporal development of epidemic |
| Banholzer 2020 (pre-print) | Inferential | EUR; WPR; AMR | Non-island state | Austria, Australia, Belgium, Canada Denmark, France, Finland, Italy, | All other countries | COVID-19 | Border closure | Number or proportion of cases |

[1] The full reference list of included studies is presented in Supplementary file S5.

| Study[1] | Study type | WHO Region | Geographical setting | Region protected by travel-related measure | Region restricted by travel-related measure | Disease | Intervention category | Outcome category |
|---|---|---|---|---|---|---|---|---|
| | | | | Norway, Switzerland, Sweden, Spain, and US | | | | |
| Bolton 2012 | Inferential | WPR | Non-island state | Mongolia | Neighboring countries | Influenza (H1N1 2009 pandemic) | Travel restriction | Number or proportion of cases |
| Boyd 2018 | Inferential | WPR | Island state | New Zealand | All other countries | Hypothetical infectious disease | Border closure | Costs |
| Boyd 2017 | Inferential | WPR | Island state | New Zealand | All other countries | Hypothetical infectious disease | Border closure | Costs |
| Caley 2007 | Inferential | n.a. | Hypothetical | Hypothetical | Hypothetical | Influenza | Entry/exit screening; Travel restriction: air | Detection of high risk persons or cases; Temporal development of epidemic |
| Chang 2020 (pre-print) | Inferential | WPR | Island state | Taiwan | Different cities in Taiwan | COVID-19 | Travel restriction | Number or proportion of cases; Temporal development of epidemic |
| Cheng 2020 | Descriptive | WPR | Island state | Taiwan | China | COVID-19 | Exit/entry screening; Travel-related quarantine | Number or proportion of cases |
| Chinazzi 2020 | Inferential | WPR | Non-island state | Other regions of China, all other countries | Wuhan | COVID-19 | Multiple travel-related control measures | Number or proportion of cases |
| Chiyomaru 2020 (pre-print) | Inferential | | Non-island state | Multiple countries | Multiple countries | COVID-19 | Travel ban | Number or proportion of cases |

| Study[1] | Study type | WHO Region | Geographical setting | Region protected by travel-related measure | Region restricted by travel-related measure | Disease | Intervention category | Outcome category |
|---|---|---|---|---|---|---|---|---|
| Chong 2012 | Inferential | WPR | Quasi-island state | Hong Kong | All other countries | Influenza (H1N1 2009 pandemic) | Travel restriction: land, air, and maritime | Number or proportion of cases; Temporal development of epidemic |
| Chung 2015 | Inferential | | Non-island state | Multiple countries | Multiple countries | SARS; 2006 Avian influenza; 2009 Swine flu H1N1 | Travel restriction: air | Industry impact |
| Ciofi 2008 | Inferential | EUR | Non-island state | Italy | Italy and all other countries | Influenza | Travel restriction: air | Number or proportion of cases; Temporal development of epidemic |
| Clifford 2020 (pre-print) | Inferential | n.a. | Hypothetical | Hypothetical | All other countries | COVID-19 | Entry/exit screening | Detection of high risk persons or cases; Temporal development of epidemic |
| Colizza 2007 | Inferential | All | Non-island state | All countries | All countries | Influenza | Travel restriction: air | Number or proportion of cases; Temporal development of epidemic |
| Cooper 2005 | Inferential | All | Non-island state | All countries | All countries | Influenza | Travel restriction: air | Temporal development of epidemic |
| Costantino 2020 (pre-print) | Inferential | WPR | Island state | Australia | All other countries | COVID-19 | Travel ban | Number or proportion of cases; Number or proportion of deaths; Temporal development of epidemic |
| Cowling 2020 | Inferential | WPR | Quasi-island state | Hong Kong | China, South Korea, Iran, Italy and affected | COVID-19 | Travel restriction: air and land; Travel ban; | Reproduction number; Number or proportion of cases; Acceptability |

| Study[1] | Study type | WHO Region | Geographical setting | Region protected by travel-related measure | Region restricted by travel-related measure | Disease | Intervention category | Outcome category |
|---|---|---|---|---|---|---|---|---|
| | | | | | regions in France, Germany, Japan, and Spain, Schengen Area, Macau, and Taiwan. | | Travel-related quarantine; Border closure | |
| Dandekar 2020 (pre-print) | Inferential | WPR, EUR, AMR | Island state; Quasi-island state | China, Italy, South Korea, US | All other countries | COVID-19 | Multiple travel-related control measures | Reproduction number |
| de Vlas 2009 | Descriptive | WPR | Non-island state | Regions of China | Regions of China | SARS | Travel-related quarantine | Reproduction number |
| Ediriweera 2020 (pre-print) | Inferential | SEAR | Island state | Sri Lanka | All other countries | COVID-19 | Multiple travel-related control measures; Travel-related quarantine | Number or proportion of cases; Healthcare resources |
| Eichner 2009 | Inferential | WPR | Hypothetical | Hypothetical | All other countries | Influenza (pandemic) | Travel restriction: air | Probability of epidemic |
| Epstein 2007 | Inferential | All | Non-island state | All countries (focus on US) | All countries | Influenza | Travel restriction: air | Number or proportion of cases; Temporal development of epidemic |
| Fang 2020 (pre-print) | Inferential | WPR | Non-island state | Other regions of China | Wuhan | COVID-19 | Multiple travel-related control measures | Number or proportion of cases |
| Ferguson 2006 | Inferential | AMR | Island state; Quasi-island state | US and UK | Countries connected by air traffic | Influenza (pandemic) | Border controls; Travel restrictios | Number or proportion of cases; Temporal development of epidemic |
| Flahault 2006 | Inferential | n.a. | Quasi-island state | 52 international cities and | Cities connected via air traffic | Influenza (pandemic) | Travel restriction: air | Number or proportion of cases |

| Study[1] | Study type | WHO Region | Geographical setting | Region protected by travel-related measure | Region restricted by travel-related measure | Disease | Intervention category | Outcome category |
|---|---|---|---|---|---|---|---|---|
| | | | | surrounding regions | | | | |
| Fujita 2011 | Inferential | WPR | Island state | Regions in Japan | US, Canada, and Mexico | Influenza (H1N1 2009 pandemic) | Entry/exit screening | Detection of high risk persons or cases |
| Germann 2006 | Inferential | AMR | Non-island state | US | | Influenza (pandemic) | Travel restriction: air | Number or proportion of cases |
| Glass 2006 | Inferential | n.a. | Non-island state | Non-infected region | Non-infected region | SARS | Entry/exit screening | Probability of epidemic |
| Gostic 2020 | Inferential | All | Island state | All countries | All countries | COVID-19 | Entry/exit screening | Detection of high risk persons or cases |
| Gostic 2015 | Inferential | n.a. | Hypothetical | Hypothetical | Hypothetical | SARS; MERS; Influenza (H1N1 2009 pandemic) | Entry/exit screening | Detection of high risk persons or cases |
| Goubar 2008 | Inferential | EUR | Island state; Quasi-island state | Germany and UK | China, Hong Kong | SARS | Entry/exit screening | Number or proportion of cases |
| Gunaratnam 2014 | Descriptive | WPR | Island state | Australia | All other countries | Influenza (H1N1 2009 pandemic) | Entry/exit screening | Detection of high risk persons or cases |
| Hale 2012 | Descriptive | WPR | Island state | New Zealand | Initially US, Canada, Mexico, then all other countries | Influenza (H1N1 2009 pandemic) | Entry/exit screening | Detection of high risk persons or cases |
| Hamidouche 2020 (pre-print) | Inferential | AFR | Non-island state | Algeria | All neighboring and connected (via air) countries | COVID-19 | Border closure; Travel ban; Travel-related quarantine | Number or proportion of cases; Reproduction number |
| He 2020 (pre-print) | Inferential | WPR | Island state; Quasi-island state | China, South Korea, Italy, Iran | Italy (Lombardy, Veneto, Emilia-Romagna, | COVID-19 | Multiple travel-related control measures | Number or proportion of cases; Temporal development of |

| Study[1] | Study type | WHO Region | Geographical setting | Region protected by travel-related measure | Region restricted by travel-related measure | Disease | Intervention category | Outcome category |
|---|---|---|---|---|---|---|---|---|
| | | | | | Piedmont and Marche), and China (Zhejiang). | | | epidemic |
| Hien 2010 | Inferential | WPR | Non-island state | Vietnam | Countries with confirmed cases of 2009 H1N1 | Influenza (H1N1 2009 pandemic) | Entry/exit screening | Detection of high risk persons or cases |
| Hollingsworth 2006 | Inferential | n.a. | Hypothetical | Hypothetical | Countries connected via air traffic | SARS and Influenza | Travel restriction: air | Number or proportion of cases; Temporal development of epidemic |
| Hossain 2020 (pre-print) | Inferential | WPR | Non-island state | Other regions of China | Wuhan | COVID-19 | Multiple travel-related control measures | Temporal development of epidemic |
| Hou 2020 (pre-print) | Inferential | WPR | Non-island state | Other regions of China | Wuhan | COVID-19 | Multiple travel-related control measures | Number or proportion of cases; Reproduction number |
| Hsieh 2007 | Inferential | n.a. | Hypothetical | Hypothetical | Hypothetical | Influenza (endemic) | Travel ban | Reproduction number |
| Hsieh 2006 | Inferential | WPR | Island state | Taiwan | Countries with SARS cases | SARS | Travel-related quarantine | Number or proportion of cases |
| Jia 2020 | Inferential | WPR | Non-island state | Other regions of China | Wuhan | COVID-19 | Multiple travel-related control measures | Number or proportion of cases; Other |
| Jiang 2020 (pre-print) | Inferential | WPR | Non-island state | Regions of China | Regions of China | COVID-19 | Multiple travel-related control measures | Other |
| Kerneis 2008 | Inferential | n.a. | Non-island state | 52 international cities and surrounding regions | 52 international cities and surrounding regions | Influenza (pandemic) | Travel restriction: air | Number or proportion of cases; Temporal development of epidemic |

| Study[1] | Study type | WHO Region | Geographical setting | Region protected by travel-related measure | Region restricted by travel-related measure | Disease | Intervention category | Outcome category |
|---|---|---|---|---|---|---|---|---|
| Khan 2013 | Inferential | AMR | Non-island state | Other countries | Mexico | Influenza (H1N1 2009 pandemic) | Entry/exit screening | Detection of high risk persons or cases; Other |
| Kim 2017 | Inferential | n.a. | Hypothetical | Hypothetical | Hypothetical | Hypothetical infectious disease | Entry/exit screening | Reproduction number |
| Kong 2020 (pre-print) | Inferential | WPR | Non-island state | Other regions of China | Wuhan and other cities in Hubei | COVID-19 and influenza | Multiple travel-related control measures | Number or proportion of cases |
| Kraemer 2020 | Inferential | WPR | Non-island state | Other regions of China | Wuhan and other cities in Hubei | COVID-19 | Multiple travel-related control measures | Number or proportion of cases |
| Kuo 2009 | Descriptive | WPR | Island state | Taiwan | All affected countries (e.g., Mexico, USA, and Canada) | Influenza (H1N2 2009 pandemic) | Entry/exit screening | Detection of high risk persons or cases |
| Lai 2020 (pre-print) | Inferential | WPR | Non-island state | Other regions of China | Wuhan | COVID-19 | Multiple travel-related control measures | Number or proportion of cases |
| Lam 2011 | Inferential | WPR | Quasi-island state | Hong Kong | All other countries | Influenza (H1N1 2009 pandemic) | Travel restriction | Probability of epidemic; Temporal development of epidemic |
| Lau 2020 (pre-print) | Inferential | WPR | Non-island state | Other regions of China | Wuhan and other cities in Hubei | COVID-19 | Multiple travel-related control measures | Number or proportion of cases; Temporal development of epidemic |
| Lee 2012 | Inferential | n.a. | Hypothetical | Hypothetical | Hypothetical | Influenza (H1N1 2009 pandemic) | Travel restriction | Temporal development of epidemic |
| Li 2020 | Inferential | WPR | Non-island state | Other regions of | Wuhan and other | COVID-19 | Multiple travel- | Number or proportion |

| Study[1] | Study type | WHO Region | Geographical setting | Region protected by travel-related measure | Region restricted by travel-related measure | Disease | Intervention category | Outcome category |
|---|---|---|---|---|---|---|---|---|
| (pre-print) | | | | China | cities in Hubei | | related control measures | of cases |
| Lin 2020 (pre-print) | Inferential | WPR | Non-island state | Other regions of China | Wuhan | COVID-19 | Multiple travel-related control measures | Reproduction number; Other |
| Linka 2020 (pre-print) | Inferential | EUR | Non-island state | All European countries | All European countries | COVID-19 | Multiple travel-related control measures | Number or proportion of cases |
| Liu 2006 | Inferential | n.a. | Hypothetical | Hypothetical | Hypothetical | Hypothetical infectious disease | Entry/exit screening | Reproduction number |
| Liu 2020 | Inferential | WPR | Non-island state | Other regions of China | Wuhan | COVID-19 | Multiple travel-related control measures | Number or proportion of cases |
| Liu 2020a (pre-print) | Inferential | WPR | Non-island state | Other Regions of China | Cities outside of Hubei | COVID-19 | Multiple travel-related control measures | Number or proportion of cases |
| Malmberg 2020 (pre-print) | Inferential | n.a. | Hypothetical | Hypothetical | Hypothetical | Hypothetical infectious disease | Travel restriction; Travel-related quarantine | Probability of epidemic |
| Malone 2009 | Inferential | AMR | Non-island state | US | Asia | Influenza (pandemic) | Entry/exit screening | Detection of high risk persons or cases; Number or proportion of cases; Number of deaths |
| Mandal 2020 | Inferential | SEAR | Non-island state | India | China, Hong Kong, Singapore, Thailand, Japan, South Korea, Iran, and Italy | COVID-19 | Entry/exit screening | Temporal development of epidemic; Number or proportion of cases |

| Study[1] | Study type | WHO Region | Geographical setting | Region protected by travel-related measure | Region restricted by travel-related measure | Disease | Intervention category | Outcome category |
|---|---|---|---|---|---|---|---|---|
| Marcelino 2013 | Inferential | All | Non-island state | All countries | Mexico city | Influenza (pandemic) | Travel restriction: air | Number or proportion of cases |
| Mbuvha 2020 (pre-print) | Inferential | AFR | Non-island state | South Africa | South Africa and the all other countries | COVID-19 | Travel ban | Number or proportion of cases; Temporal development of epidemic |
| Mondal 2020 (pre-print) | Inferential | SEAR | Non-island state | India | India | COVID-19 | Travel ban | Number or proportion of cases |
| Moriatry 2020 | Inferential | WPR, AMR | Non-island state | Japan, US, home countries of cruise ship passengers | Home countries of cruise ships | COVID-19 | Travel-related quarantine | Detection of high risk persons or cases |
| Mummert 2013 | Inferential | AMR | Non-island state | US | Mexico | Influenza (H1N1 2009 pandemic) | Entry/exit screening | Number or proportion of cases |
| Muraduzzaman 2017 | Descriptive | SEAR | Non-island state | Bangladesh | Saudi Arabia | MERS | Entry/exit screening | Detection of high risk persons or cases |
| Nakara 2015 | Inferential | n.a. | Hypothetical | Hypothetical | Hypothetical | Hypothetical infectious disease | Travel restriction | Reproduction number |
| Nigmatulina 2009 | Inferential | n.a. | Hypothetical | Hypothetical | Hypothetical | Influenza | Travel restriction | Number or proportion of cases |
| Nishiura 2009 | Inferential | n.a. | Hypothetical | Hypothetical | Hypothetical | Influenza (pandemic) | Travel-related quarantine | Detection of high risk persons or cases |
| Odendaal 2020 (pre-print) | Inferential | AMR | Non-island state | US | China, and other affected countries | COVID-19 | Travel restriction | Number or proportion of cases |
| Pan 2020 | Inferential | WPR | Non-island state | Other regions of China | Wuhan and other cities in Hubei | COVID-19 | Multiple travel-related control measures | Number or proportion of cases |
| Pan 2020a (pre-print) | Inferential | WPR | Non-island state | China | China | COVID-19 | Multiple travel-related control | Number or proportion of cases; Reproduction |

| Study[1] | Study type | WHO Region | Geographical setting | Region protected by travel-related measure | Region restricted by travel-related measure | Disease | Intervention category | Outcome category |
|---|---|---|---|---|---|---|---|---|
| | | | | | | | measures | number |
| Pang 2003 | Descriptive | WPR | Non-island state | Beijing | All other countries | SARS | Entry/exit screening | Number or proportion of cases |
| Pinkas 2020 | Descriptive | EUR | Non-island state | Poland | All other countries | COVID-19 | Entry/exit screening; Border closure; Travel ban; Travel-related quarantine | Number or proportion of cases |
| Pitman 2005 | Descriptive | EUR | Quasi-island state | UK | Any of the top 100 sources of international airline passengers | SARS, Influenza | Entry/exit screening | Detection of high risk persons or cases |
| Priest 2013 | Descriptive | WPR | Island state | New Zealand | Australia | Influenza (type A and B) | Entry/exit screening | Detection of high risk persons or cases |
| Pullano 2020 | Inferential | WPR | Non-island state | Other regions of China and European countries | Wuhan | COVID-19 | Travel ban | Probability of epidemic |
| Qiu 2020 (pre-print) | Inferential | WPR | Non-island state | Other regions of China | Wuhan and other cities in Hubei | COVID-19 | Multiple travel-related control measures | Number or proportion of cases |
| Quilty 2020 (pre-print) | Inferential | WPR | Non-island state | Beijing, Chongqing, Hangzhou, and Shenzhen | Wuhan | COVID-19 | Multiple travel-related control measures | Number or proportion of cases; Probability of epidemic |
| Ray 2020 (pre-print) | Inferential | SEAR | Non-island state | India | All other countries | COVID-19 | Travel ban | Number or proportion of cases |
| Sakaguchi 2012 | Descriptive | WPR | Island state | Japan | Mexico, US, and Canada | Influenza (A H1N1 2009 pandemic) | Entry/exit screening | Detection of high risk persons or cases |
| Samaan 2004 | Descriptive | WPR | Island state | Australia | All countries | SARS | Entry/exit screening | Detection of high risk |

| Study[1] | Study type | WHO Region | Geographical setting | Region protected by travel-related measure | Region restricted by travel-related measure | Disease | Intervention category | Outcome category |
|---|---|---|---|---|---|---|---|---|
| | | | | | affected by SARS | | | persons or cases |
| Sang 2012 | Inferential | n.a. | Hypothetical | Hypothetical | Hypothetical | SARS | Entry/exit screening | Reproduction number; Temporal development of epidemic; Number or proportion of cases; Number or proportion of deaths |
| Scala 2020 (pre-print) | Inferential | EUR | Non-island state | Individual regions of Italy | Individual regions of Italy | COVID-19 | Travel restriction | Temporal development of epidemic |
| Scalia Tomba 2008 | Inferential | n.a. | Hypothetical | Hypothetical | Hypothetical | Influenza (pandemic) | Travel restriction/border control | Temporal development of epidemic |
| Shi 2020 (pre-print) | Inferential | WPR | Non-island state | Other regions of China | Wuhan | COVID-19 | Multiple travel-related control measures | Number or proportion of cases |
| Song 2020 (Wang 2020) | Inferential | WPR | Non-island state | Other regions of China | Wuhan | COVID-19 | Multiple travel-related control measures | Number or proportion of cases; Reproduction number |
| St. John 2005 | Descriptive | AMR | Non-island state | Canada | All other countries | SARS | Entry/exit screening | Detection of high risk persons or cases |
| Su 2020 (pre-print) | Inferential | WPR | Non-island state | Beijng, Shanghai, Guangzhou, and Shenzhen | Beijng, Shanghai, Guangzhou, and Shenzhen | COVID-19 | Multiple travel-related control measures | Number or proportion of cases; Temporal development of epidemic |
| Tang 2020 | Inferential | WPR | Non-island state | Beijing | Wuhan and other cities in China | COVID-19 | Multiple travel-related control measures | Number or proportion of cases |
| Tian 2020 (pre-print) | Inferential | WPR | Non-island state | Other regions of China | Wuhan and other cities in China | COVID-19 | Multiple travel-related control measures | Number or proportion of cases; Temporal development of |

| Study[1] | Study type | WHO Region | Geographical setting | Region protected by travel-related measure | Region restricted by travel-related measure | Disease | Intervention category | Outcome category |
|---|---|---|---|---|---|---|---|---|
| | | | | | | | | epidemic |
| Tsuboi 2020 | Descriptive | WPR | Island state | Japan | Diamond Princess | COVID-19 | Travel-related quarantine | Number or proportion of quarantined diagnosed |
| Wang 2015 | Inferential | n.a. | Hypothetical | Hypothetical (low-risk patches) | Hypothetical (high-risk patch) | Influenza (H1N1 2009 pandemic) | Entry/exit screening | Reproduction number |
| Wang 2020 (pre-print) | Inferential | WPR | Non-island state | China | Wuhan | COVID-19 | Multiple travel-related control measures | Number or proportion of cases; Number or proportion of deaths |
| Wang 2007 | Descriptive | WPR | Island state | Taiwan | All SARS-affected regions | SARS | Travel-related quarantine | Detection of high risk persons or cases diagnosed |
| Wang 2012 | Inferential | WPR | Non-island state | Different regions in China | Different regions in China | Influenza (pandemic) | Travel restriction | Temporal development of epidemic |
| Wells 2020 | Inferential | WPR | Non-island state | All other countries | China | COVID-19 | Multiple travel-related control measures | Number or proportion of cases; Probability of epidemic |
| Weng 2015 | Inferential | WPR | Hypothetical | Hypothetical | Hypothetical | Influenza (H1N1 2009 pandemic) | Travel restriction | Number or proportion of cases |
| Wilder-Smith 2003 | Descriptive | WPR | Non-island state | Singapore | All SARS-affected regions | SARS | Entry/exit screening | Detection of high risk persons or cases |
| Wood 2007 | Inferential | WPR | Non-island state | Melbourne and Sydney | Sydney and Darwin | Influenza | Travel restriction: air | Temporal development of epidemic |
| Yang 2020 | Inferential | WPR | Non-island state | Other regions of China, particularly Guangdong and Zhjiang provinces | Hubei | COVID-19 | Multiple travel-related control measures | Number or proportion of cases; Temporal development of epidemic |
| Ying 2020 | Inferential | WPR | Non-island state | Other regions of | Hubei | COVID-19 | Multiple travel- | Number or proportion |

| Study[1] | Study type | WHO Region | Geographical setting | Region protected by travel-related measure | Region restricted by travel-related measure | Disease | Intervention category | Outcome category |
|---|---|---|---|---|---|---|---|---|
| (pre-print) | | | | China | | | related control measures | of cases |
| Yu 2012 | Inferential | WPR | Non-island state | China | All other countries | Influenza (H1N1 2009 pandemic) | Entry/exit screening | Number or proportion of cases |
| Yuan 2020 (pre-print) | Inferential | WPR | Non-island state | Other regions of China and countries | Wuhan | COVID-19 | Multiple travel-related control measures | Temporal development of epidemic |
| Yuan 2020a (pre-print) | Inferential | WPR | Non-island state | China | Wuhan | COVID-19 | Multiple travel-related control measures | Number or proportion of cases |
| Zhang 2012 | Descriptive | WPR | Non-island state | Beijing | All other countries | Influenza (H1N1 2009 pandemic) | Entry/exit screening | Number or proportion of screened identified as cases |
| Zhang 2014 | Inferential | n.a. | Hypothetical | Hypothetical | Hypothetical | Influenza (pandemic) | Entry/exit screening | Number or proportion of cases; Temporal development of epidemic |
| Zhang 2019 | Inferential | n.a. | Hypothetical | Hypothetical | Hypothetical | Influenza (H1N1 2009 pandemic) | Entry/exit screening; Reductions in human mobility | Temporal development of epidemic |
| Zhang 2020 (pre-print) | Inferential | WPR | Non-island state | Other regions of China | Wuhan and other cities in Hubei | COVID-19 | Multiple travel-related control measures | Number or proportion of cases |
| Zhang 2020a (pre-print) | Inferential | WPR | Non-island state | Other regions of China | Wuhan and other cities in Hubei | COVID-19 | Multiple travel-related control measures | Number or proportion of cases; Reproduction number |
| Zhao 2020 | Inferential | WPR | Non-island state | Regions of China | Regions of China | COVID-19 | Multiple travel-related control | Number or proportion of cases; Reproduction |

| Study[1] | Study type | WHO Region | Geographical setting | Region protected by travel-related measure | Region restricted by travel-related measure | Disease | Intervention category | Outcome category |
|---|---|---|---|---|---|---|---|---|
| | | | | | | | measures | number; Temporal development of epidemic |
| Zhou 2020 (pre-print) | Inferential | WPR | Non-island state | Other regions of China | Wuhan and other cities in Hubei | COVID-19 | Multiple travel-related control measures | Number or proportion of cases; Number or proportion of deaths |
| Zlojutro 2018 | Inferential | AMR | Non-island state | USA | All other countries | Influenza (H1N1 2009 pandemic) | Entry/exit screening | Number or proportion of cases; Cost; Other |

For peer review only

### COVID-19 and other relevant respiratory infectious diseases

Included studies assessed the impact of interventions aiming to prevent or slow the transmission of COVID-19 and other infectious respiratory diseases. We identified a large number of studies focusing on COVID-19 (n=59); we also found studies focusing on SARS (n=11), MERS (n=1) and various strains of influenza (n=39). Other studies looked at multiple infectious respiratory diseases (n=5) or a hypothetical infectious disease with COVID-19-relevant properties (n=7).

Figure 2 illustrates how the number of publications concerned with each disease has developed over time, and explores where interventions have been implemented. As expected, there has been a rapid burst of research related to COVID-19 travel restrictions in 2020 (panel A), currently consisting mostly of non-peer-reviewed pre-prints. Research on SARS, MERS and influenza travel restrictions is more spread over time, but also clusters around specific outbreaks (e.g. SARS 2003; H1N1 influenza 2009) (panel B).

### Intervention categories and interventions

The 122 included studies assessed the impact of a wide range of travel-related control measures. We classified these according to broad intervention categories, including border closures (n=3), entry/exit screening (n=31), travel-related quarantine (n=6), travel bans, such as suspension of international flights (n=8), and travel restrictions (n=25).

For travel restrictions, a few studies described imposing restrictions in relation to the mode of travel (e.g. restricting air, land, or maritime travel); most used the term “travel restrictions” without providing any specification. However, most of the studies in this category were modeling studies, which commonly simulated “travel restrictions” as different percentage reductions in the travel volume (e.g. 50% and 90%).

We identified a relatively large number of studies assessing the impact of a bundle of different travel-related control measures (e.g. entry/exit screening and quarantining all arriving passengers) (n=49). More than half of these (n=29) assessed the impact of the lockdown of Wuhan (n=29), combining several travel-related measures. We classified all of these studies as assessing *multiple travel-related control measures* (see online supplementary file S6), as it was impossible to identify the specific measures assessed due to lack of reporting.

Figure 3 visualizes the body of evidence according to the respective disease, intervention implemented, as well as region in which an intervention was implemented. It becomes clear that the majority of the evidence assesses multiple travel-related control measures to delay or limit the progression of COVID-19. The second largest block of evidence emerges from studies focusing on various influenza strains and the impact of travel restrictions and entry/exit screening.

A critical aspect in relation to the likely impact of travel-related control measures is the timing of implementation. Figure 4 (panel A) visualizes during which phase of an epidemic or pandemic different types of interventions addressed by included studies were implemented. With respect to the timing of implementation we distinguish the following:

- Early phase: Interventions are implemented at a time when there are either no or only singular detected/notified cases (i.e. all cases are detected and quarantined). During this phase, imported cases represent the main source of infections.



For peer review only - http://bmjopen.bmj.com/site/about/guidelines.xhtml

- Local transmission phase: Interventions are implemented during more or less widespread human-to-human transmission of the disease. During this phase local transmission and imported cases represent sources of infections.
- Post-peak phase: Interventions are implemented during/after successful containment of an initial outbreak/epidemic/pandemic with the possibility of recurrence. During this phase imported cases may again represent a major source of infections.
- Unclear phase: The phase of the outbreak/epidemic/pandemic is not reported or is not directly relevant for implementation of the intervention.

Figure 4 (panel A) shows, for example, that many interventions, especially those comprising multiple measures, are implemented when local transmission of the disease has already been established. Travel restrictions are often employed in the early phase of an epidemic or pandemic while entry/exit screening measures tend to be implemented both in the early stages, as well as throughout the phases of an epidemic or pandemic.

## Outcome categories and outcomes

We considered studies assessing five broad categories of outcomes: infectious disease outcomes, screening outcomes, other health outcomes, economic outcomes, and social outcomes. Identified studies, however, largely assessed infectious disease (n=98) and/or screening outcomes (n=25). We identified no studies concerned with other health outcomes, and very few studies assessing economic (n=5) or social outcomes (n=1).

Infectious disease outcomes included several outcomes related to disease timing and transmission, including the number or proportion of cases, the number or proportion of deaths, the reproduction number, the probability of an epidemic, demand for healthcare resources, and the temporal development of the epidemic. Screening outcomes all comprised some form of measuring the number and/or proportion of high risk persons and/or cases detected. Economic outcomes covered costs and industry impact, and social outcomes examined the acceptability of travel-related control measures. Outcomes assessed in each study, according to these categories, are listed in Table 1. A comprehensive list of specific outcomes and how often these were utilized across the evidence map can be found in online supplementary file S7.

Figure 4 (panel B) illustrates which intervention categories were assessed by different outcome categories. Infectious disease outcomes such as the number or proportion of cases, the temporal development of an epidemic, and the reproduction number were well-represented across all intervention types. Conversely, screening outcomes were assessed primarily with respect to entry/exit screening. The lack of studies assessing economic and social outcomes is also clearly visible.

## Study types

We included all studies assessing the quantitative impact of travel-related control measures on infectious disease, screening, economic and social outcomes. As a consequence of this broad scope, included studies employed a range of vastly different approaches drawing from the fields of infectious disease research, epidemiology, economics, biology, and mathematics, among others. We categorized each study as being either inferential or descriptive. Most of the studies we found were inferential in nature (n=103), aiming to retrospectively calculate or prospectively forecast the impact of one or multiple travel-related control measures on outcomes. The remaining studies were



For peer review only - http://bmjopen.bmj.com/site/about/guidelines.xhtml

descriptive in nature (n=19), aiming to describe the impact of control measures through summary statistics and/or graphics.

Within these broad categories, however, included studies varied greatly with regard to the specific approach taken. Inferential studies applied numerous modeling and epidemiological techniques; compartmental models, such as SIR models (S: susceptible, I: infectious, R: recovered) or SIR model derivatives, were common. Several studies also applied spatial models to explore how disease transmission moves geographically. Epidemiological time-series models, as well as other epidemiological modeling and testing strategies, were also common. Descriptive studies comprised primarily observational studies and graphical summary studies, both of which measured and reported descriptive summary statistics related to intervention impact. The types of studies illustrated here are not exhaustive, and the methodological boundaries between the approaches employed are sometimes blurry. In fact, several studies apply multiple techniques, combining, for example, compartmental and spatial models or compartmental and time-series models. Online supplementary file S8 provides a more detailed overview of the study types included, along with examples from the included studies.

## Discussion

### Summary of findings

This evidence map provides a comprehensive overview of travel-related control measures available for the control of SARS-CoV-2/COVID-19.

Most of the included studies used infectious disease or epidemiological modeling methods to examine the impact of travel-related control measures on the current COVID-19 pandemic. The COVID-19 evidence base, however, largely consists of pre-print publications which have not yet undergone peer review and are often characterized by poor reporting and conduct (12). We also identified studies on SARS-CoV-1/SARS and influenza, mostly undertaken in the context of previous epidemics/pandemics. We found very few studies addressing MERS-CoV/MERS.

Studies were undertaken across the globe. The geographical region most represented is WPR, driven in part by a number of studies focusing on the Hubei region of China and in part by studies across the region during the previous SARS-CoV-1/SARS outbreak. AFR and EUR are the least represented; we did not identify any studies from EMR. Most studies operate at the level of countries and say little about specific settings of interest (e.g. a named airport, a specific country border) or the broader implementation context (e.g. usual border arrangements when control measures are not in place).

Travel-related control measures can be classified as (i) border closure, (ii) travel bans, (iii) travel restrictions, (iv) entry/exit screening, (v) travel-related quarantine, and (vi) multiple travel-related control measures. Studies were identified and mapped for all of these categories. We identified a relatively large body of evidence on entry/exit screening, as well as on travel restrictions. The latter also included sub-national measures, e.g. city-to-city travel restrictions across broader regions, making a clear distinction from general social distancing measures challenging. We also found many studies examining the impact of bundles of travel-related control measures. Interventions are often poorly described, both in relation to the measure itself (e.g. border closure) and in relation to how the measure is implemented or enforced (e.g. border patrols, fines, exceptions). Moreover, travel-



For peer review only - http://bmjopen.bmj.com/site/about/guidelines.xhtml

related control measures rarely happen in a vacuum – the closer to real-life the intervention (and the study), the more co-interventions tend to be involved. This makes it very difficult to assess the unique impact of a specific measure.

The impact of travel-related control measures in controlling geographical spread and overall disease transmission likely varies between early, local transmission and post peak phases of an outbreak/epidemic/pandemic, yet reporting of the timing of implementation tends to be poor. Most studies focused on the early and local transmission phases; few studies were concerned with the post-peak phase.

The identified studies almost exclusively examined infectious disease and screening outcomes. Surprisingly, we did not identify any study reporting on other health outcomes, such as implications for the physical and psychosocial health of stranded travelers, of those unable to visit family members, of regular commuters unable to reach their workplace, of individuals quarantined, of people unable to obtain medical treatment and other collateral damage (e.g. suspended immunization programs and impacts on food supply due to limited air, maritime and land travel of either people or goods). Only three included studies were concerned with economic and social outcomes.

The bulk of the evidence derives from modeling studies. In an acute outbreak situation, the time and resources that can be dedicated to conducting timely, empirical research are limited. Modeling studies vary greatly in type (e.g. SIR-like models vs. time series models), and in the underlying assumptions regarding the disease, interventions or regions/settings. Additionally, many studies do not fall into clean categories, and a single study may combine compartmental infectious disease models with epidemiological spatial models. They also vary with respect to whether and how they have been validated through real-life applications.

## Methodological limitations of this study

This evidence map was put together over ten days, and the process is thus characterized by several limitations.

While we conducted searches in three major databases and two COVID-specific databases, these were mostly health-centric. We only searched Web of Science – comprising Science Citation Index Expanded (1900-present), Social Sciences Citation Index (1900-present) and Emerging Sources Citation Index (2015-present) – to identify social and economic studies. As we identified very few economic and social outcomes, it is likely that there is another body of evidence to be located with more focused searches in specific economic and social science databases. In addition, we did not search for grey literature sources.

The unspecific and inconsistent reporting of primary studies with regard to interventions, especially when a package of control measures was investigated, sometimes made inclusion/exclusion decisions difficult. While we developed and calibrated screening guidance to ensure consistency among reviewers, it is nevertheless possible that we excluded some travel-related control measures that were not explicitly described as such. Also, we did not undertake double-screening of all studies but only re-assessed a sub-set of studies excluded at the full-text screening stage. In general,


For peer review only - http://bmjopen.bmj.com/site/about/guidelines.xhtml

however, we applied a very conservative approach to title/abstract, as well as full-text screening, where any uncertainties associated with a study were marked for further checking by a second reviewer and/or for discussion among the whole review team.

We developed and calibrated data extraction guidance to be used consistently by reviewers. Nevertheless, we had to refine some categories post hoc. We also had a large number of reviewers extracting data, which created heterogeneity in the dataset. To address this, a second reviewer double-checked all extracted information for each of the main domains, i.e. all information regarding interventions, outcomes and study design. Overall, high-quality data extraction was limited by poor reporting of the travel-related control measures and the specific contexts in which they are implemented in primary studies (13).

Finally, we did not include travel warning or travel advice in the evidence map, which limits the scope of this map. While these may be classified as travel-related control measures (13), their inclusion would have likely broadened our search strategy and prolonged the timeline of its development. In general, lack of intervention specification in the included studies makes it challenging to draw clear categories of interventions without potential overlap (e.g. drawing clear distinction between a travel ban vs. a travel restriction, when these are used only as descriptors in the studies without further specification).

## Implications for moving forward

An evidence map is not designed to assess the effectiveness of interventions, in this case the effectiveness of travel-related control measures in controlling infectious disease spread. The present evidence map sheds light on the variety of evidence available with regards to the quantifiable impacts of travel-related control measures, the outcomes utilized, as well as the study types employed. It thus represents a stepping stone towards a systematic review on the effectiveness of all or a subset of travel-related control measures.

Given our health-centric searches, we feel confident that we have identified most of the available body of evidence regarding the quantifiable impacts of travel-related control measures on health. In terms of conducting a systematic review of effectiveness it would, however, be advisable to undertake additional forward-backward citation searches and/or similar studies searches with included studies. We identified a number of challenges related to the full analysis of this evidence base. These include: (i) classifying interventions in an appropriate and consistent manner; (ii) capturing the details of interventions (e.g. components, timing, implementation characteristics) and co-interventions through an analysis of linked sources of evidence; (iii) assessing the quality and usefulness of different types of studies ranging from simple observational studies to complex modeling studies; (iv) quantitatively synthesizing this extremely heterogeneous evidence base; and (v) dealing with the large number of pre-print studies and their varying quality. Moving forward with a full analysis, we suggest reviewers plan ahead and develop strategies on how these challenges can be adequately managed.

Importantly, decisions to maintain or stop travel-related control measures are determined by a range of factors beyond effectiveness, including legal and human rights aspects, as well as considerations of broader health, economic and social implications, as well as socio-cultural and political acceptability. Gathering evidence about these broader factors in a systematic manner was beyond the scope of the current evidence map, however such aspects might be important to inform decision-making. Future



For peer review only - http://bmjopen.bmj.com/site/about/guidelines.xhtml

evidence synthesis would therefore require a different scope and a broader search strategy, notably encompassing searches in economics and social science databases, as well as multidisciplinary databases (e.g. EconLit, PsycINFO and Scopus).

Contributors: JB, AM, ER, and OH defined the study scope and developed the study protocol with significant intellectual input from all review authors. JB and AM coordinated the entire study process. IK designed the search strategy and conducted all the searches. JB, AM, RB, MC, KG, LMP, PP, KS, BS, JMS, SV and ER conducted data screening and extraction. JB, AM, JMP, LMP and PP did data mapping. JB, AM, and ER prepared the first draft of the manuscript, which was further critically examined by all the authors and revised based on their feedback. All review authors reviewed and approved the final draft.

Competing interests: The authors declare they have no competing interests.

Funding: The conduct of this evidence map was funded by the World Health Organization (WHO).

Disclaimer: This study was commissioned and paid for by the World Health Organization (WHO). Copyright in the original work on which this article is based belongs to WHO. The authors have been given permission to publish this article. The authors alone are responsible for the views expressed in this publication and they do not necessarily represent the views, decisions or policies of WHO.

Data sharing statement: No original data were generated for this study. All studies included in the evidence map are presented in the supplementary file.

Patient consent: Not applicable.

Provenance and peer review: Commissioned by the World Health Organization (WHO) and externally peer reviewed.

Acknowledgements: We are grateful to Susan L Norris and Carmen Dolea for their guidance in scoping this evidence map.

For peer review only

## References


1. WHO. Novel Coronavirus (2019-nCoV) Situation Report - 1. World Health Organization; 2020.

2. WHO. Novel Coronavirus (2019-nCoV) Situation Report - 51. World Health Organization; 2020.

3. WHO. Weekly update on COVID-19, April 8–15, 2020. Health Emergencies Programme. Geneva: World Health Organization; 2020.

4. Mateus ALP, Otete HE, Beck CR, Dolan GP, Nguyen-Van-Tam JS. Effectiveness of travel restrictions in the rapid containment of human influenza: a systematic review. Bull WHO. 2014;92(12):868-80D.

5. Ryu S, Gao H, Wong JY, Shiu EYC, Xiao J, Fong MW, et al. Nonpharmaceutical Measures for Pandemic Influenza in Nonhealthcare Settings-International Travel-Related Measures. Emerging infectious diseases. 2020;26(5):961-6.

6. WHO. Non-pharmaceutical public health measures for mitigating the risk and impact of epidemic and pandemic influenza. Licence: CC BY-NC-SA 3.0 IGO.: Licence: CC BY-NC-SA 3.0 IGO.; 2019.

7. Mouchtouri VA, Christoforidou EP, An der Heiden M, Menel Lemos C, Fanos M, Rexroth U, et al. Exit and Entry Screening Practices for Infectious Diseases among Travelers at Points of Entry: Looking for Evidence on Public Health Impact. International journal of environmental research and public health. 2019;16(23):4638-.

8. Pasquini-Descomps H, Brender N, Maradan D. Value for Money in H1N1 Influenza: A Systematic Review of the Cost-Effectiveness of Pandemic Interventions. Value Health. 2017;20(6):819-27.

9. Perez Velasco R, Praditsitthikorn N, Wichmann K, Mohara A, Kotirum S, Tantivess S, et al. Systematic review of economic evaluations of preparedness strategies and interventions against influenza pandemics. PLoS ONE. 2012;7(2):e30333.

10. Errett NA, Sauer LM, Rutkow L. An integrative review of the limited evidence on international travel bans as an emerging infectious disease disaster control measure. Journal of emergency management (Weston, Mass). 2020;18(1):7-14.

11. Ouzzani M, Hammady H, Fedorowicz Z, Elmagarmid A. Rayyan-a web and mobile app for systematic reviews. Syst Rev. 2016;5(1):210.

12. Glaszio P. Waste in covid-19 research. BMJ. 2020;369:m1847.

13. Lee K, Worsnop CZ, Grepin KA, Kamradt-Scott A. Global coordination on cross-border travel and trade measures crucial to COVID-19 response. Lancet. 2020.


For peer review only

For peer review only


For peer review only - http://bmjopen.bmj.com/site/about/guidelines.xhtml

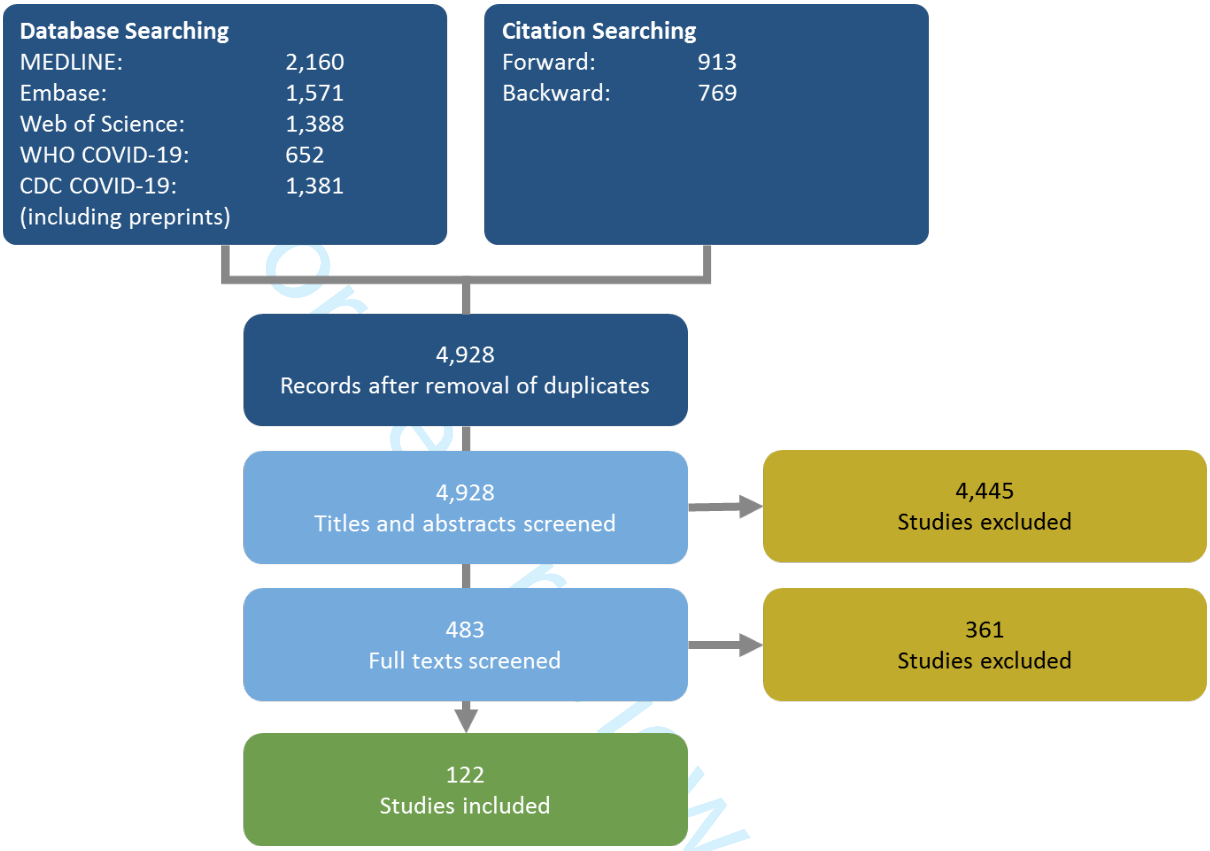


Figure 1*:* Flowchart of studies identified and finally included during different stages of searching and screening

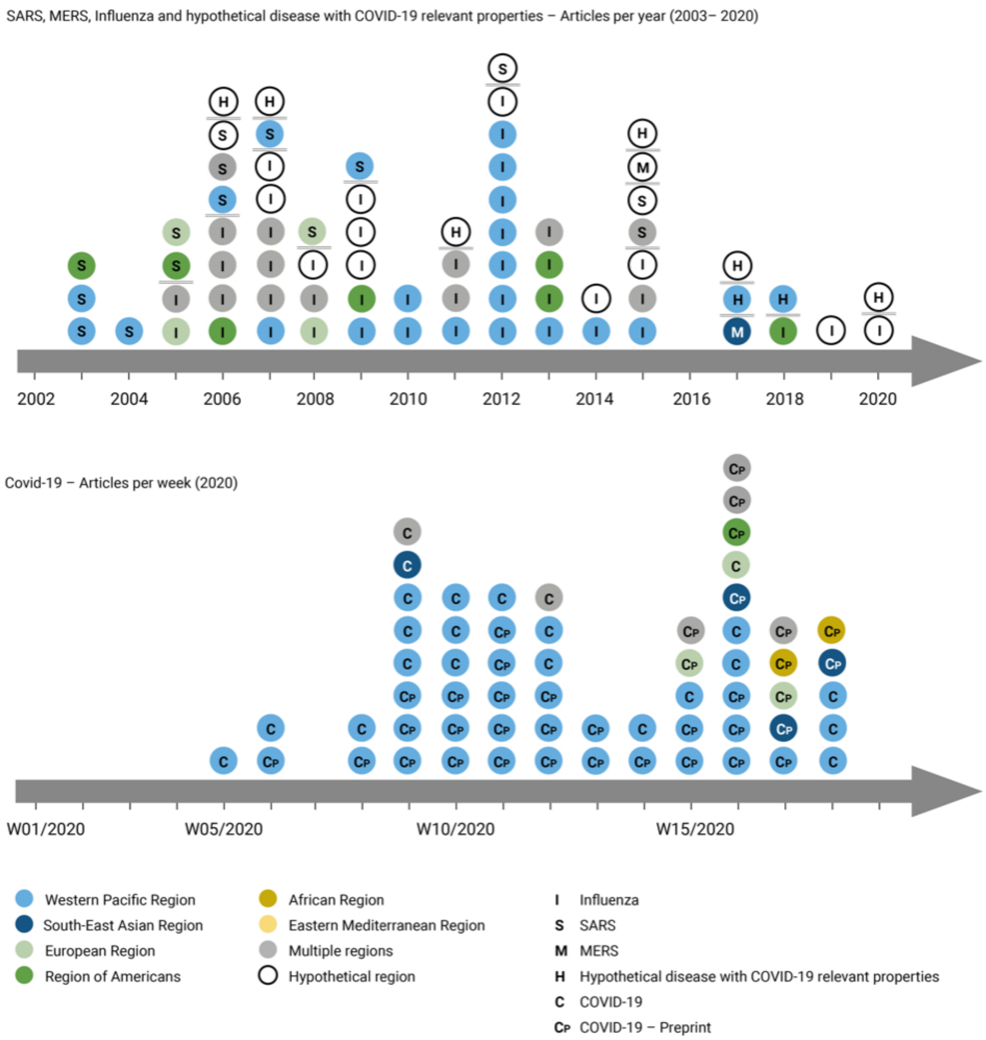


Figure 2: Timeline illustrating the publication of included studies addressing A) COVID-19, by WHO region (weeks 01/2020 to 19/2020) and B) influenza, SARS, MERS and hypothetical diseases with COVID-19 relevant properties, by WHO region (years 2002 to 2020)

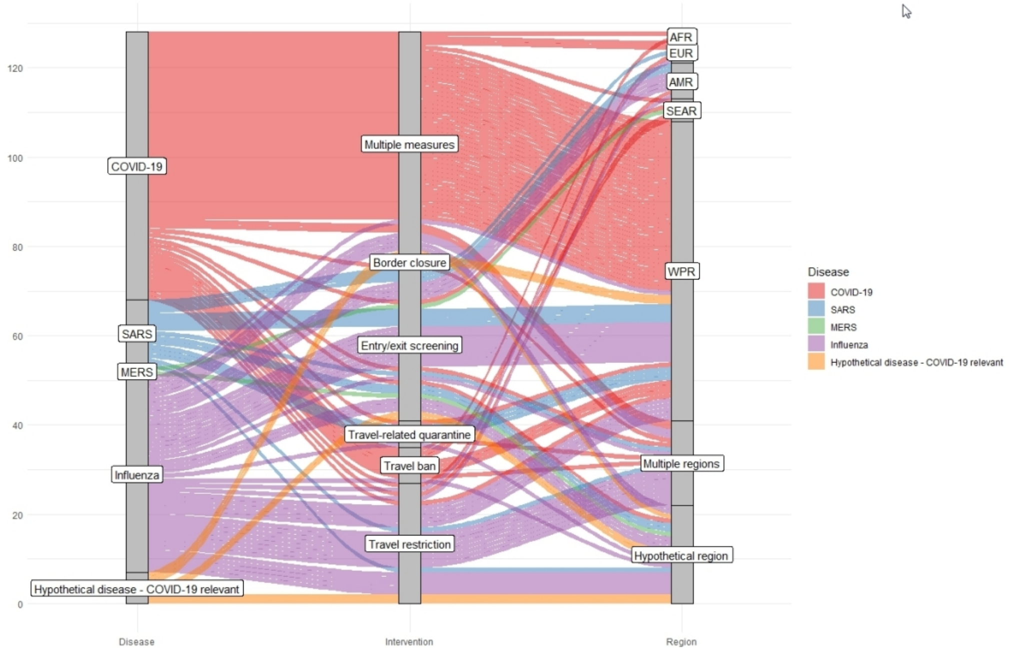


Figure 3: Visualization of the evidence base with respect to the disease, intervention category and region.

111x71mm (300 x 300 DPI)

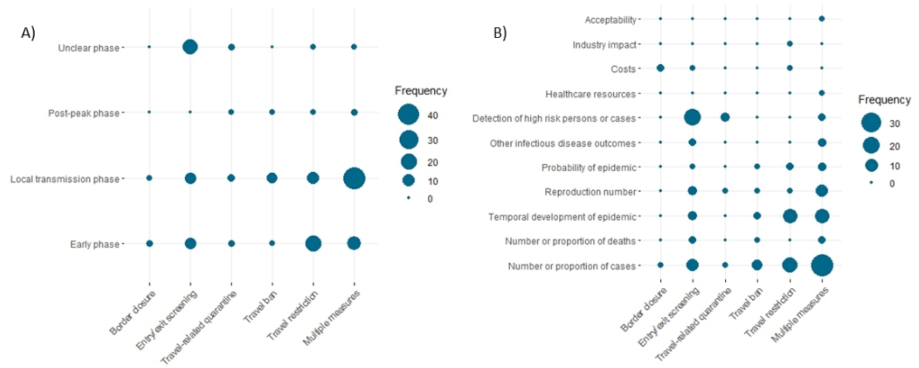


Figure 4: Bubble plots illustrating the evidence base with respect to the intervention categories and A) the time point of implementation during an epidemic or pandemic and B) the associated outcome measures.

## Supplementary file

### S1: Preferred Reporting Items for Systematic reviews and Meta-Analyses extension for Scoping Reviews (PRISMA-ScR) Checklist

| SECTION | ITEM | PRISMA-ScR CHECKLIST ITEM | REPORTED ON PAGE # |
|---|---|---|---|
| **TITLE** | | | |
| Title | 1 | Identify the report as a scoping review. | |
| **ABSTRACT** | | | |
| Structured summary | 2 | Provide a structured summary that includes (as applicable): background, objectives, eligibility criteria, sources of evidence, charting methods, results, and conclusions that relate to the review questions and objectives. | |
| **INTRODUCTION** | | | |
| Rationale | 3 | Describe the rationale for the review in the context of what is already known. Explain why the review questions/objectives lend themselves to a scoping review approach. | |
| Objectives | 4 | Provide an explicit statement of the questions and objectives being addressed with reference to their key elements (e.g., population or participants, concepts, and context) or other relevant key elements used to conceptualize the review questions and/or objectives. | |
| **METHODS** | | | |
| Protocol and registration | 5 | Indicate whether a review protocol exists; state if and where it can be accessed (e.g., a Web address); and if available, provide registration information, including the registration number. | |
| Eligibility criteria | 6 | Specify characteristics of the sources of evidence used as eligibility criteria (e.g., years considered, language, and publication status), and provide a rationale. | |
| Information sources* | 7 | Describe all information sources in the search (e.g., databases with dates of coverage and contact with authors to identify additional sources), as well as the date the most recent search was executed. | |
| Search | 8 | Present the full electronic search strategy for at least 1 database, including any limits used, such that it could be repeated. | |
| Selection of sources of evidence† | 9 | State the process for selecting sources of evidence (i.e., screening and eligibility) included in the scoping review. | |
| Data charting process‡ | 10 | Describe the methods of charting data from the included sources of evidence (e.g., calibrated forms or forms that have been tested by the team before their use, and whether data charting was done independently or in duplicate) and any processes for obtaining and confirming data from investigators. | |
| Data items | 11 | List and define all variables for which data were sought and any assumptions and simplifications made. | |
| Critical appraisal of individual sources of evidence§ | 12 | If done, provide a rationale for conducting a critical appraisal of included sources of evidence; describe the methods used and how this information was used in any data synthesis (if appropriate). | |
| Synthesis of results | 13 | Describe the methods of handling and summarizing the data that were charted. | |

For peer review only

| SECTION | ITEM | PRISMA-ScR CHECKLIST ITEM | REPORTED ON PAGE # |
|---|---|---|---|
| **RESULTS** | | | |
| Selection of sources of evidence | 14 | Give numbers of sources of evidence screened, assessed for eligibility, and included in the review, with reasons for exclusions at each stage, ideally using a flow diagram. | |
| Characteristics of sources of evidence | 15 | For each source of evidence, present characteristics for which data were charted and provide the citations. | |
| Critical appraisal within sources of evidence | 16 | If done, present data on critical appraisal of included sources of evidence (see item 12). | |
| Results of individual sources of evidence | 17 | For each included source of evidence, present the relevant data that were charted that relate to the review questions and objectives. | |
| Synthesis of results | 18 | Summarize and/or present the charting results as they relate to the review questions and objectives. | |
| **DISCUSSION** | | | |
| Summary of evidence | 19 | Summarize the main results (including an overview of concepts, themes, and types of evidence available), link to the review questions and objectives, and consider the relevance to key groups. | |
| Limitations | 20 | Discuss the limitations of the scoping review process. | |
| Conclusions | 21 | Provide a general interpretation of the results with respect to the review questions and objectives, as well as potential implications and/or next steps. | |
| **FUNDING** | | | |
| Funding | 22 | Describe sources of funding for the included sources of evidence, as well as sources of funding for the scoping review. Describe the role of the funders of the scoping review. | |

JBI = Joanna Briggs Institute; PRISMA-ScR = Preferred Reporting Items for Systematic reviews and Meta-Analyses extension for Scoping Reviews.

* Where *sources of evidence* (see second footnote) are compiled from, such as bibliographic databases, social media platforms, and Web sites.

† A more inclusive/heterogeneous term used to account for the different types of evidence or data sources (e.g., quantitative and/or qualitative research, expert opinion, and policy documents) that may be eligible in a scoping review as opposed to only studies. This is not to be confused with *information sources* (see first footnote).

‡ The frameworks by Arksey and O'Malley (6) and Levac and colleagues (7) and the JBI guidance (4, 5) refer to the process of data extraction in a scoping review as data charting.

§ The process of systematically examining research evidence to assess its validity, results, and relevance before using it to inform a decision. This term is used for items 12 and 19 instead of "risk of bias" (which is more applicable to systematic reviews of interventions) to include and acknowledge the various sources of evidence that may be used in a scoping review (e.g., quantitative and/or qualitative research, expert opinion, and policy document).

*From:* Tricco AC, Lillie E, Zarin W, O'Brien KK, Colquhoun H, Levac D, et al. PRISMA Extension for Scoping Reviews (PRISMAScR): Checklist and Explanation. Ann Intern Med. 2018;169:467–473. doi: 10.7326/M18-0850.


For peer review only - http://bmjopen.bmj.com/site/about/guidelines.xhtml

**S2:** MEDLINE search strategy and results

| Ovid MEDLINE(R) ALL 1946 to May 01, 2020 | | |
|---|---|---|
| **#** | **Searches** | **Results** |
| 1 | exp Coronavirus/ | 12994 |
| 2 | exp Coronavirus Infections/ | 11480 |
| 3 | (coronavir* or coronovir*).ti,ab,kf. | 14864 |
| 4 | ((corona* or corono*) adj1 (virus* or viral* or virinae*)).ti,ab,kf. | 578 |
| 5 | (ncov or n-cov or 2019nCoV or nCoV2019 or CO?VID-19 or CO?VID19 or WN-CoV or WNCoV or HCoV-19 or HCoV19 or 2019 novel* or SARS-CoV-2 or SARSCoV-2 or SARSCoV2 or SARS-CoV2 or SARSCov19 or SARS-Cov19 or SARSCov-19 or SARS-Cov-19 or Ncovor or Ncorona* or Ncorono* or NcovWuhan* or NcovHubei* or NcovChina* or NcovChinese* or SARS2 or SARS-2 or SARScoron?virus2 or SARS-coron?virus-2 or SARScoron?virus 2 or SARS coron?virus2).ti,ab,kf. | 7647 |
| 6 | (Middle East Respiratory Syndrome or MERS or Severe Acute Respiratory Syndrome or SARS).ti,ab,kf. | 15761 |
| 7 | Influenza, Human/ | 48540 |
| 8 | exp influenzavirus a/ or exp influenzavirus b/ or exp influenzavirus c/ | 44601 |
| 9 | (influenza* not (h?em?phil* influenza* or "h influenza*")).ti,ab,kf. | 95326 |
| 10 | (flu or H1N1 or H2N2 or H3N2 or H1N12 or H5N1).ti,ab,kf. | 36447 |
| 11 | or/1-10 | 140838 |
| 12 | travel/ or air travel/ | 25098 |
| 13 | quarantine/ | 2164 |
| 14 | (travel* or border?).ti. | 25986 |
| 15 | (travel* adj3 (restrict* or reduc* or control* or limit* or lockdown? or ban*)).ab,kf. | 1461 |
| 16 | (travel adj4 (measure? or intervention? or NPI?)).ab,kf. | 401 |
| 17 | (border? adj3 (clos* or restrict* or control* or measure?)).ab,kf. | 1014 |
| 18 | ((screen* or surveil*) adj4 (traveller? or entry or exit or border? or airport?)).ti,ab,kf. | 1124 |
| 19 | visa?.ti,ab,kf. | 2044 |
| 20 | quarantin*.ti,ab,kf. | 4807 |
| 21 | (isolat* adj6 (exposed or suspected or travel* or airport? or border?)).ti,ab,kf. | 7659 |
| 22 | or/12-21 | 59213 |
| 23 | 11 and 22 | 2405 |
| 24 | limit 23 to "humans only (removes records about animals)" | 2247 |

| | | |
|---|---|---|
| 25 | 24 and (armenian or english or french or german or italian or russian).lg. | 2160 |

For peer review only

**S3:** List of (systematic) reviews used for forward and backward citation tracking


1. Ahmad A, Krumkamp R, Reintjes R. Controlling SARS: a review on China's response compared with other SARS-affected countries. Tropical Medicine & International Health. 2009;14(s1):36-45.

2. Aledort JE, Lurie N, Wasserman J, Bozzette SA. Non-pharmaceutical public health interventions for pandemic influenza: an evaluation of the evidence base. BMC Public Health. 2007;7(1):208-.

3. Bitar D, Goubar A, Desenclos JC. International travels and fever screening during epidemics: a literature review on the effectiveness and potential use of non-contact infrared thermometers. Eurosurveillance. 2009;14(6):19115.

4. Bradt DA, Drummond CM. Avian influenza pandemic threat and health systems response. Emergency Medicine Australasia. 2006;18(5-6):430-43.

5. Cowling BJ, Lau LLH, Wu P, Wong HWC, Fang VJ, Riley S, et al. Entry screening to delay local transmission of 2009 pandemic influenza A (H1N1). BMC Infectious Diseases. 2010;10(1):82-.

6. Errett NA, Sauer LM, Rutkow L. An integrative review of the limited evidence on nternational travel bans as an emerging infectious disease disaster control measure. Journal of emergency management (Weston, Mass). 2020;18(1):7-14.

7. Gössling S, Scott D, Hall CM. Pandemics, tourism and global change: a rapid assessment of COVID-19. Journal of Sustainable Tourism. 2020:1-20.

8. Group WHOW, Bell D, Nicoll A, Fukuda K, Horby P, Monto A, et al. Non-pharmaceutical interventions for pandemic influenza, national and community measures. Emerging infectious diseases. 2006;12(1):88-94.

9. Huizer YL, Swaan CM, Leitmeyer KC, Timen A. Usefulness and applicability of infectious disease control measures in air travel: A review. Travel Medicine and Infectious Disease. 2015;13(1):19-30.

10. Inglesby TV, Nuzzo JB, O'Toole T, Henderson DA. Disease Mitigation Measures in the Control of Pandemic Influenza. Biosecurity and Bioterrorism: Biodefense Strategy, Practice, and Science. 2006;4(4):366-75.

11. Jefferson T, Del Mar CB, Dooley L, Ferroni E, Al-Ansary LA, Bawazeer GA, et al. Physical interventions to interrupt or reduce the spread of respiratory viruses. Cochrane Database of Systematic Reviews. 2011(7).

12. Juneau C-E, Pueyo T, Bell M, Gee G, Potvin L. Evidence-based, cost-effective interventions to suppress the COVID-19 pandemic: a rapid systematic review. medRxiv. 2020:2020.04.20.20054726-2020.04.20.




For peer review only - http://bmjopen.bmj.com/site/about/guidelines.xhtml

13. Kelly HA, Priest PC, Mercer GN, Dowse GK. We should not be complacent about our population-based public health response to the first influenza pandemic of the 21st century. BMC public health. 2011;11:78-.

14. Lee VJ, Lye DC, Wilder-Smith A. Combination strategies for pandemic influenza response - a systematic review of mathematical modeling studies. BMC Medicine. 2009;7(1):76-.

15. Lugnér AK, Postma MJ. Mitigation of pandemic influenza: review of cost–effectiveness studies. Expert Review of Pharmacoeconomics & Outcomes Research. 2009;9(6):547-58.

16. Mateus ALP, Otete HE, Beck CR, Dolan GP, Nguyen-Van-Tam JS. Effectiveness of travel restrictions in the rapid containment of human influenza: a systematic review. Bulletin of the World Health Organization. 2014;92(12):868-80D.

17. Mouchtouri VA, Christoforidou EP, An der Heiden M, Menel Lemos C, Fanos M, Rexroth U, et al. Exit and Entry Screening Practices for Infectious Diseases among Travelers at Points of Entry: Looking for Evidence on Public Health Impact. International journal of environmental research and public health. 2019;16(23):4638-.

18. Nicola M, Alsafi Z, Sohrabi C, Kerwan A, Al-Jabir A, Iosifidis C, et al. The Socio-Economic Implications of the Coronavirus and COVID-19 Pandemic: A Review. International journal of surgery (London, England). 2020:S1743-9191(20)30316-2.

19. Nussbaumer-Streit B, Mayr V, Dobrescu AI, Chapman A, Persad E, Klerings I, et al. Quarantine alone or in combination with other public health measures to control COVID-19: a rapid review. Cochrane Database of Systematic Reviews. 2020(4).

20. Paudel S, Dangal G, Chalise A, Bhandari TR, Dangal O. The Coronavirus Pandemic: What Does the Evidence Show? Journal of Nepal Health Research Council. 2020;18(1):1-9.

21. Rashid H, Ridda I, King C, Begun M, Tekin H, Wood JG, et al. Evidence compendium and advice on social distancing and other related measures for response to an influenza pandemic. Paediatric Respiratory Reviews. 2015;16(2):119-26.

22. Röhr S, Müller F, Jung F, Apfelbacher C, Seidler A, Riedel-Heller SG. [Psychosocial Impact of Quarantine Measures During Serious Coronavirus Outbreaks: A Rapid Review]. Psychiatrische Praxis. 2020;47(4):179-89.

23. Ryu S, Gao H, Wong JY, Shiu EYC, Xiao J, Fong MW, et al. Nonpharmaceutical Measures for Pandemic Influenza in Nonhealthcare Settings-International Travel- Related Measures. Emerging infectious diseases. 2020;26(5):961-6.

24. Selvey LA, Antão C, Hall R. Entry screening for infectious diseases in humans. Emerging infectious diseases. 2015;21(2):197-201.

25. Taghrir MH, Akbarialiabad H, Ahmadi Marzaleh M. Efficacy of Mass Quarantine as Leverage of Health System Governance During COVID-19 Outbreak: A Mini Policy Review. Archives of Iranian medicine. 2020;23(4):265-7.


For peer review only - http://bmjopen.bmj.com/site/about/guidelines.xhtml

26. Vaidya R, Herten-Crabb A, Spencer J, Moon S, Lillywhite L. Travel restrictions and infectious disease outbreaks. Journal of Travel Medicine. 2020.

27. Walters CE, Meslé MMI, Hall IM. Modeling the global spread of diseases: A review of current practice and capability. Epidemics. 2018;25:1-8.

28. Wilder-Smith A. The severe acute respiratory syndrome: impact on travel and tourism. Travel medicine and infectious disease. 2006;4(2):53-60.

29. Wilder-Smith A, Paton NI, Goh KT. Experience of severe acute respiratory syndrome in singapore: importation of cases, and defense strategies at the airport. Journal of travel medicine. 2003;10(5):259-62.

30. WHO. Handbook for public health capacity-building at ground crossings and cross-border collaboration. World Health Organization; 2020.

For peer review only



For peer review only - http://bmjopen.bmj.com/site/about/guidelines.xhtml

**S4:** Data extraction form

Study information:

- Study ID
- Study title
- Publication year
- Study source (journal, report, pre-print publication)
- For pre-print publication only: date of publication

Study design:

- Study type (e.g. modeling study, cross-sectional study, econometric study)
- Verbal summary of study (e.g., stochastic discrete event simulation model)
- Comments

Population/context:

- Country in which measure is implemented
- Study setting (e.g., Auckland international airport)
- Region protected by travel-related measure
- Region restricted by travel-related measure
- Short description of population studied (e.g., international travelers including flight passenger and crew members arriving between 27 April and 22 June 2009)
- Interconnectedness of the region
- Comments

Characteristics of pathogen/disease:

- Pathogen/disease (e.g., COVID-19, influenza)
- For influenza only: subtype (e.g. H1N1), year, endemic vs. pandemic
- Real vs. hypothetical disease outbreak
- Comments

Intervention:

- Broad measure category (e.g., entry screening)
- Verbal summary of specific travel-related measure(s) (e.g., in-flight questionnaire/symptoms)
- Real vs. hypothetical intervention
- Implementation of travel-related measure
- Phase of disease/epidemic spread (e.g. early-stage vs. late-stage)
- Co-interventions (assessed in the study)
- Co-interventions (not assessed in the study)
- Comments

Primary outcomes (repeated for each primary outcome):

- Outcome category (i.e. infectious-disease related health outcome, other health outcome, economic outcome, social outcome)
- Description of outcome
- Outcome attributable to travel-related measures (yes/no)
- Length of follow-up



For peer review only - http://bmjopen.bmj.com/site/about/guidelines.xhtml

- Comments

Secondary outcomes

- Description of any secondary (non-quantitative) outcomes

For peer review only



For peer review only - http://bmjopen.bmj.com/site/about/guidelines.xhtml

**S5:** Studies included in the evidence map

1. Adekunle AI, Meehan M, Rojaz Alvarez D, Trauer J, McBryde E. Delaying the COVID-19 epidemic in Australia: Evaluating the effectiveness of international travel bans. medRxiv. 2020:2020.03.22.20041244.

2. Aleta A, Hu Q, Ye J, Ji P, Moreno Y. A data-driven assessment of early travel restrictions related to the spreading of the novel COVID-19 within mainland China. medRxiv. 2020:2020.03.05.20031740.

3. Anonymous. Thermal image scanners to detect fever in airline passengers, Vancouver and Toronto, 2003. Canada Communicable Disease Report.30(19):165-7.

4. Anzai A, Kobayashi T, Linton NM, Kinoshita R, Hayashi K, Suzuki A, et al. Assessing the Impact of Reduced Travel on Exportation Dynamics of Novel Coronavirus Infection (COVID-19). Journal of Clinical Medicine. 2020;9(2):24.

5. Arima Y, Shimada T, Suzuki M, Suzuki T, Kobayashi Y, Tsuchihashi Y, et al. Severe Acute Respiratory Syndrome Coronavirus 2 Infection among Returnees to Japan from Wuhan, China, 2020. Emerging Infectious Diseases. 2020;26(7):10.

6. Arino J, Jordan R, van den Driessche P. Quarantine in a multi-species epidemic model with spatial dynamics. Mathematical Biosciences. 2007;206(1):46-60.

7. Bajardi P, Poletto C, Ramasco JJ, Tizzoni M, Colizza V, Vespignani A. Human mobility networks, travel restrictions, and the global spread of 2009 H1N1 pandemic. PLoS ONE. 2011;6(1).

8. Banholzer N, van Weenen E, Kratzwald B, Seeliger A, Tschernutter D, Bottrighi P, et al. Estimating the impact of non-pharmaceutical interventions on documented infections with COVID-19: A cross-country analysis. medRxiv. 2020:2020.04.16.20062141.

9. Bolton KJ, McCaw JM, Moss R, Morris RS, Wang S, Burma A, et al. Likely effectiveness of pharmaceutical and non-pharmaceutical interventions for mitigating influenza virus transmission in Mongolia. Bull WHO. 2012;90(4):264-71.

10. Boyd M, Baker MG, Mansoor OD, Kvizhinadze G, Wilson N. Protecting an island nation from extreme pandemic threats: Proof-of-concept around border closure as an intervention. PLoS ONE [Electronic Resource]. 2017;12(6):e0178732.

11. Boyd M, Mansoor OD, Baker MG, Wilson N. Economic evaluation of border closure for a generic severe pandemic threat using New Zealand Treasury methods. Australian & New Zealand Journal of Public Health. 2018;42(5):444-6.

12. Caley P, Becker NG, Philip DJ. The waiting time for inter-country spread of pandemic influenza. PLoS ONE. 2007;2(1).

13. Chang M-C, Kahn R, Li Y-A, Lee C-S, Buckee CO, Chang H-H. Modeling the impact of human mobility and travel restrictions on the potential spread of SARS-CoV-2 in Taiwan. medRxiv. 2020:2020.04.07.20053439.

14. Cheng HY, Li SY, Yang CH. Initial rapid and proactive response for the COVID-19 outbreak - Taiwan's experience. Journal of the Formosan Medical Association. 2020;119(4):771-3.



For peer review only - http://bmjopen.bmj.com/site/about/guidelines.xhtml

15. Chinazzi M, Davis JT, Ajelli M, Gioannini C, Litvinova M, Merler S, et al. The effect of travel restrictions on the spread of the 2019 novel coronavirus (COVID-19) outbreak. Science. 2020;368(6489):395-400.

16. Chiyomaru K, Takemoto K. Global COVID-19 transmission rate is influenced by precipitation seasonality and the speed of climate temperature warming. medRxiv. 2020:2020.04.10.20060459.

17. Chong KC, Ying Zee BC. Modeling the impact of air, sea, and land travel restrictions supplemented by other interventions on the emergence of a new influenza pandemic virus. BMC Infect Dis. 2012;12.

18. Chung LH. Impact of pandemic control over airport economics: Reconciling public health with airport business through a streamlined approach in pandemic control. J Air Transp Manage. 2015;44-45:42-53.

19. Ciofi degli Atti ML, Merler S, Rizzo C, Ajelli M, Massari M, Manfredi P, et al. Mitigation measures for pandemic influenza in Italy: An individual based model considering different scenarios. PLoS ONE. 2008;3(3).

20. Clifford SJ, Pearson CAB, Klepac P, Z V, voort K, Quilty BJ, et al. Interventions targeting air travellers early in the pandemic may delay local outbreaks of SARS-CoV-2. medRxiv. 2020:2020.02.12.20022426.

21. Colizza V, Barrat A, Barthelemy M, Valleron AJ, Vespignani A. Modeling the worldwide spread of pandemic influenza: Baseline case and containment interventions. PLoS Med. 2007;4(1):0095-110.

22. Cooper BS, Pitman RJ, Edmunds WJ, Gay NJ. Delaying the international spread of pandemic influenza. PLoS Med. 2006;3(6):0845-55.

23. Costantino V, Heslop DJ, MacIntyre C, Raina i. The effectiveness of full and partial travel bans against COVID-19 spread in Australia for travellers from China. medRxiv. 2020:2020.03.09.20032045.

24. Cowling BJ, Ali ST, Ng TWY, Tsang TK, Li JCM, Fong MW, et al. Impact assessment of non-pharmaceutical interventions against coronavirus disease 2019 and influenza in Hong Kong: an observational study. The lancet Public Health. 2020;17:17.

25. Dandekar R, Barbastathis G. Quantifying the effect of quarantine control in Covid-19 infectious spread using machine learning. medRxiv. 2020:2020.04.03.20052084.

26. De Vlas SJ, Feng D, Cooper BS, Fang LQ, Cao WC, Richardus JH. The impact of public health control measures during the SARS epidemic in mainland China. Trop Med Int Health. 2009;14(SUPPL. 1):101-4.

27. Ediriweera DS, de Silva NR, Malavige NG, de Silva HJ. An epidemiological model to aid decision-making for COVID-19 control on Sri Lanka. medRxiv. 2020:2020.04.11.20061481.

28. Eichner M, Schwehm M, Wilson N, Baker MG. Small islands and pandemic influenza: Potential benefits and limitations of travel volume reduction as a border control measure. BMC Infect Dis. 2009;9:160.

For peer review only

29. Epstein JM, Goedecke DM, Yu F, Morris RJ, Wagener DK, Bobashev GV. Controlling pandemic flu: The value of international air travel restrictions. PLoS ONE. 2007;2(5).

30. Fang H, Wang L, Yang Y. Human Mobility Restrictions and the Spread of the Novel Coronavirus (2019-nCoV) in China. medRxiv. 2020:2020.03.24.20042424.

31. Ferguson NM, Cummings DAT, Fraser C, Cajka JC, Cooley PC, Burke DS. Strategies for mitigating an influenza pandemic. Nature. 2006;442(7101):448-52.

32. Flahault A, Vergu E, Coudeville L, Grais RF. Strategies for containing a global influenza pandemic. Vaccine. 2006;24(44-46):6751-5.

33. Fujita M, Sato H, Kaku K, Tokuno S, Kanatani Y, Suzuki S, et al. Airport quarantine inspection, follow-up observation, and the prevention of pandemic influenza. Aviat Space Environ Med. 2011;82(8):782-9.

34. Germann TC, Kadau K, Longini Jr IM, Macken CA. Mitigation strategies for pandemic influenza in the United States. Proc Natl Acad Sci U S A. 2006;103(15):5935-40.

35. Glass K, Becker NG. Evaluation of measures to reduce international spread of SARS. Epidemiol Infect. 2006;134(5):1092-101.

36. Gostic KM, Gomez ACR, Mummah RO, Kucharski AJ, Lloyd-Smith JO. Estimated effectiveness of symptom and risk screening to prevent the spread of COVID-19. eLife. 2020;9.

37. Gostic KM, Kucharski AJ, Lloyd-Smith JO. Effectiveness of traveller screening for emerging pathogens is shaped by epidemiology and natural history of infection. eLife. 2015;2015(4).

38. Goubar A, Bitar D, Cao WC, Feng D, Fang LQ, Desenclos JC. An approach to estimate the number of SARS cases imported by international air travel. Epidemiol Infect. 2009;137(7):1019-31.

39. Gunaratnam PJ, Tobin S, Seale H, Marich A, McAnulty J. Airport arrivals screening during pandemic (H1N1) 2009 influenza in New South Wales, Australia. Med J Aust. 2014;200(5):290-2.

40. Hale MJ, Hoskins RS, Baker MG. Screening for influenza A(H1N1)pdm09, Auckland International Airport, New Zealand. Emerging Infectious Diseases. 2012;18(5):866-8.

41. Hamidouche M. COVID-19 Epidemic in Algeria: Assessment of the implemented preventive strategy. medRxiv. 2020:2020.04.21.20074443.

42. He J, Chen G, Jiang Y, Jin R, He M, Shortridge A, et al. Comparative Analysis of COVID-19 Transmission Patterns in Three Chinese Regions vs. South Korea,Italy and Iran. medRxiv. 2020:2020.04.09.20053223.

43. Hien TT, Boni MF, Bryant JE, Ngan TT, Wolbers M, Nguyen TD, et al. Early pandemic influenza (2009 H1N1) in Ho Chi Minh City, Vietnam: a clinical virological and epidemiological analysis. PLoS Medicine / Public Library of Science.7(5):e1000277.

44. Hollingsworth TD, Ferguson NM, Anderson RM. Will travel restrictions control the international spread of pandemic influenza? [3]. Nat Med. 2006;12(5):497-9.


For peer review only - http://bmjopen.bmj.com/site/about/guidelines.xhtml

45. Hossain MP, Junus A, Zhu X, Jia P, Wen T-H, Pfeiffer D, et al. The effects of border control and quarantine measures on global spread of COVID-19. medRxiv. 2020:2020.03.13.20035261.

46. Hou J, Hong J, Ji B, Dong B, Chen Y, Ward MP, et al. Changing transmission dynamics of COVID-19 in China: a nationwide population-based piecewise mathematical modeling study. medRxiv. 2020:2020.03.27.20045757.

47. Hsieh YH, King CC, Chen CW, Ho MS, Hsu SB, Wu YC. Impact of quarantine on the 2003 SARS outbreak: a retrospective modeling study. Journal of Theoretical Biology. 2006;244(4):729-36.

48. Hsieh YH, Van Den Driessche P, Wang L. Impact of travel between patches for spatial spread of disease. Bulletin of Mathematical Biology. 2007;69(4):1355-75.

49. Jia JS, Lu X, Yuan Y, Xu G, Jia J, Christakis NA. Population flow drives spatio-temporal distribution of COVID-19 in China. Nature. 2020.

50. Jiang X, eng, Chang L, Shi Y. How does the outbreak of 2019-nCoV spread in mainland China? A retrospective analysis of the dynamic transmission routes. medRxiv. 2020:2020.03.01.20029645.

51. Kerneis S, Grais RF, Boelle PY, Flahault A, Vergu E. Does the effectiveness of control measures depend on the influenza pandemic profile? PLoS ONE [Electronic Resource]. 2008;3(1):e1478.

52. Khan K, Eckhardt R, Brownstein JS, Naqvi R, Hu W, Kossowsky D, et al. Entry and exit screening of airline travellers during the A(H1N1) 2009 pandemic: A retrospective evaluation. Bull WHO. 2013;91(5):368-76.

53. Kim S, Chang DE, editors. Border screening vs. community level disease control for infectious diseases: Timing and effectiveness2017: American Institute of Physics Inc.

54. Kong XS, Liu F, Wang HB, Yang RF, Chen DB, Wang XX, et al. Epidemic prevention and control measures in China significantly curbed the epidemic of COVID-19 and influenza. medRxiv. 2020:2020.04.09.20058859.

55. Kraemer MUG, Yang CH, Gutierrez B, Wu CH, Klein B, Pigott DM, et al. The effect of human mobility and control measures on the COVID-19 epidemic in China. Science. 2020;25:25.

56. Kuo JS, Lee YH, Heish JW, Lin MC, Yang SY. Initial evaluation on screening of novel influenza A (H1N1) at international ports in Taiwan. Taiwan Epidemiol Bull. 2009;25(9):254-67.

57. Lai S, Ruktanonchai NW, Zhou L, Prosper O, Luo W, Floyd JR, et al. Effect of non-pharmaceutical interventions for containing the COVID-19 outbreak in China. medRxiv. 2020:2020.03.03.20029843.

58. Lam EHY, Cowling BJ, Cook AR, Wong JYT, Lau MSY, Nishiura H. The feasibility of age-specific travel restrictions during influenza pandemics. Theor Biol Med Model. 2011;8(1).

59. Lau H et al.. Positive impact of lockdown in Wuhan on containing the COVID-19 outbreak in China ;Journal of Travel Medicine; Oxford Academic. Journal of Travel Medicine. 2020.

60. Lee JM, Choi D, Cho G, Kim Y. The effect of public health interventions on the spread of influenza among cities. Journal of Theoretical Biology. 2012;293:131-42.


For peer review only

61. Li D, Liu Z, Liu Q, Gao Z, Zhu J, Yang J, et al. Estimating the Efficacy of Traffic Blockage and Quarantine for the Epidemic Caused by 2019-nCoV (COVID-19). medRxiv. 2020:2020.02.14.20022913.

62. Lin H, Liu W, Gao H, Nie J, Fan Q. Trends in Transmissibility of 2019 Novel Coronavirus-infected Pneumonia in Wuhan and 29 Provinces in China. medRxiv. 2020:2020.02.21.20026468.

63. Linka K, Peirlinck M, Sahli Costabal F, Kuhl E. Outbreak dynamics of COVID-19 in Europe and the effect of travel restrictions. medRxiv. 2020:2020.04.18.20071035.

64. Liu H, Bai X, Shen H, Pang X, Liang Z, Liu Y. Synchronized travel restrictions across cities can be effective in COVID-19 control. medRxiv. 2020a:2020.04.02.20050781.

65. Liu K, Ai S, Song S, Zhu G, Tian F, Li H, et al. Population movement, city closure in Wuhan and geographical expansion of the 2019-nCoV pneumonia infection in China in January 2020. Clinical infectious diseases. 2020.

66. Liu XN, Takeuchi Y. Spread of disease with transport-related infection and entry screening. Journal of Theoretical Biology. 2006;242(2):517-28.

67. Malmberg H, Britton T. Inflow restrictions can prevent epidemics when contact tracing efforts are effective but have limited capacity. medRxiv. 2020:2020.04.01.20050401.

68. Malone JD, Brigantic R, Muller GA, Gadgil A, Delp W, McMahon BH, et al. U.S. airport entry screening in response to pandemic influenza: Modeling and analysis. Travel Med Infect Dis. 2009;7(4):181-91.

69. Mandal S, Bhatnagar T, Arinaminpathy N, Agarwal A, Chowdhury A, Murhekar M, et al. Prudent public health intervention strategies to control the coronavirus disease 2019 transmission in India: A mathematical model-based approach. Indian Journal of Medical Research. 2020;23:23.

70. Marcelino J, Kaiser M. Critical paths in a metapopulation model of H1N1: Efficiently delaying influenza spreading through flight cancellation. PLoS Currents. 2012.

71. Mbuvha R, Marwala T. Bayesian Inference of COVID-19 Spreading Rates in South Africa. medRxiv. 2020:2020.04.28.20083873.

72. Mondal S, Ghosh S. Searching the Sigmoid-type trend in Lock Down period covid19 data of India and its different states. medRxiv. 2020:2020.04.25.20079624.

73. Moriarty L. et al., Prevention, et al. Public Health Responses to COVID-19 Outbreaks on Cruise Ships — Worldwide, February–March 2020. MMWR Morb Mortal Wkly Rep. 2020.

74. Mummert A, Weiss H, Long LP, Amigo JM, Wan XF. A perspective on multiple waves of influenza pandemics. PLoS ONE [Electronic Resource]. 2013;8(4):e60343.

75. Muraduzzaman AKM, Khan MH, Parveen R, Sultana S, Alam AN, Akram A, et al. Event based surveillance of Middle East Respiratory Syndrome Coronavirus (MERS- CoV) in Bangladesh among pilgrims and travelers from the Middle East: An update for the period 2013-2016. PLoS ONE [Electronic Resource]. 2018;13(1):e0189914.

For peer review only

76. Nakata Y, Rost G. Global analysis for spread of infectious diseases via transportation networks. Journal of Mathematical Biology. 2015;70(6):1411-56.

77. Nigmatulina KR, Larson RC. Living with influenza: Impacts of government imposed and voluntarily selected interventions. European Journal of Operational Research. 2009;195(2):613-27.

78. Nishiura H, Wilson N, Baker MG. Quarantine for pandemic influenza control at the borders of small island nations. BMC Infect Dis. 2009;9.

79. Odendaal WG. A Method to Model Outbreaks of New Infectious Diseases with Pandemic Potential such as COVID-19. medRxiv. 2020:2020.03.11.20034512.

80. Pan J, Yao Y, Liu Z, Li M, Wang Y, Dong W, et al. Effectiveness of control strategies for Coronavirus Disease 2019: a SEIR dynamic modeling study. medRxiv. 2020:2020.02.19.20025387.

81. Pan X, Ojcius DM, Gao T, Li Z, Pan C, Pan C. Lessons learned from the 2019-nCoV epidemic on prevention of future infectious diseases. Microbes Infect. 2020a;22(2):86-91.

82. Pang X, Zhu Z, Xu F, Guo J, Gong X, Liu D, et al. Evaluation of Control Measures Implemented in the Severe Acute Respiratory Syndrome Outbreak in Beijing, 2003. J Am Med Assoc. 2003;290(24):3215-21.

83. Pinkas J, Jankowski M, Szumowski L, Lusawa A, Zgliczynski WS, Raciborski F, et al. Public Health Interventions to Mitigate Early Spread of SARS-CoV-2 in Poland. Medical Science Monitor. 2020;26:e924730.

84. Pitman RJ, Cooper BS, Trotter CL, Gay NJ, Edmunds WJ. Entry screening for severe acute respiratory syndrome (SARS) or influenza: Policy evaluation. Br Med J. 2005;331(7527):1242-3.

85. Priest PC, Jennings LC, Duncan AR, Brunton CR, Baker MG. Effectiveness of border screening for detecting influenza in arriving airline travelers. American Journal of Public Health. 2013;103(8):1412-8.

86. Pullano G, Pinotti F, Valdano E, Boelle PY, Poletto C, Colizza V. Novel coronavirus (2019-nCoV) early-stage importation risk to Europe, January 2020. Euro Surveillance: Bulletin Europeen sur les Maladies Transmissibles = European Communicable Disease Bulletin.25(4):01.

87. Qiu Y, Chen X, Shi W. Impacts of social and economic factors on the transmission of coronavirus disease (COVID-19) in China. medRxiv. 2020:2020.03.13.20035238.

88. Quilty BJ, Diamond C, Liu Y, Gibbs H, Russell TW, Jarvis CI, et al. The effect of inter-city travel restrictions on geographical spread of COVID-19: Evidence from Wuhan, China. medRxiv. 2020:2020.04.16.20067504.

89. Ray D, Salvatore M, Bhattacharyya R, Wang L, Mohammed S, Purkayastha S, et al. Predictions, role of interventions and effects of a historic national lockdown in India's response to the COVID-19 pandemic: data science call to arms. medRxiv. 2020:2020.04.15.20067256.

For peer review only

90. Sakaguchi H, Tsunoda M, Wada K, Ohta H, Kawashima M, Yoshino Y, et al. Assessment of border control measures and community containment measures used in japan during the early stages of Pandemic (H1N1) 2009. PLoS ONE. 2012;7(2).

91. Samaan G, Patel M, Spencer J, Roberts L. Border screening for SARS in Australia: What has been learnt? Med J Aust. 2004;180(5):220-4.

92. Sang Z, Qiu ZP, Yan XF, Zou Y. Assessing the effect of non-pharmaceutical interventions on containing an emerging disease.. Mathematical Biosciences and Engineering.9(1):147-64.

93. Scala A, Flori A, Spelta A, ro, Brugnoli E, Cinelli M, et al. Between Geography and Demography: Key Interdependencies and Exit Mechanisms for Covid-19. medRxiv. 2020:2020.04.09.20059592.

94. Scalia Tomba G, Wallinga J. A simple explanation for the low impact of border control as a countermeasure to the spread of an infectious disease. Mathematical Biosciences.214(1):70-2.

95. Shi Z, Fang Y. Temporal relationship between outbound traffic from Wuhan and the 2019 coronavirus disease (COVID-19) incidence in China. medRxiv. 2020:2020.03.15.20034199.

96. Song PX, Wang L, Zhou Y, He J, Zhu B, Wang F, et al. An epidemiological forecast model and software assessing interventions on COVID-19 epidemic in China. medRxiv. 2020:2020.02.29.20029421.

97. St. John RK, King A, De Jong D, Bodie-Collins M, Squires SG, Tam TWS. Border screening for SARS. Emerging Infectious Diseases. 2005;11(1):6-10.

98. Su L, Hong N, Zhou X, He J, Ma Y, Jiang H, et al. Evaluation of the secondary transmission pattern and epidemic prediction of COVID-19 in the four metropolitan areas of China. medRxiv. 2020.

99. Tang B, Wang X, Li Q, Bragazzi NL, Tang S, Xiao Y, et al. Estimation of the Transmission Risk of the 2019-nCoV and Its Implication for Public Health Interventions. Journal of Clinical Medicine.9(2):07.

100. Tian H, Liu Y, Li Y, Wu C-H, Chen B, Kraemer MUG, et al. The impact of transmission control measures during the first 50 days of the COVID-19 epidemic in China. medRxiv. 2020:2020.01.30.20019844.

101. Tsuboi M, Hachiya M, Noda S, Iso H, Umeda T. Epidemiology and quarantine measures during COVID-19 outbreak on the cruise ship Diamond Princess docked at Yokohama, Japan in 2020: a descriptive analysis. Global Health & Medicine. 2020:2020.01037-2020.

102. Wang F, Li Y, Tang D, Li Q, Liu G, Wang J, et al. Trend of the Coronavirus Disease-2019 Epidemic in China After the Lockdown of Wuhan City on January 23, 2020. SSRN. 2020.

103. Wang L, Zhang Y, Huang T, Li X. Estimating the value of containment strategies in delaying the arrival time of an influenza pandemic: a case study of travel restriction and patient isolation. Physical Review E Statistical, Nonlinear, & Soft Matter Physics. 2012;86(3):032901.

For peer review only

104. Wang TH, Wei KC, Hsiung CA, Maloney SA, Eidex RB, Posey DL, et al. Optimizing severe acute respiratory syndrome response strategies: lessons learned from quarantine. American Journal of Public Health. 2007;97:S98-100.

105. Wang X, Liu S, Wang L, Zhang W. An Epidemic Patchy Model with Entry-Exit Screening. Bulletin of Mathematical Biology. 2015;77(7):1237-55.

106. Wells CR, Sah P, Moghadas SM, Pandey A, Shoukat A, Wang Y, et al. Impact of international travel and border control measures on the global spread of the novel 2019 coronavirus outbreak. Proc Natl Acad Sci U S A. 2020;117(13):7504-9.

107. Weng W, Ni S. Evaluation of containment and mitigation strategies for an influenza A pandemic in China. Simulation. 2015;91(5):407-16.

108. Wilder-Smith A, Goh KT, Paton NI. Experience of Severe Acute Respiratory Syndrome in Singapore: Importation of Cases, and Defense Strategies at the Airport. Journal of Travel Medicine. 2003;10(5):259-62.

109. Wood JG, Zamani N, Raina MacIntyre C, Becker NG. Effects of internal border control on spread of pandemic influenza. Emerging Infectious Diseases. 2007;13(7):1038-45.

110. Yang Z, Zeng Z, Wang K, Wong SS, Liang W, Zanin M, et al. Modified SEIR and AI prediction of the epidemics trend of COVID-19 in China under public health interventions. Journal of Thoracic Disease. 2020;12(3):165-74.

111. Ying S, Li F, Geng X, Li Z, Du X, Chen H, et al. Spread and control of COVID-19 in China and their associations with population movement, public health emergency measures, and medical resources. medRxiv. 2020:2020.02.24.20027623.

112. Yu H, Cauchemez S, Donnelly CA, Zhou L, Feng L, Xiang N, et al. Transmission dynamics, border entry screening, and school holidays during the 2009 influenza A (H1N1) pandemic, China. Emerging Infectious Diseases. 2012;18(5):758-66.

113. Yuan H-Y, Hossain MP, Tsegaye MM, Zhu X, Jia P, Wen T-H, et al. Estimating the risk on outbreak spreading of 2019-nCoV in China using transportation data. medRxiv. 2020:2020.02.01.20019984.

114. Yuan Z, Xiao Y, Dai Z, Huang J, Chen Y. A simple model to assess Wuhan lock-down effect and region efforts during COVID-19 epidemic in China Mainland. medRxiv. 2020a;2020a:2020.02.29.20029561.

115. Zhang B, Zhou H, Zhou F. Study on SARS-COV-2 transmission and the effects of control measures in China. medRxiv. 2020a:2020.02.16.20023770.

116. Zhang C, Chen C, Shen W, Tang F, Lei H, Xie Y, et al. Impact of population movement on the spread of 2019-nCoV in China. Emerging Microbes & Infections. 2020:1-28.

117. Zhang N, Zhao PC, Li YG. Increased infection severity in downstream cities in infectious disease transmission and tourists surveillance analysis. Journal of Theoretical Biology. 2019;470:20-9.

For peer review only



For peer review only - http://bmjopen.bmj.com/site/about/guidelines.xhtml

118. Zhang WD, Zu ZH, Xu Q, Xu ZJ, Liu JJ, Zheng T. Optimized Strategy for the Control and Prevention of Newly Emerging Influenza Revealed by the Spread Dynamics Model. PLoS ONE. 2014;9(1):11.

119. Zhang Y, Yang P, Liyanage S, Seale H, Deng Y, Pang X, et al. The characteristics of imported cases and the effectiveness of outbreak control strategies of pandemic influenza A (H1N1) in China. Asia-Pac J Public Health. 2012;24(6):932-9.

120. Zhao S, Chen H. Modeling the epidemic dynamics and control of COVID-19 outbreak in China. Quantitative Biology. 2020:1-9.

121. Zhou X, Wu Z, Yu R, Cao S, Fang W, Jiang Z, et al. Modeling-based evaluation of the effect of quarantine control by the Chinese government in the coronavirus disease 2019 outbreak. medRxiv. 2020:2020.03.03.20030445.

122. Zlojutro A, Rey D, Gardner L. A decision-support framework to optimize border control for global outbreak mitigation. Sci Rep. 2019;9(1).

For peer review only

**S6:** Interventions considered in the included studies

| Study ID | Short description of intervention | Phase of implementation |
|---|---|---|
| **Border closure** | | |
| **Studies assessing COVID-19** | | |
| Banholzer 2020 | Closure of national borders for individuals | Local transmission phase |
| **Studies assessing a hypothetical disease (COVID-19 relevant)** | | |
| Boyd 2018 | n.r. | Early phase |
| Boyd 2017 | Closure of national borders (ending the arrival of all commercial passenger planes, private planes, cruise ships, and yachts) | Early phase |
| **Entry/exit screening** | | |
| **Studies assessing COVID-19** | | |
| Clifford 2020 | • Entry screening (at departure)<br>• Exit screening (on arrival)<br>• Follow-up quarantine of positive cases<br>• Follow-up information/handouts to all arriving passengers | Early phase |
| Gostic 2020 | • Entry screening (all arriving passengers; temperature screening followed by a questionnaire on exposure risk)<br>• Exit screening (all departing passengers; temperature screening followed by a questionnaire on exposure risk)<br>• Follow up detainment of potentially infected passengers | Unclear phase |

| | Study ID | Short description of intervention | Phase of implementation |
|---|---|---|---|
| | Mandal 2020 | • Entry screening (passengers from China, Hong Kong, Singapore, Thailand, Japan, South Korea, Iran, and Italy; focus on temperature screening; advise for symptomatic passengers to volunteer for screening)<br>· Follow-up quarantine of positive cases | Early phase |
| | **Studies assessing influenza** | | |
| | Fujita 2011 | · Entry screening (on-board screening at Narita International Airport; questionnaire and thermography)<br>· Follow-up medical assessment (rapid tests followed by RT-PCR)<br>· Follow-up quarantine of positive cases and those sitting nearby<br>· Follow-up daily monitoring of travelers with negative screening results | Unclear phase |
| | Gunaratnam 2014 | · Entry screening (all international arrivals at Sydney Airport; health declaration and thermal scanning)<br>· Follow-up medical assessment (swab taken) | Unclear phase |
| | Hale 2012 | · Entry screening (all international arrivals at Auckland International Airport; health card, self-presentation)<br>· Follow up medical assessment (ILI case definition, RT-PCR)<br>· Quarantine of positive cases | Unclear phase |
| | Hien 2010 | · Entry screening (all international arrivals; temperature screening; symptom questionnaire)<br>· Follow up quarantine of suspected cases (in hospitals) | Local transmission phase |
| | Khan 2013 | · Entry screening (targeted: passengers arriving at international airports after flying from Mexico on a direct flight or indiscriminate entry screening on a direct or a connecting flight)<br>· Exit screening (passengers departing from any international airport in Mexico) | Unclear phase |
| | Kuo 2009 | · Entry screening (on-board screening of passengers arriving from epidemic affected areas, such as Mexico, USA, and Canada before passengers leave their seats; the procedure soon adjusted for only those airplanes reporting ill passengers on board)<br>· Follow-up quarantine of positive cases (hospital) | Unclear phase |

| Study ID | Short description of intervention | Phase of implementation |
|---|---|---|
| Malone 2009 | · Entry screening (all international passengers arriving at 18 US airports from Asia, Europe, South America and Canada: includes pre-arrival, primary active, and secondary active surveillance measures)<br>· Follow up quarantine of positive cases | Local transmission phase |
| Mummert 2013 | · Entry screening (thermal scanners)<br>· Follow-up quarantine of suspected cases and those seating nearby | Local transmission phase |
| Priest 2013 | · Entry screening (for all passengers arriving from Australia; all completed a health questionnaire, randomly chosen passengers (50%) screened for temperature and had throat and nasal swabs taken ) | Unclear phase |
| Sakaguchi 2012 | · Entry screening (all passengers arriving from other countries; infrared thermos-scanner, a health declaration form)<br>· Follow-up medical assessment (rapid influenza test, RT-PCR)<br>· Follow-up quarantine of positive cases and close contacts (seating next to them in the airplane)<br>· Follow-up information/handouts (all asymptomatic passengers)<br>· Follow-up health monitoring for fellow travelers of infected individuals identified during entry screening | Unclear phase |
| Wang 2015 | Different scenarios were considered in the model:<br>· Indiscriminate entry and exit screening in each patch<br>· Indiscriminate entry screening only in each patch<br>· Targeted entry screening (travel to and from high risk patch only)<br>· Selective entry screening (only the high risk patch and those closely connected patches)<br>· One-way entry screening in low-risk patches only | Local transmission phase |
| Yu 2012 | · Entry screening (all passengers at any point of entry into China from another country or from a neighboring region)<br>· Follow-up quarantine of suspected cases (in designated hospitals) | Local transmission phase; Unclear phase |
| Zhang 2014 | · Entry screening (temperature screening at ports of entry) | Early phase |
| Zhang 2012 | · Entry screening (temperature screening, questionnaire)<br>· Follow-up medical assessment (designated hospital)<br>· Quarantine of positive cases and close contacts | Unclear phase |

| Study ID | Short description of intervention | Phase of implementation |
|---|---|---|
| | · Medical follow-up of all travelers from overseas (daily contacts for 7 days)<br>· Follow-up ILI screening in hospitals | |
| Zlojutro 2018 | · Entry screening (in airports)<br>· Follow up quarantine of positive cases | Early phase |
| **Studies assessing SARS** | | |
| Anonymous | · Entry screening (all passengers arriving from affected areas; questionnaire, thermal image scanners)<br>· Exit screening (all departing passengers; thermal image scanners)<br>· Follow-up medical assessment (oral temperature screening, standardized questionnaire for SARS) | Local transmission phase;<br>Unclear phase |
| Glass 2006 | · Border screening (health declaration cards)<br>· Follow-up Information/handouts to travelers | Early phase;<br>Unclear phase |
| Goubar 2008 | · Entry screening (for symptomatic travelers only) | Early phase;<br>Unclear phase |
| Samaan 2004 | · Entry screening (identification through self-referral, detection by airline staff or airport custom staff; Australian Quarantine and Inspection Service conducting basic screening)<br>· Follow up screening (temperature screening by nursing staff)<br>· Follow-up screening (chief quarantine officer applies case definition)<br>· Follow-up medical assessment (in a hospital) | Early phase;<br>Local transmission phase |
| Pang 2003 | · Border screening (at the Beijing airport, major train stations, and 71 roads connecting Beijing to other areas) | Local transmission phase |
| Sang 2012 | · Entry screening | Unclear phase |
| St. John 2005 | · Entry screening (all arriving passengers; questionnaire, thermal scanners)<br>· Exit screening (all departing passengers; questionnaire, thermal scanners)<br>· Follow-up: Information/handouts to all arriving passengers<br>· Follow-up medical assessment<br>· Detainment and further medical assessment (in a hospital) | Unclear phase |

| Study ID | Short description of intervention | Phase of implementation |
| --- | --- | --- |
| Wilder-Smith 2003 | Different entry and exit screening procedures rolled out at different times:<br>· Entry screening (all passengers arriving from SARS-affected areas; visual screening and further temperature screening; health declaration)<br>· Entry screening (all arriving passengers in the airport and road entry points and further ferry terminals; temperature screening)<br>· Exit screening (all departing passengers in the airport and road entry points and further ferry terminals; temperature screening)<br>· Entry screening (all arriving passengers screened for SARS)<br>· Follow-up medical assessment | Early phase; (or post-peak phase) |
| **Studies assessing MERS** | | |
| Muraduzzaman, 2017 | · Entry screening (Bangladeshi travelers/pilgrims returning from the Middle East)<br>· Follow-up medical assessment<br>· Follow up quarantine of suspected cases | Unclear phase |
| **Studies assessing multiple diseases (SARS, MERS, and/or Influenza)** | | |
| Gostic 2015 | · Entry screening (all arriving passengers; temperature screening followed by a questionnaire on exposure risk)<br>· Exit screening (all departing passengers; temperature screening followed by a questionnaire on exposure risk)<br>· Follow up detainment of potentially infected passengers | Unclear phase |
| Pitman 2005 | · Entry screening | Unclear phase |
| **Studies assessing a hypothetical disease (COVID-19 relevant)** | | |
| Kim 2017 | n.r. | Unclear phase |
| Liu 2006 | · Entry screening<br>· Exit screening<br>· Follow-up quarantine of positive cases | Local transmission phase |
| **Travel ban** | | |



For peer review only - http://bmjopen.bmj.com/site/about/guidelines.xhtml

| Study ID | Short description of intervention | Phase of implementation |
|---|---|---|
| **Studies assessing COVID-19** | | |
| Adekunle 2020 | Ban on China, Iran, South Korea, and Italy | Local transmission phase |
| Chiyomaru 2020 | Three scenarios considered:<br>· Global ban<br>· Global quarantine<br>· Non-global restrictions | Local transmission phase |
| Costantino 2020 | Three scenarios modelled:<br>· No ban<br>· Current ban followed by a full lifting<br>· Partial lifting of the current ban (allowing over 100,000 university students to enter Australia, but not tourists) | Local transmission phase;<br>Post-peak phase |
| Mbuvha 2020 | n.r. | Local transmission phase |
| Mondal 2020 | Shutting of airlines and rail transport in India | Local transmission phase |
| Pullano 2020 | Ban on international flights in Hubei (as an example) | Early phase |
| Ray 2020 | · Main scenario: national lockdown<br>· Alternative scenario A: mild action: social distancing and travel ban<br>· Alternative scenario B: no action | Local transmission phase |
| **Studies assessing influenza** | | |
| Hsieh 2007 | n.r. | Local transmission phase |
| **Travel-related quarantine** | | |
| **Studies assessing COVID-19** | | |

| Study ID | Short description of intervention | Phase of implementation |
|---|---|---|
| Moriatry 2020 | Quarantine of travelers (14 days either on the ship or on the land (port of Oakland) before disembarkation or onward travel) | Local transmission phase |
| Tsuboi 2020 | Quarantine of all travelers in their cabins (followed by targeted screening for COVID-19) | Unclear phase |
| **Studies assessing influenza** | | |
| Nishiura 2009 | Quarantine of travelers (all individuals arriving from the country in which the outbreak occurred) | Early phase |
| **Studies assessing SARS** | | |
| de Vlas 2009 | Quarantine of travelers (in airports and stations) | Early phase;<br>Local transmission phase;<br>Post-peak phase |
| Hsieh 2006 | Home quarantine of travelers (anyone who entered the country from affected countries) | Local transmission phase |
| Wang 2007 | Quarantine of travelers (those sitting on the same flight within 3 rows of a person infected with SARS or those returning from WHO–designated SARS-affected areas) | Unclear phase |
| **Travel restrictions** | | |
| **Studies assessing COVID-19** | | |
| Chang 2020 | n.r. | Early phase;<br>Local transmission phase |
| Odendaal 2020 | n.r. | Early phase |

| Study ID | Short description of intervention | Phase of implementation |
|---|---|---|
| Scala 2020 | n.r. | Early phase;<br>Local transmission phase;<br>Post-peak phase |
| **Studies assessing influenza** | | |
| Bolton 2012 | n.r. | Local transmission phase |
| Chong 2012 | Restricting air, land, and maritime travel | Local transmission phase |
| Ciofi 2008 | Restricting air travel | Early phase |
| Colizza 2007 | Restricting air travel | Early phase |
| Cooper 2005 | Restricting air travel | Local transmission phase |
| Eichner 2009 | Restricting air travel | Early phase |
| Epstein 2007 | Restricting air travel | Early phase |
| Flahault 2006 | Restricting air travel | Early phase |
| Germann 2006 | Restricting air travel | Local transmission phase |
| Kerneis 2008 | Restricting air travel | Early phase |
| Lam 2011 | n.r. | Early phase |
| Lee 2012 | n.r. | Early phase |
| Marcelino 2013 | Restricting air travel | Early phase;<br>Local transmission phase |
| Nigmatulina 2009 | n.r. | Early phase;<br>Local transmission phase |

| Study ID | Short description of intervention | Phase of implementation |
|---|---|---|
| Scalia Tomba 2008 | n.r. | Early phase |
| Wang 2012 | Cancellation of non-essential trips | Early phase |
| Weng 2015 | n.r. | Early phase;<br>Local transmission phase |
| Wood 2007 | Restricting air travel | Early phase |
| **Studies assessing multiple diseases (SARS and influenza)** | | |
| Chung 2015 | Restricting (control measures on) air travel | Unclear phase |
| Hollingsworth 2006 | Restricting air travel | Early phase |
| **Studies assessing a hypothetical disease (COVID-19 relevant)** | | |
| Arino 2007 | n.r. | Early phase |
| Nakata 2015 | n.r. | Early phase;<br>Local transmission phase |
| **Multiple travel-related control measures** | | |
| **Studies assessing COVID-19** | | |
| Aleta 2020 | Lockdown of Wuhan (referred in the study as "Wuhan's quarantine") | Local transmission phase |
| Anzai 2020 | "Travel restriction and border control have been implemented by various countries, either as: (i) complete travel bans, (ii) travel restriction and quarantine—which allows for restriction of healthy individuals, (iii) entry screening for all incoming travelers, or some combination thereof" | Local transmission phase |
| Arima 2020 | Entry/exit screening | Unclear phase |

| Study ID | Short description of intervention | Phase of implementation |
|---|---|---|
| | · Entry screening (repatriation of nationals from China and oropharyngeal swab sample taken for all)<br>· Follow-up quarantine of positive cases (in a hospital)<br>Travel-related quarantine (all returning from China for 14 days) | |
| Cheng 2020 | Entry/exit screening<br>· Entry screening (on-board temperature screening for all travelers from Wuhan)<br>· Follow-up medical assessment (in designated hospitals)<br>Travel-related quarantine (all returning from Wuhan, Guangdong, Zheijiang, China, Hong Kong, Macau, and later from South Korea and Italy) | Local transmission phase |
| Chinazzi 2020 | n.r. | Local transmission phase |
| Cowling 2020 | Travel ban<br>· Ban on Hubei residents and visitors to Hubei province in the past 14 days<br>· Ban on flights (between Hong Kong and Wuhan)<br>· Ban on entry of non-Hong Kong residents from overseas countries and territories<br>Travel restrictions<br>· Restricting air travel<br>· Restricting land travel<br>Border closure<br>Travel-related quarantine (for travelers from Hubei, then China, South Korea, Iran, Italy, Germany, Japan and Spain, all Schengen area, all overseas countries, Macau and Taiwan). | Local transmission phase |
| Dandekar 2020 | n.r. | Local transmission phase |
| Ediriweera 2020 | · Lockdown (island-wide)<br>· Travel-related quarantine (all inbound passengers) | Local transmission phase |
| Fang 2020 | Lockdown of Wuhan | Local transmission phase |
| Hamidouche 2020 | Travel ban<br>· Ban: air travel (China, Italy, Morocco, France)<br>· Ban: local public transport including rail traffic | Local transmission phase |

| Study ID | Short description of intervention | Phase of implementation |
|---|---|---|
| | Closure of all land borders and air routes<br>Travel-related quarantine (14 days) | |
| He 2020 | n.r. | Local transmission phase |
| Hossain 2020 | Lockdown of Wuhan (referred in the study as "lockdown, border control, or quarantine measures"). | Local transmission phase |
| Hou 2020 | Lockdown of Wuhan | Local transmission phase |
| Jia 2020 | Lockdown of Wuhan (referred in the study as "quarantine of Wuhan") | Early phase;<br>Local transmission phase |
| Jiang 2020 | n.r. (referred in the study as "travel restrictions in Hubei") | Local transmission phase |
| Kraemer 2020 | Lockdown of Wuhan (referred in the study as "cordon sanitaire imposed on Wuhan") | Local transmission phase |
| Lai 2020 | Lockdown of Wuhan | Early phase;<br>Local transmission phase |
| Lau 2020 | Lockdown of Wuhan | Early phase;<br>Local transmission phase |
| Li 2020 | Lockdown of Wuhan (referred in the study as "quarantine and traffic blockage") | Early phase;<br>Local transmission phase |
| Lin 2020 | Lockdown of Wuhan (referred in the study as "travel bans into and from Wuhan") | Local transmission phase |
| Linka 2020 | n.r. | Early phase;<br>Local transmission phase |
| Liu 2020 | Lockdown of Wuhan (referred in the study as "city closure") | Early phase;<br>Local transmission phase |



For peer review only - http://bmjopen.bmj.com/site/about/guidelines.xhtml

| Study ID | Short description of intervention | Phase of implementation |
|---|---|---|
| Liu 2020a | Lockdown of Wuhan and travel restrictions in other cities | Early phase;<br>Local transmission phase |
| Pan 2020 | Various interventions strategies in China, including travel restrictions and voluntary quarantine | Local transmission phase |
| Pan 2020a | "Strict traffic restrictions" implemented by the Chinese government. | Local transmission phase |
| Pinkas 2020 | · Border screening (passengers location cards, temperature screening)<br>· Closure of borders to non-citizens<br>· Travel ban: all non-essential travel; suspension of all international air and rail services<br>· Travel-related quarantine | Local transmission phase |
| Qiu 2020 | · Lockdown of Wuhan and other cities in Hubei<br>· Imposed travel restrictions, ranging from "canceling public events, stopping public transportation to limiting how often residents can leave home" | Early phase;<br>Local transmission phase |
| Quilty 2020 | · Lockdown of Wuhan (referred in the study as "cordon sanitaire on the city of Wuhan")<br>· Travel restrictions extended to the whole of Hubei | Local transmission phase |
| Shi 2020 | Lockdown of Wuhan (referred in the study as "Wuhan travel ban") | Local transmission phase |
| Song 2020 | Lockdown of Wuhan (referred in the study as "community-level quarantines such as hospitalization for infected cases, city blockade, traffic control and restricted social activities, and so on") | Local transmission phase |
| Su 2020 | "Official quarantine regulations and travel restrictions in China" | Local transmission phase |
| Tang 2020 | Lockdown of Wuhan and other cities in China (referred in the study as "travel restrictions affecting five cities (Wuhan, Huanggang, Ezhou, Chibi and Zhijiang), effectively shutting down the movement of more than 40 million people") | Local transmission phase |
| Tian 2020 | Lockdown of Wuhan and other cities in China ("China banned travel to and from Wuhan city on 23 January and implemented a national emergency response") | Local transmission phase |

| Study ID | Short description of intervention | Phase of implementation |
|---|---|---|
| Wang 2020 | Lockdown of Wuhan (referred in the study as "quarantine of Wuhan city") | Local transmission phase |
| Wells 2020 | · Lockdown of Wuhan and some other cities in China<br>· Entry/exit screening in airports (questionnaire, self-reporting) | Local transmission phase |
| Yang 2020 | Lockdown of Wuhan and other cities in China (referred in the study as "large-scale quarantine, strict controls on travel and extensive monitoring of suspected cases) | Local transmission phase |
| Ying 2020 | Lockdown of Wuhan and other cities in Hubei | Local transmission phase |
| Yuan 2020 | Lockdown of Wuhan<br>Two scenarios were considered:<br>· Border control measures without any other control (no mask wearing, isolation, or contact tracing)<br>· Border control with some other control measures | Early phase;<br>Local transmission phase |
| Yuan 2020a | Lockdown of Wuhan "combined with nationwide traffic restrictions" | Local transmission |
| **Studies assessing influenza** | | |
| Bajardi 2011 | Multiple scenarios modelled:<br>· No travel restrictions<br>· Travel ban: air travel (country-specific)<br>· Travel restrictions: land travel | Early phase |
| Caley 2007 | Entry/exit screening<br>· Exit screening (all passengers at departure)<br>· Follow up detainment (those positive are detained and prevented from traveling)<br>· Entry screening (all passengers at arrival)<br>· Follow-up quarantine of all travelers if a positive case is detected<br>Travel restriction: air travel | Early phase |
| Ferguson 2006 | Border controls<br>Travel restriction | Local transmission phase |
| Zhang 2019 | Entry/exit screening | Early phase |



For peer review only - http://bmjopen.bmj.com/site/about/guidelines.xhtml

| Study ID | Short description of intervention | Phase of implementation |
|---|---|---|
| | · Entry screening (city-entry surveillance)<br>· Exit screening (city-exit surveillance)<br>· Follow up quarantine of positive cases<br>Travel reduction across cities (decrease in human mobility) | |
| Zhang 2020 | Lockdown of Wuhan and other cities in Hubei (referred in the study as "citywide quarantine of Wuhan and several nearby cities") | Local transmission phase |
| Zhang 2020a | Lockdown of Wuhan and other cities in Hubei ("Chinese government and people moved quickly to take measures to control the sources of infection and block the routes of transmission") | Early phase;<br>Local transmission phase |
| Zhao 2020 | Lockdown of Wuhan (referred to in the study as "a quarantine") | Early phase;<br>Local transmission phase;<br>Post-peak phase |
| Zhou 2020 | Lockdown of Wuhan (referred in the study as "strict control policies, including lockdown of Wuhan city") | Local transmission phase |
| **Studies assessing multiple diseases (COVID-19 and influenza)** | | |
| Kong 2020 | Lockdown of Wuhan ("Wuhan was locked down and China raised the national public health response to the first state of emergency") | Local transmission phase |
| **Studies assessing a hypothetical disease (COVID-19 relevant)** | | |
| Malmberg 2020 | · Inflow travel restrictions<br>· Quarantine of travelers (all those arriving) | Local transmission phase;<br>Post-peak phase |

**S7:** Outcome categories and specific outcomes assessed in studies

| Category | Outcomes included | Number of studies |
|---|---|---|
| **Infectious disease outcomes** | | |
| Number or proportion of cases | Number of cases (predicted, reported, detected) | 58 |
| | Number of cases as rates per e.g. 1000, 100000 population | 3 |
| | Number of imported/exported cases | 4 |
| | Number of cases at epidemic peak | 1 |
| | Attack rate | 7 |
| Number or proportion of deaths | Number of deaths | 6 |
| | Case fatality rate | 1 |
| Healthcare resources | Number of cases requiring ICU treatment | 1 |
| | Time until ICU capacity is reached | 1 |
| Reproduction number | Effective reproduction number | 12 |
| | Basic reproduction number | 5 |
| Temporal development of epidemic | Time to epidemic arrival | 22 |
| | Time to epidemic peak | 9 |
| | Time to 1000 cases | 2 |
| | Doubling rate | 1 |
| Probability of epidemic | Probability of epidemic | 4 |
| | Probability of avoiding epidemic | 1 |
| | Probability of sustained transmission | 1 |
| | Probability of releasing infected individuals | 1 |
| | Probability of developing symptoms | 1 |
| Other infectious disease outcomes | Transmission risk index | 1 |
| | Transmission routes | 1 |
| | Number of infected cities | 1 |
| **Screening outcomes** | | |
| Detection of high risk persons or cases | Number or proportion screened that are identified as high risk | 10 |
| | Number or proportion screened that are diagnosed | 12 |
| | Number or proportion quarantined that are diagnosed | 3 |
| | Positive predictive value of screening measures/tests | 2 |
| | Probability of missing cases | 4 |
| **Economic outcomes** | | |
| Costs | Value of measures | 2 |
| | Cost of measures as percentage of Gross Domestic Product | 1 |
| | Cost of preventing cases | 1 |
| Industry impact | Air traffic passenger volumes | 1 |
| **Social outcomes** | | |
| Acceptability of measures | Attitudes towards travel restrictions | 1 |



For peer review only - http://bmjopen.bmj.com/site/about/guidelines.xhtml

**S8:** Overview of types of studies included in the evidence map

**Inferential studies**

**Compartmental models**
Common in infectious disease research, these include, for example the SIR model (comprising three compartments, S: susceptible, I: infectious, R: recovered). Several included studies applied such models, and extended the SIR model by adding other compartments, such as E: exposed, NS: non-susceptible, L: latent, I: isolated, D: dead

Examples from included studies: Kim et al. 2017; Mandal et al. 2020; Mbuvha et al. 2020

**Spatial models**
Several included studies applied models to explore how disease transmission moves spatially. Such models use assumptions and/or data about how humans move to predict disease transmission. Included studies examined, for example, mobility data from smartphone usage or global flight patterns.

Examples from included studies: Bajardi et al. 2011; Chinazzi et al. 2020; Clifford et al. 2020; Colizza 2020; Kraemer et al. 2020; Pullano et al. 2020

**Epidemiological time-series models**
Many included studies investigated the temporal nature of the disease data, and applied time-series approaches common to epidemiology. They applied a range of approaches, including simple time-series analysis, auto-regressive integrated moving average models, and change-point analysis to explore changes over time.

Examples from included studies: Chung et al. 2015; Jiang et al. 2020; Lin 2020_trends; Yuan 2020a

**Other epidemiological techniques**
Included studies also used more simple epidemiological designs and and statistical methods, such as correlation analysis, linear regression and statistical testing (e.g. chi-square test).

Examples from included studies: Fujita et al. 2011; Lau et al. 2020; Wang et al. 2020d

**Mixed approaches**
Several included studies used a range of approaches; combinations included compartmental models and spatial models, compartmental models and time-series models.

Examples from included studies: Epstein et al. 2007; Linka et al. 2020; Longxiang et al. 2020; Marcelino et al. 2013; Wang et al. 2020

**Descriptive studies**

**Observational studies**

Several included studies described the impact of control measures through summary statistics; these comprised, for example, the number or proportion of screened individuals that were classified as high risk and the number or proportion of screened individuals that were diagnosed.

Examples from included studies: Arima et al. 2020; Hale et al. 2020; Kuo et al. 2009; Sakaguchi et al. 2012

**Graphical summary studies**

A small number of included studies used graphical summary techniques to describe the impact of control measures. These illustrated, for example, how the number of cases developed in time relative to the introduction of control measures.

Examples from included studies: Cheng et al. 2020; de Vlas et al. 2009; Pinkas et al. 2020

For peer review only



For peer review only - http://bmjopen.bmj.com/site/about/guidelines.xhtml

## Supplementary file

## S1: Preferred Reporting Items for Systematic reviews and Meta-Analyses extension for Scoping Reviews (PRISMA-ScR) Checklist

| SECTION | ITEM | PRISMA-ScR CHECKLIST ITEM | REPORTED ON PAGE # |
|---|---|---|---|
| **TITLE** | | | |
| Title | 1 | Identify the report as a scoping review. | |
| **ABSTRACT** | | | |
| Structured summary | 2 | Provide a structured summary that includes (as applicable): background, objectives, eligibility criteria, sources of evidence, charting methods, results, and conclusions that relate to the review questions and objectives. | |
| **INTRODUCTION** | | | |
| Rationale | 3 | Describe the rationale for the review in the context of what is already known. Explain why the review questions/objectives lend themselves to a scoping review approach. | |
| Objectives | 4 | Provide an explicit statement of the questions and objectives being addressed with reference to their key elements (e.g., population or participants, concepts, and context) or other relevant key elements used to conceptualize the review questions and/or objectives. | |
| **METHODS** | | | |
| Protocol and registration | 5 | Indicate whether a review protocol exists; state if and where it can be accessed (e.g., a Web address); and if available, provide registration information, including the registration number. | |
| Eligibility criteria | 6 | Specify characteristics of the sources of evidence used as eligibility criteria (e.g., years considered, language, and publication status), and provide a rationale. | |
| Information sources* | 7 | Describe all information sources in the search (e.g., databases with dates of coverage and contact with authors to identify additional sources), as well as the date the most recent search was executed. | |
| Search | 8 | Present the full electronic search strategy for at least 1 database, including any limits used, such that it could be repeated. | |
| Selection of sources of evidence† | 9 | State the process for selecting sources of evidence (i.e., screening and eligibility) included in the scoping review. | |
| Data charting process‡ | 10 | Describe the methods of charting data from the included sources of evidence (e.g., calibrated forms or forms that have been tested by the team before their use, and whether data charting was done independently or in duplicate) and any processes for obtaining and confirming data from investigators. | |
| Data items | 11 | List and define all variables for which data were sought and any assumptions and simplifications made. | |
| Critical appraisal of individual sources of evidence§ | 12 | If done, provide a rationale for conducting a critical appraisal of included sources of evidence; describe the methods used and how this information was used in any data synthesis (if appropriate). | |
| Synthesis of results | 13 | Describe the methods of handling and summarizing the data that were charted. | |

St. Michael's
Inspired Care. Inspiring Science.

For peer review only - http://bmjopen.bmj.com/site/about/guidelines.xhtml

| SECTION | ITEM | PRISMA-ScR CHECKLIST ITEM | REPORTED ON PAGE # |
|---|---|---|---|
| **RESULTS** | | | |
| Selection of sources of evidence | 14 | Give numbers of sources of evidence screened, assessed for eligibility, and included in the review, with reasons for exclusions at each stage, ideally using a flow diagram. | |
| Characteristics of sources of evidence | 15 | For each source of evidence, present characteristics for which data were charted and provide the citations. | |
| Critical appraisal within sources of evidence | 16 | If done, present data on critical appraisal of included sources of evidence (see item 12). | |
| Results of individual sources of evidence | 17 | For each included source of evidence, present the relevant data that were charted that relate to the review questions and objectives. | |
| Synthesis of results | 18 | Summarize and/or present the charting results as they relate to the review questions and objectives. | |
| **DISCUSSION** | | | |
| Summary of evidence | 19 | Summarize the main results (including an overview of concepts, themes, and types of evidence available), link to the review questions and objectives, and consider the relevance to key groups. | |
| Limitations | 20 | Discuss the limitations of the scoping review process. | |
| Conclusions | 21 | Provide a general interpretation of the results with respect to the review questions and objectives, as well as potential implications and/or next steps. | |
| **FUNDING** | | | |
| Funding | 22 | Describe sources of funding for the included sources of evidence, as well as sources of funding for the scoping review. Describe the role of the funders of the scoping review. | |

JBI = Joanna Briggs Institute; PRISMA-ScR = Preferred Reporting Items for Systematic reviews and Meta-Analyses extension for Scoping Reviews.

* Where *sources of evidence* (see second footnote) are compiled from, such as bibliographic databases, social media platforms, and Web sites.

† A more inclusive/heterogeneous term used to account for the different types of evidence or data sources (e.g., quantitative and/or qualitative research, expert opinion, and policy documents) that may be eligible in a scoping review as opposed to only studies. This is not to be confused with *information sources* (see first footnote).

‡ The frameworks by Arksey and O'Malley (6) and Levac and colleagues (7) and the JBI guidance (4, 5) refer to the process of data extraction in a scoping review as data charting.

§ The process of systematically examining research evidence to assess its validity, results, and relevance before using it to inform a decision. This term is used for items 12 and 19 instead of "risk of bias" (which is more applicable to systematic reviews of interventions) to include and acknowledge the various sources of evidence that may be used in a scoping review (e.g., quantitative and/or qualitative research, expert opinion, and policy document).

*From:* Tricco AC, Lillie E, Zarin W, O'Brien KK, Colquhoun H, Levac D, et al. PRISMA Extension for Scoping Reviews (PRISMAScR): Checklist and Explanation. Ann Intern Med. 2018;169:467–473. doi: 10.7326/M18-0850.


For peer review only - http://bmjopen.bmj.com/site/about/guidelines.xhtml

BMJ Open

# Travel-related control measures to contain the COVID-19 pandemic: an evidence map



| | |
|---|---|
| Journal: | *BMJ Open* |
| Manuscript ID | bmjopen-2020-041619.R1 |
| Article Type: | Original research |
| Date Submitted by the Author: | 09-Nov-2020 |





| | |
|---|---|
| Complete List of Authors: | Burns, Jacob; Ludwig Maximilians University Munich, Institute for Medical Information Processing, Biometry and Epidemiology; Ludwig Maximilians University Munich, Pettenkofer School of Public Health<br>Movsisyan, Ani; Ludwig Maximilians University Munich, Institute for Medical Information Processing, Biometry and Epidemiology; Ludwig Maximilians University Munich, Pettenkofer School of Public Health<br>Biallas, Renke; Ludwig Maximilians University Munich, Institute for Medical Information Processing, Biometry and Epidemiology; Ludwig Maximilians University Munich, Pettenkofer School of Public Health<br>Coenen, Michaela; Ludwig Maximilians University Munich, Institute for Medical Information Processing, Biometry and Epidemiology; Ludwig Maximilians University Munich, Pettenkofer School of Public Health<br>Geffert, Karin; Ludwig Maximilians University Munich, Institute for Medical Information Processing, Biometry and Epidemiology; Ludwig Maximilians University Munich, Pettenkofer School of Public Health<br>Horstick, Olaf; Heidelberg University, Heidelberg Institute of Global Health<br>Klerings, Irma; Danube University Krems, Department for Evidence-based Medicine and Evaluation<br>Pfadenhauer, Lisa; Ludwig Maximilians University Munich, Institute for Medical Information Processing, Biometry and Epidemiology; Ludwig Maximilians University Munich, Pettenkofer School of Public Health<br>von Philipsborn, Peter; Ludwig Maximilians University Munich, Institute for Medical Information Processing, Biometry and Epidemiology; Ludwig Maximilians University Munich, Pettenkofer School of Public Health<br>Sell, Kerstin; Ludwig Maximilians University Munich, Institute for Medical Information Processing, Biometry and Epidemiology; Ludwig Maximilians University Munich, Pettenkofer School of Public Health<br>Strahwald, Brigitte; Ludwig Maximilians University Munich, Institute for Medical Information Processing, Biometry and Epidemiology; Ludwig Maximilians University Munich, Pettenkofer School of Public Health<br>Stratil, Jan; Ludwig Maximilians University Munich, Institute for Medical Information Processing, Biometry and Epidemiology; Ludwig Maximilians University Munich, Pettenkofer School of Public Health<br>Voss, Stephan; Ludwig Maximilians University Munich, Institute for Medical Information Processing, Biometry and Epidemiology; Ludwig Maximilians University Munich, Pettenkofer School of Public Health<br>Rehfuess, Eva; Ludwig Maximilians University Munich, Institute for Medical Information Processing, Biometry and Epidemiology, Pettenkofer School of Public Health; Ludwig Maximilians University Munich, Pettenkofer School of Public Health |

| | |
|---|---|
| <b>Primary Subject Heading</b>: | Public health |
| Secondary Subject Heading: | Global health, Public health, Infectious diseases |
| Keywords: | Public health < INFECTIOUS DISEASES, INFECTIOUS DISEASES, Diabetic foot < DIABETES & ENDOCRINOLOGY |
| | |

SCHOLARONE™
Manuscripts

*I, the Submitting Author has the right to grant and does grant on behalf of all authors of the Work (as defined in the below author licence), an exclusive licence and/or a non-exclusive licence for contributions from authors who are: i) UK Crown employees; ii) where BMJ has agreed a CC-BY licence shall apply, and/or iii) in accordance with the terms applicable for US Federal Government officers or employees acting as part of their official duties; on a worldwide, perpetual, irrevocable, royalty-free basis to BMJ Publishing Group Ltd ("BMJ") its licensees and where the relevant Journal is co-owned by BMJ to the co-owners of the Journal, to publish the Work in this journal and any other BMJ products and to exploit all rights, as set out in our licence.*

*The Submitting Author accepts and understands that any supply made under these terms is made by BMJ to the Submitting Author unless you are acting as an employee on behalf of your employer or a postgraduate student of an affiliated institution which is paying any applicable article publishing charge ("APC") for Open Access articles. Where the Submitting Author wishes to make the Work available on an Open Access basis (and intends to pay the relevant APC), the terms of reuse of such Open Access shall be governed by a Creative Commons licence – details of these licences and which Creative Commons licence will apply to this Work are set out in our licence referred to above.*

*Other than as permitted in any relevant BMJ Author's Self Archiving Policies, I confirm this Work has not been accepted for publication elsewhere, is not being considered for publication elsewhere and does not duplicate material already published. I confirm all authors consent to publication of this Work and authorise the granting of this licence.*

For peer review only

# Travel-related control measures to contain the COVID-19 pandemic: an evidence map

Jacob Burns*[1,2], Ani Movsisyan*[1,2], Renke Biallas[1,2], Michaela Coenen[1,2], Karin Geffert[1,2], Olaf Horstick[3], Irma Klerings[4], Lisa Maria Pfadenhauer[1,2], Peter von Philipsborn [1,2], Kerstin Sell[1,2], Brigitte Strahwald[1,2], Jan M Stratil[1,2], Stephan Voss[1,2], Eva Rehfuess [1,2]

* These authors have contributed equally.

[1] Institute for Medical Information Processing, Biometry and Epidemiology; Ludwig-Maximilians-University Munich, Germany

[2] Pettenkofer School of Public Health; Ludwig-Maximilians-University, Munich, Germany

[3] Heidelberg Institute of Global Health, Heidelberg University, Germany

[4] Department for Evidence-based Medicine and Evaluation, Danube University Krems, Austria

Corresponding author:
Ani Movsisyan
Institute of Medical Information Processing, Biometry, and Epidemiology
Pettenkofer School of Public Health
LMU Munich, Marchioninistrasse 17, 81377 Munich, Germany
ani.movsisyan@ibe.med.uni-muenchen.de
+49 17685245374

Keywords: Travel restrictions, SARS-CoV-2, COVID-19, pandemic, evidence map.

Word count: 5294

For peer review only

## Abstract

**Objectives**: To comprehensively map the existing evidence assessing the impact of travel-related control measures for containment of the SARS-CoV-2/COVID-19 pandemic.

**Design**: Rapid evidence map.

**Data sources**: MEDLINE, Embase and Web of Science, and COVID-19-specific databases offered by the US Centers for Disease Control and Prevention and the World Health Organization.

**Eligibility criteria**: We included studies in human populations susceptible to SARS-CoV-2/COVID-19, SARS-CoV-1/SARS, MERS-CoV/MERS, or influenza. Interventions of interest were travel-related control measures affecting travel across national or sub-national borders. Outcomes of interest included infectious disease, screening, other health, economic and social outcomes. We considered all empirical studies which quantitatively evaluate impact available in Armenian, English, French, German, Italian, and Russian, based on the team's language capacities.

**Data extraction and synthesis**: We extracted data from included studies in a standardized manner, and mapped them to a priori and (one) post-hoc defined categories.

**Results**: We included 122 studies assessing travel-related control measures. These studies were undertaken across the globe, most in the Western Pacific Region (n=71). A large proportion of studies focused on COVID-19 (n=59), but a number of studies also examined SARS, MERS, and influenza. We identified studies on border closures (n=3), entry/exit screening (n=31), travel-related quarantine (n=6), travel bans (n=8), and travel restrictions (n=25). Many addressed a bundle of travel-related control measures (n=49). Most studies assessed infectious disease (n=98) and/or screening-related (n=25) outcomes; we found only limited evidence on economic and social outcomes. Studies applied numerous methods, both inferential and descriptive in nature, ranging from simple observational methods to complex modeling techniques.

**Conclusions**: We identified a heterogeneous and complex evidence base on travel-related control measures. While this map is not sufficient to assess the effectiveness of different measures, it outlines aspects regarding interventions and outcomes, as well as study methodology and reporting which could inform future research and evidence synthesis.

For peer review only

**Strengths and limitations of this study**

- We applied systematic and standardized methods at all stages, and have produced a comprehensive evidence map of travel-related control measures for containment of the SARS-CoV-2/COVID-19 pandemic.
- For title/abstract and full text screening, we developed guidance documents and conducted piloting exercises to ensure consistency among review authors; we additionally collected and clarified all uncertainties on a rolling basis through daily team calls.
- We conducted searches in three major health-related databases and two COVID-specific databases; however, it is likely that most studies assessing economic and social outcomes are found in other databases.
- The unspecific and inconsistent reporting of primary studies with regard to interventions, especially when a package of control measures was investigated, meant that determining eligibility, as well as summarizing and mapping these studies was challenging.
- We did not include travel warning or travel advice in the evidence map, which limits the scope of this map.

For peer review only

## Introduction

In December 2019 the occurrence of the novel coronavirus, SARS-CoV-2, was reported in Wuhan, China. Over the next weeks the virus and the associated respiratory disease referred to as COVID-19 spread further into China and other parts of Asia including Japan, South Korea, and Thailand (1). By mid-March 2020, when the World Health Organization (WHO) declared COVID-19 a global pandemic, cases had been observed in over 100 countries and territories across the globe (2).

According to WHO, various travel-related control measures, such as entry/exit screening, travel bans to and/or from specific areas within or between countries, and quarantine of travelers have since been implemented by most countries around the world to contain and mitigate the spread of SARS-CoV-2 and COVID-19 (3). Following the early-stage responses in Asian countries, strict measures, such as border closures and drastic reductions in airline travel, have been put into place in most countries around the world, starting in February 2020 and continuing into May 2020. While in the context of a rapidly evolving pandemic decisions often need to be made even in the absence of high quality evidence, efforts to identify and synthesize the best-available evidence will help inform whether the currently implemented measures should be sustained, adapted, or lifted. Where possible, decisions need to be based on evidence regarding the effectiveness of these measures in contributing to the control of the pandemic, as well as regarding the associated economic and social impacts. They will also need to take into account short- and longer-term costs, acceptability, and feasibility of such measures.

Travel-related control measures have been assessed through systematic or narrative reviews in the context of previous epidemics and pandemics, such as influenza, severe acute respiratory syndrome (SARS-CoV-1/SARS), and Middle East Respiratory Syndrome (MERS-CoV/MERS). Regarding the containment of influenza, these reviews have examined the effectiveness of a broad set of measures (4-6), as well as the effectiveness of specific measures, such as entry/exit screening (6, 7); they have also assessed the economic implications of various pharmaceutical and non-pharmaceutical interventions during influenza pandemics (8, 9). Effectiveness of measures, such as international travel bans and entry/exit screening have also been examined in reviews of SARS and MERS (7, 10). To date and to the best of our knowledge, the evidence on travel-related control measures for the control of the current pandemic has not been systematically assessed.

In this paper we aim to systematically identify and map the existing evidence assessing the impact of travel-related control measures (i.e. border closures, travel restrictions and bans, entry and exit screening, quarantine/isolation of travelers crossing borders, multiple interventions combined) for containment of the SARS-CoV-2/COVID-19 pandemic, drawing on evidence from the current pandemic, as well as on evidence in relation to SARS, MERS, and influenza. This evidence map was commissioned by the World Health Organization to serve as an important resource for researchers and policy-makers in providing an overview of the currently available evidence in relation to various travel-related control measures to contain the COVID-19 pandemic. In itself, the evidence map is not sufficient to assess the effectiveness of different measures. Instead, it provides an important basis for decisions regarding the need for and possibility to conduct primary research as well as more specific evidence synthesis (e.g. regarding a specific category of control measures or a specific type of studies).

For peer review only

# Methods

## Search strategy

We designed the evidence map in accordance with the Preferred Reporting Items for Systematic Reviews and Meta-Analyses extension for Scoping Reviews (PRISMA-ScR) reporting guideline, and implemented the entire project within ten days (see online supplementary file S1 for the completed PRISMA-ScR checklist). We searched the following databases: (i) Ovid MEDLINE(R) ALL (1946-present), (ii) Embase.com (Elsevier), (iii) Science Citation Index Expanded (1900-present), Social Sciences Citation Index (1900-present), Emerging Sources Citation Index (2015-present) (Web of Science), (iv) the US Centers for Disease Control and Prevention's COVID-19 Research Articles Downloadable Database (includes published articles, as well as grey literature, such as preprints), and (v) WHO COVID-19 Database (includes published articles only).

The initial search strategy was developed for MEDLINE (see online supplementary file S2) and further adapted for the other databases. All database searches were conducted up to 3 May 2020. The search strategies were designed and conducted by an experienced information specialist. The searches were conducted in English. Where database functionality allowed for it, we limited the search results to Armenian, English, French, German, Italian, and Russian, based on the language capacity of the research team, and considered studies for inclusion published in all of these languages. We also conducted forward and backward citation searches of all relevant (systematic) reviews identified through the database searches (see online supplementary file S3) up to 6 May 2020 in Scopus (Elsevier).

## Eligibility criteria

We determined eligibility of studies based on the investigated population/context, intervention, outcome, and study type.

Regarding the population/context, this evidence map draws on direct evidence from human populations susceptible to the current SARS-CoV-2/COVID-19, as well as indirect evidence from a set of other relevant respiratory infectious diseases. To help define a set of diseases that is most similar and relevant to COVID-19, we used the following criteria: (i) diseases of viral origin; (ii) mode of transmission primarily airborne via droplets/aerosols (as well as person-to-person); (iii) acute disease with the potential to cause an epidemic/pandemic; (iv) similar clinical features (i.e. nonspecific febrile illness with the potential to develop into pneumonia and acute respiratory distress syndrome; difficult diagnosis based on clinical features during transmission-relevant phase, including the period prior to symptom development and during early-stage symptoms); (v) unavailability of a vaccine and/or difficulty to contain an outbreak through vaccination. As a result, we considered:

- SARS-CoV-2/COVID-19
- SARS-CoV-1/SARS
- MERS-CoV/MERS
- Influenza

For peer review only

Studies in all other populations/contexts, including evidence on infectious diseases less relevant to the current SARS-CoV-2/COVID-19 pandemic (e.g. avian influenza, Ebola, meningitis, HIV/AIDS, tuberculosis, dengue, plague, cholera, fever, smallpox, measles, or zika virus) were excluded.

Regarding the intervention, we considered travel-related control measures affecting human travel across national or sub-national borders, specifically:

- Closure of borders (borders closed to entry and/or exit)
- Travel bans (suspension of flights, ground crossing, ship itineraries, refusal of entry or travel, visa suspension/denial) between countries, and between regions and large cities within countries
- Travel restrictions (i.e., varying levels of travel reductions) between countries, and between regions and large cities within countries)
- Entry/exit screening (e.g. temperature measurement, health questionnaire, thermography, physical examination, laboratory tests, passive observation and/or follow-up quarantine) at airports, ports, land borders, and train stations
- Quarantine/isolation of travelers from affected regions (at borders, at designated institutions or at home)
- Any combination of the above measures and/or with other control measures (e.g. combination of border closure and school closure).

All other interventions were excluded. This comprised those not directly related to travel (e.g. community-based quarantine, hygiene measures, bans on mass gatherings), those related to the movement of animals or goods, travel warnings or travel advice issued by the WHO or national governments, situations affecting travel but not representing travel-related control measures (e.g. school holidays), and those solely concerned with the effectiveness of laboratory tests rather than their implementation as part of an entry/exit screening procedure.

Regarding outcomes, we considered those assessing the quantitative impacts of the interventions on:

- Infectious disease outcomes (e.g. number/proportion of cases, number/proportion of deaths, time to/delay in epidemic arrival or peak, reproduction number, healthcare demand and utilization)
- Screening outcomes (e.g. number/proportion of persons screened, number/proportion of those screened identified as cases)
- Other health outcomes (e.g. psychosocial impact)
- Economic outcomes (e.g. travel volumes, costs of measures implemented, losses to different economic sectors)
- Social outcomes (e.g. stigmatization/discrimination of foreigners, xenophobia, migration volumes)

All other outcomes, such as those on the human rights and legal implications of interventions, were excluded.

Regarding study types, we considered all types of empirical studies which quantitatively evaluate impact (e.g. epidemiologic, modeling, simulations, and econometric studies). All other study types, including qualitative studies, diagnostic studies focused on test performance and non-empirical studies (e.g. commentaries, narrative and systematic reviews), were excluded.



For peer review only - http://bmjopen.bmj.com/site/about/guidelines.xhtml

## Study selection

After de-duplication, all titles and abstracts were screened by one reviewer (shared among several team members), excluding only those studies which were clearly irrelevant. For all studies deemed potentially relevant or unclear at the title/abstract screening stage, one reviewer screened the full text. At this stage, a final decision regarding eligibility was made. We adopted a very inclusive approach: any unclear cases were discussed with a second reviewer, and remaining uncertainties were resolved with involvement of a third reviewer and/or the whole review team. In addition, all studies excluded at the full text screening stage based on the intervention (i.e. not addressing travel-related control measures) were double-checked by a second reviewer to make sure that no relevant studies were excluded.

We used Endnote to manage collection and de-duplication of records. For title and abstract screening, we used Rayyan, a web-based application designed for citation screening for systematic reviews (11). We documented reasons for the exclusion of full texts and reported those using Microsoft Excel.

For both the title/abstract and full text screening stages, we developed screening guidance forms to ensure that all reviewers screen similarly and consistently. We discussed inconsistencies and challenges encountered within the review team, after having screened approximately 300 titles/abstracts and 50 full texts, and subsequently refined the screening guidance. We additionally collected and clarified all uncertainties in screening on a rolling basis. These were discussed in daily online meetings to ensure consistency in screening across multiple reviewers and that any questions and comments were addressed.

## Data extraction

One reviewer extracted study characteristics and data into the pre-defined categories of the data extraction form in Microsoft Excel (see online supplementary file S4). The extraction form was pilot-tested in the review team. The following categories were covered by the extraction form: population, setting, and context; characterization of the respiratory pathogen/disease (i.e. SARS CoV-2/COVID-19, SARS CoV-1/SARS, MERS CoV/MERS, influenza), types of interventions (e.g. entry/exit screening, border closure, quarantine of travelers, and travel bans), comparisons (where available), outcomes of interest (e.g. health, economic, and social impact), study designs (e.g. epidemiologic study, modeling study).

## Mapping

We charted the extracted data based on categories and present findings in a tabular, narrative, or graphical manner. Most of these categories were defined a priori, while one category, namely, geographical setting was adapted post hoc. Specifically, we sought to define, summarize, and present clusters of studies based on the pathogen/disease (i.e. SARS-CoV-2/COVID-19, SARS-CoV-1/SARS, MERS-CoV/MERS, influenza), type of intervention (border closure, entry/exist screening including follow-up measures, such as isolation of positive cases, travel ban, travel-related quarantine in the absence of entry/exit screening, travel restrictions, and multiple travel-related control measures combined), timing of the intervention (i.e. early phase, local transmission phase, post-peak phase, unclear phase), outcomes of interest (i.e. infectious disease outcomes, screening outcomes, other


For peer review only - http://bmjopen.bmj.com/site/about/guidelines.xhtml

health outcomes, economic and social outcomes) and study designs (e.g. epidemiologic study, modeling study). All data presented in the tables, text and graphics were double-checked by a second reviewer with an emphasis on accuracy in reporting on populations, interventions and outcomes and in relation to consistency of presentation.

## Patient and public involvement

Considering the time constraints and the nature of this research (i.e., a rapid systematic evidence map), it was conducted without patient and public involvement.

# Results

Database searches yielded a total of 4,928 unique records. Through snowballing of reviews identified through the database searches, we identified an additional 1,700 studies. During the title/abstract screening stage, we excluded 4,445 records as clearly irrelevant. We subsequently assessed the full texts of 483 records and excluded another 361 records. Overall, we included 122 studies in this evidence map (see online supplementary file S5 for the full list). Of these, 80 were journal articles, 41 were pre-prints and 1 was a report. Figure 1 provides an overview of our searching and screening procedures.

## Characteristics of included studies

The 122 included studies were characterized by substantial heterogeneity in relation to the countries and populations targeted, the diseases addressed, the types of travel-related control measures examined (sometimes assessed against a range of other interventions to contain an epidemic/pandemic), and the numerous outcomes assessed. They comprise both studies that are inferential in nature and studies that are descriptive in nature, varying greatly in the specific methods applied; notably, many of the studies addressing COVID-19 were pre-prints. We summarize these aspects below, and provide a description of each study in Table 1.

**Countries and settings**

Included studies assessed interventions across the globe, including, according to WHO world regions, from the Western Pacific Region (WPR) (n=71), the South-East Asia region (SEAR) (n=5), the Region of the Americas (AMR) (n=12), the European region (EUR) (n=8), and the African Region (AFR) (n=2). None of the studies included in this evidence map was conducted in a country of the Eastern Mediterranean Region (EMR). The specific countries in which interventions were implemented are listed in Table 1 (under "regions protected by travel related measure"). Several modeling studies looked at hypothetical regions (n=16).

Of the identified interventions, some were implemented in island states (n=24) or in what we define quasi-island states (n=9), such as the UK or Hong Kong, which have limited land borders connected through, for example, a tunnel. Most interventions were implemented in non-island states (n=74). The remaining interventions assessed hypothetical regions, some of which were framed as comparable to island states, while others were more general in their framing and assumptions.



For peer review only - http://bmjopen.bmj.com/site/about/guidelines.xhtml

**Table 1**: Characteristics of included studies

| Study[1] | Study type | WHO Region | Geographical setting | Region protected by travel-related measure | Region restricted by travel-related measure | Disease | Intervention category | Outcome type |
|---|---|---|---|---|---|---|---|---|
| Adekunle 2020 (pre-print) | Inferential | WPR | Island state | Australia | China, Iran, South Korea, Italy | COVID-19 | Travel ban | Number or proportion of cases |
| Aleta 2020 (pre-print) | Inferential | WPR | Non-island state | Regions within China | Wuhan | COVID-19 | Multiple travel-related control measures | Number or proportion of cases |
| Anonymous | Descriptive | AMR | Non-island state | Vancouver, Toronto, other parts of Canada | All other countries | SARS | Entry/exit screening | Detection of high risk persons or cases |
| Anzai 2020 | Inferential | WPR | Non-island state | Multiple countries | China | COVID-19 | Multiple travel-related control measures | Number or proportion of cases; Temporal development of epidemic |
| Arima 2020 | Descriptive | WPR | Island state | Japan | China | COVID-19 | Entry/exit screening; Travel-related quarantine | Detection of high risk persons or cases |
| Arino 2007 | Inferential | n.a. | Hypothetical | Hypothetical | Hypothetical | Hypothetical infectious disease | Travel restriction | Number or proportion of cases |
| Bajardi 2011 | Inferential | All | Non-island state | All countries | Mexico | Influenza (H1N1 2009 pandemic) | Travel ban; Travel restriction: land | Temporal development of epidemic |
| Banholzer 2020 (pre-print) | Inferential | EUR; WPR; AMR | Non-island state | Austria, Australia, Belgium, Canada Denmark, France, Finland, Italy, | All other countries | COVID-19 | Border closure | Number or proportion of cases |

[1] The full reference list of included studies is presented in Supplementary file S5.

| Study[1] | Study type | WHO Region | Geographical setting | Region protected by travel-related measure | Region restricted by travel-related measure | Disease | Intervention category | Outcome type |
|---|---|---|---|---|---|---|---|---|
| | | | | Norway, Switzerland, Sweden, Spain, and US | | | | |
| Bolton 2012 | Inferential | WPR | Non-island state | Mongolia | Neighboring countries | Influenza (H1N1 2009 pandemic) | Travel restriction | Number or proportion of cases |
| Boyd 2018 | Inferential | WPR | Island state | New Zealand | All other countries | Hypothetical infectious disease | Border closure | Costs |
| Boyd 2017 | Inferential | WPR | Island state | New Zealand | All other countries | Hypothetical infectious disease | Border closure | Costs |
| Caley 2007 | Inferential | n.a. | Hypothetical | Hypothetical | Hypothetical | Influenza | Entry/exit screening; Travel restriction: air | Detection of high risk persons or cases; Temporal development of epidemic |
| Chang 2020 (pre-print) | Inferential | WPR | Island state | Taiwan | Different cities in Taiwan | COVID-19 | Travel restriction | Number or proportion of cases; Temporal development of epidemic |
| Cheng 2020 | Descriptive | WPR | Island state | Taiwan | China | COVID-19 | Exit/entry screening; Travel-related quarantine | Number or proportion of cases |
| Chinazzi 2020 | Inferential | WPR | Non-island state | Other regions of China, all other countries | Wuhan | COVID-19 | Multiple travel-related control measures | Number or proportion of cases |
| Chiyomaru 2020 (pre-print) | Inferential | | Non-island state | Multiple countries | Multiple countries | COVID-19 | Travel ban | Number or proportion of cases |

| Study[1] | Study type | WHO Region | Geographical setting | Region protected by travel-related measure | Region restricted by travel-related measure | Disease | Intervention category | Outcome type |
|---|---|---|---|---|---|---|---|---|
| Chong 2012 | Inferential | WPR | Quasi-island state | Hong Kong | All other countries | Influenza (H1N1 2009 pandemic) | Travel restriction: land, air, and maritime | Number or proportion of cases; Temporal development of epidemic |
| Chung 2015 | Inferential | | Non-island state | Multiple countries | Multiple countries | SARS; 2006 Avian influenza; 2009 Swine flu H1N1 | Travel restriction: air | Industry impact |
| Ciofi 2008 | Inferential | EUR | Non-island state | Italy | Italy and all other countries | Influenza | Travel restriction: air | Number or proportion of cases; Temporal development of epidemic |
| Clifford 2020 (pre-print) | Inferential | n.a. | Hypothetical | Hypothetical | All other countries | COVID-19 | Entry/exit screening | Detection of high risk persons or cases; Temporal development of epidemic |
| Colizza 2007 | Inferential | All | Non-island state | All countries | All countries | Influenza | Travel restriction: air | Number or proportion of cases; Temporal development of epidemic |
| Cooper 2005 | Inferential | All | Non-island state | All countries | All countries | Influenza | Travel restriction: air | Temporal development of epidemic |
| Costantino 2020 (pre-print) | Inferential | WPR | Island state | Australia | All other countries | COVID-19 | Travel ban | Number or proportion of cases; Number or proportion of deaths; Temporal development of epidemic |
| Cowling 2020 | Inferential | WPR | Quasi-island state | Hong Kong | China, South Korea, Iran, Italy and affected regions in | COVID-19 | Travel restriction: air and land; Travel ban; | Reproduction number; Number or proportion of cases; Acceptability |

| Study[1] | Study type | WHO Region | Geographical setting | Region protected by travel-related measure | Region restricted by travel-related measure | Disease | Intervention category | Outcome type |
|---|---|---|---|---|---|---|---|---|
| | | | | | France, Germany, Japan, and Spain, Schengen Area, Macau, and Taiwan. | | Travel-related quarantine; Border closure | |
| Dandekar 2020 (pre-print) | Inferential | WPR, EUR, AMR | Island state; Quasi-island state | China, Italy, South Korea, US | All other countries | COVID-19 | Multiple travel-related control measures | Reproduction number |
| de Vlas 2009 | Descriptive | WPR | Non-island state | Regions of China | Regions of China | SARS | Travel-related quarantine | Reproduction number |
| Ediriweera 2020 (pre-print) | Inferential | SEAR | Island state | Sri Lanka | All other countries | COVID-19 | Multiple travel-related control measures; Travel-related quarantine | Number or proportion of cases; Healthcare resources |
| Eichner 2009 | Inferential | WPR | Hypothetical | Hypothetical | All other countries | Influenza (pandemic) | Travel restriction: air | Probability of epidemic |
| Epstein 2007 | Inferential | All | Non-island state | All countries (focus on US) | All countries | Influenza | Travel restriction: air | Number or proportion of cases; Temporal development of epidemic |
| Fang 2020 (pre-print) | Inferential | WPR | Non-island state | Other regions of China | Wuhan | COVID-19 | Multiple travel-related control measures | Number or proportion of cases |
| Ferguson 2006 | Inferential | AMR | Island state; Quasi-island state | US and UK | Countries connected by air traffic | Influenza (pandemic) | Border controls; Travel restrictios | Number or proportion of cases; Temporal development of epidemic |
| Flahault 2006 | Inferential | n.a. | Quasi-island state | 52 international cities and surrounding regions | Cities connected via air traffic | Influenza (pandemic) | Travel restriction: air | Number or proportion of cases |

| Study[1] | Study type | WHO Region | Geographical setting | Region protected by travel-related measure | Region restricted by travel-related measure | Disease | Intervention category | Outcome type |
|---|---|---|---|---|---|---|---|---|
| Fujita 2011 | Inferential | WPR | Island state | Regions in Japan | US, Canada, and Mexico | Influenza (H1N1 2009 pandemic) | Entry/exit screening | Detection of high risk persons or cases |
| Germann 2006 | Inferential | AMR | Non-island state | US | | Influenza (pandemic) | Travel restriction: air | Number or proportion of cases |
| Glass 2006 | Inferential | n.a. | Non-island state | Non-infected region | Non-infected region | SARS | Entry/exit screening | Probability of epidemic |
| Gostic 2020 | Inferential | All | Island state | All countries | All countries | COVID-19 | Entry/exit screening | Detection of high risk persons or cases |
| Gostic 2015 | Inferential | n.a. | Hypothetical | Hypothetical | Hypothetical | SARS; MERS; Influenza (H1N1 2009 pandemic) | Entry/exit screening | Detection of high risk persons or cases |
| Goubar 2008 | Inferential | EUR | Island state; Quasi-island state | Germany and UK | China, Hong Kong | SARS | Entry/exit screening | Number or proportion of cases |
| Gunaratnam 2014 | Descriptive | WPR | Island state | Australia | All other countries | Influenza (H1N1 2009 pandemic) | Entry/exit screening | Detection of high risk persons or cases |
| Hale 2012 | Descriptive | WPR | Island state | New Zealand | Initially US, Canada, Mexico, then all other countries | Influenza (H1N1 2009 pandemic) | Entry/exit screening | Detection of high risk persons or cases |
| Hamidouche 2020 (pre-print) | Inferential | AFR | Non-island state | Algeria | All neighboring and connected (via air) countries | COVID-19 | Border closure; Travel ban; Travel-related quarantine | Number or proportion of cases; Reproduction number |
| He 2020 (pre-print) | Inferential | WPR | Island state; Quasi-island state | China, South Korea, Italy, Iran | Italy (Lombardy, Veneto, Emilia-Romagna, Piedmont and | COVID-19 | Multiple travel-related control measures | Number or proportion of cases; Temporal development of epidemic |



For peer review only - http://bmjopen.bmj.com/site/about/guidelines.xhtml

| Study[1] | Study type | WHO Region | Geographical setting | Region protected by travel-related measure | Region restricted by travel-related measure | Disease | Intervention category | Outcome type |
|---|---|---|---|---|---|---|---|---|
| | | | | | Marche), and China (Zhejiang). | | | |
| Hien 2010 | Inferential | WPR | Non-island state | Vietnam | Countries with confirmed cases of 2009 H1N1 | Influenza (H1N1 2009 pandemic) | Entry/exit screening | Detection of high risk persons or cases |
| Hollingsworth 2006 | Inferential | n.a. | Hypothetical | Hypothetical | Countries connected via air traffic | SARS and Influenza | Travel restriction: air | Number or proportion of cases; Temporal development of epidemic |
| Hossain 2020 (pre-print) | Inferential | WPR | Non-island state | Other regions of China | Wuhan | COVID-19 | Multiple travel-related control measures | Temporal development of epidemic |
| Hou 2020 (pre-print) | Inferential | WPR | Non-island state | Other regions of China | Wuhan | COVID-19 | Multiple travel-related control measures | Number or proportion of cases; Reproduction number |
| Hsieh 2007 | Inferential | n.a. | Hypothetical | Hypothetical | Hypothetical | Influenza (endemic) | Travel ban | Reproduction number |
| Hsieh 2006 | Inferential | WPR | Island state | Taiwan | Countries with SARS cases | SARS | Travel-related quarantine | Number or proportion of cases |
| Jia 2020 | Inferential | WPR | Non-island state | Other regions of China | Wuhan | COVID-19 | Multiple travel-related control measures | Number or proportion of cases; Other |
| Jiang 2020 (pre-print) | Inferential | WPR | Non-island state | Regions of China | Regions of China | COVID-19 | Multiple travel-related control measures | Other |
| Kerneis 2008 | Inferential | n.a. | Non-island state | 52 international cities and surrounding regions | 52 international cities and surrounding regions | Influenza (pandemic) | Travel restriction: air | Number or proportion of cases; Temporal development of epidemic |
| Khan 2013 | Inferential | AMR | Non-island state | Other countries | Mexico | Influenza | Entry/exit screening | Detection of high risk |

| Study[1] | Study type | WHO Region | Geographical setting | Region protected by travel-related measure | Region restricted by travel-related measure | Disease | Intervention category | Outcome type |
|---|---|---|---|---|---|---|---|---|
| | | | | | | (H1N1 2009 pandemic) | | persons or cases; Other |
| Kim 2017 | Inferential | n.a. | Hypothetical | Hypothetical | Hypothetical | Hypothetical infectious disease | Entry/exit screening | Reproduction number |
| Kong 2020 (pre-print) | Inferential | WPR | Non-island state | Other regions of China | Wuhan and other cities in Hubei | COVID-19 and influenza | Multiple travel-related control measures | Number or proportion of cases |
| Kraemer 2020 | Inferential | WPR | Non-island state | Other regions of China | Wuhan and other cities in Hubei | COVID-19 | Multiple travel-related control measures | Number or proportion of cases |
| Kuo 2009 | Descriptive | WPR | Island state | Taiwan | All affected countries (e.g., Mexico, USA, and Canada) | Influenza (H1N2 2009 pandemic) | Entry/exit screening | Detection of high risk persons or cases |
| Lai 2020 (pre-print) | Inferential | WPR | Non-island state | Other regions of China | Wuhan | COVID-19 | Multiple travel-related control measures | Number or proportion of cases |
| Lam 2011 | Inferential | WPR | Quasi-island state | Hong Kong | All other countries | Influenza (H1N1 2009 pandemic) | Travel restriction | Probability of epidemic; Temporal development of epidemic |
| Lau 2020 (pre-print) | Inferential | WPR | Non-island state | Other regions of China | Wuhan and other cities in Hubei | COVID-19 | Multiple travel-related control measures | Number or proportion of cases; Temporal development of epidemic |
| Lee 2012 | Inferential | n.a. | Hypothetical | Hypothetical | Hypothetical | Influenza (H1N1 2009 pandemic) | Travel restriction | Temporal development of epidemic |
| Li 2020 (pre-print) | Inferential | WPR | Non-island state | Other regions of China | Wuhan and other cities in Hubei | COVID-19 | Multiple travel-related control | Number or proportion of cases |



For peer review only - http://bmjopen.bmj.com/site/about/guidelines.xhtml

| Study[1] | Study type | WHO Region | Geographical setting | Region protected by travel-related measure | Region restricted by travel-related measure | Disease | Intervention category | Outcome type |
|---|---|---|---|---|---|---|---|---|
| | | | | | | | measures | |
| Lin 2020 (pre-print) | Inferential | WPR | Non-island state | Other regions of China | Wuhan | COVID-19 | Multiple travel-related control measures | Reproduction number; Other |
| Linka 2020 (pre-print) | Inferential | EUR | Non-island state | All European countries | All European countries | COVID-19 | Multiple travel-related control measures | Number or proportion of cases |
| Liu 2006 | Inferential | n.a. | Hypothetical | Hypothetical | Hypothetical | Hypothetical infectious disease | Entry/exit screening | Reproduction number |
| Liu 2020 | Inferential | WPR | Non-island state | Other regions of China | Wuhan | COVID-19 | Multiple travel-related control measures | Number or proportion of cases |
| Liu 2020a (pre-print) | Inferential | WPR | Non-island state | Other Regions of China | Cities outside of Hubei | COVID-19 | Multiple travel-related control measures | Number or proportion of cases |
| Malmberg 2020 (pre-print) | Inferential | n.a. | Hypothetical | Hypothetical | Hypothetical | Hypothetical infectious disease | Travel restriction; Travel-related quarantine | Probability of epidemic |
| Malone 2009 | Inferential | AMR | Non-island state | US | Asia | Influenza (pandemic) | Entry/exit screening | Detection of high risk persons or cases; Number or proportion of cases; Number of deaths |
| Mandal 2020 | Inferential | SEAR | Non-island state | India | China, Hong Kong, Singapore, Thailand, Japan, South Korea, Iran, and Italy | COVID-19 | Entry/exit screening | Temporal development of epidemic; Number or proportion of cases |
| Marcelino 2013 | Inferential | All | Non-island state | All countries | Mexico city | Influenza | Travel restriction: | Number or proportion |

| Study[1] | Study type | WHO Region | Geographical setting | Region protected by travel-related measure | Region restricted by travel-related measure | Disease | Intervention category | Outcome type |
|---|---|---|---|---|---|---|---|---|
| | | | | | | (pandemic) | air | of cases |
| Mbuvha 2020 (pre-print) | Inferential | AFR | Non-island state | South Africa | South Africa and the all other countries | COVID-19 | Travel ban | Number or proportion of cases; Temporal development of epidemic |
| Mondal 2020 (pre-print) | Inferential | SEAR | Non-island state | India | India | COVID-19 | Travel ban | Number or proportion of cases |
| Moriatry 2020 | Inferential | WPR, AMR | Non-island state | Japan, US, home countries of cruise ship passengers | Home countries of cruise ships | COVID-19 | Travel-related quarantine | Detection of high risk persons or cases |
| Mummert 2013 | Inferential | AMR | Non-island state | US | Mexico | Influenza (H1N1 2009 pandemic) | Entry/exit screening | Number or proportion of cases |
| Muraduzzaman 2017 | Descriptive | SEAR | Non-island state | Bangladesh | Saudi Arabia | MERS | Entry/exit screening | Detection of high risk persons or cases |
| Nakara 2015 | Inferential | n.a. | Hypothetical | Hypothetical | Hypothetical | Hypothetical infectious disease | Travel restriction | Reproduction number |
| Nigmatulina 2009 | Inferential | n.a. | Hypothetical | Hypothetical | Hypothetical | Influenza | Travel restriction | Number or proportion of cases |
| Nishiura 2009 | Inferential | n.a. | Hypothetical | Hypothetical | Hypothetical | Influenza (pandemic) | Travel-related quarantine | Detection of high risk persons or cases |
| Odendaal 2020 (pre-print) | Inferential | AMR | Non-island state | US | China, and other affected countries | COVID-19 | Travel restriction | Number or proportion of cases |
| Pan 2020 | Inferential | WPR | Non-island state | Other regions of China | Wuhan and other cities in Hubei | COVID-19 | Multiple travel-related control measures | Number or proportion of cases |
| Pan 2020a (pre-print) | Inferential | WPR | Non-island state | China | China | COVID-19 | Multiple travel-related control measures | Number or proportion of cases; Reproduction number |



For peer review only - http://bmjopen.bmj.com/site/about/guidelines.xhtml

| Study[1] | Study type | WHO Region | Geographical setting | Region protected by travel-related measure | Region restricted by travel-related measure | Disease | Intervention category | Outcome type |
|---|---|---|---|---|---|---|---|---|
| Pang 2003 | Descriptive | WPR | Non-island state | Beijing | All other countries | SARS | Entry/exit screening | Number or proportion of cases |
| Pinkas 2020 | Descriptive | EUR | Non-island state | Poland | All other countries | COVID-19 | Entry/exit screening; Border closure; Travel ban; Travel-related quarantine | Number or proportion of cases |
| Pitman 2005 | Descriptive | EUR | Quasi-island state | UK | Any of the top 100 sources of international airline passengers | SARS, Influenza | Entry/exit screening | Detection of high risk persons or cases |
| Priest 2013 | Descriptive | WPR | Island state | New Zealand | Australia | Influenza (type A and B) | Entry/exit screening | Detection of high risk persons or cases |
| Pullano 2020 | Inferential | WPR | Non-island state | Other regions of China and European countries | Wuhan | COVID-19 | Travel ban | Probability of epidemic |
| Qiu 2020 (pre-print) | Inferential | WPR | Non-island state | Other regions of China | Wuhan and other cities in Hubei | COVID-19 | Multiple travel-related control measures | Number or proportion of cases |
| Quilty 2020 (pre-print) | Inferential | WPR | Non-island state | Beijing, Chongqing, Hangzhou, and Shenzhen | Wuhan | COVID-19 | Multiple travel-related control measures | Number or proportion of cases; Probability of epidemic |
| Ray 2020 (pre-print) | Inferential | SEAR | Non-island state | India | All other countries | COVID-19 | Travel ban | Number or proportion of cases |
| Sakaguchi 2012 | Descriptive | WPR | Island state | Japan | Mexico, US, and Canada | Influenza (A H1N1 2009 pandemic) | Entry/exit screening | Detection of high risk persons or cases |
| Samaan 2004 | Descriptive | WPR | Island state | Australia | All countries affected by SARS | SARS | Entry/exit screening | Detection of high risk persons or cases |

| Study[1] | Study type | WHO Region | Geographical setting | Region protected by travel-related measure | Region restricted by travel-related measure | Disease | Intervention category | Outcome type |
|---|---|---|---|---|---|---|---|---|
| Sang 2012 | Inferential | n.a. | Hypothetical | Hypothetical | Hypothetical | SARS | Entry/exit screening | Reproduction number; Temporal development of epidemic; Number or proportion of cases; Number or proportion of deaths |
| Scala 2020 (pre-print) | Inferential | EUR | Non-island state | Individual regions of Italy | Individual regions of Italy | COVID-19 | Travel restriction | Temporal development of epidemic |
| Scalia Tomba 2008 | Inferential | n.a. | Hypothetical | Hypothetical | Hypothetical | Influenza (pandemic) | Travel restriction/border control | Temporal development of epidemic |
| Shi 2020 (pre-print) | Inferential | WPR | Non-island state | Other regions of China | Wuhan | COVID-19 | Multiple travel-related control measures | Number or proportion of cases |
| Song 2020 (Wang 2020) | Inferential | WPR | Non-island state | Other regions of China | Wuhan | COVID-19 | Multiple travel-related control measures | Number or proportion of cases; Reproduction number |
| St. John 2005 | Descriptive | AMR | Non-island state | Canada | All other countries | SARS | Entry/exit screening | Detection of high risk persons or cases |
| Su 2020 (pre-print) | Inferential | WPR | Non-island state | Beijng, Shanghai, Guangzhou, and Shenzhen | Beijng, Shanghai, Guangzhou, and Shenzhen | COVID-19 | Multiple travel-related control measures | Number or proportion of cases; Temporal development of epidemic |
| Tang 2020 | Inferential | WPR | Non-island state | Beijing | Wuhan and other cities in China | COVID-19 | Multiple travel-related control measures | Number or proportion of cases |
| Tian 2020 (pre-print) | Inferential | WPR | Non-island state | Other regions of China | Wuhan and other cities in China | COVID-19 | Multiple travel-related control measures | Number or proportion of cases; Temporal development of epidemic |



For peer review only - http://bmjopen.bmj.com/site/about/guidelines.xhtml

| Study[1] | Study type | WHO Region | Geographical setting | Region protected by travel-related measure | Region restricted by travel-related measure | Disease | Intervention category | Outcome type |
|---|---|---|---|---|---|---|---|---|
| Tsuboi 2020 | Descriptive | WPR | Island state | Japan | Diamond Princess | COVID-19 | Travel-related quarantine | Number or proportion of quarantined diagnosed |
| Wang 2015 | Inferential | n.a. | Hypothetical | Hypothetical (low-risk patches) | Hypothetical (high-risk patch) | Influenza (H1N1 2009 pandemic) | Entry/exit screening | Reproduction number |
| Wang 2020 (pre-print) | Inferential | WPR | Non-island state | China | Wuhan | COVID-19 | Multiple travel-related control measures | Number or proportion of cases; Number or proportion of deaths |
| Wang 2007 | Descriptive | WPR | Island state | Taiwan | All SARS-affected regions | SARS | Travel-related quarantine | Detection of high risk persons or cases diagnosed |
| Wang 2012 | Inferential | WPR | Non-island state | Different regions in China | Different regions in China | Influenza (pandemic) | Travel restriction | Temporal development of epidemic |
| Wells 2020 | Inferential | WPR | Non-island state | All other countries | China | COVID-19 | Multiple travel-related control measures | Number or proportion of cases; Probability of epidemic |
| Weng 2015 | Inferential | WPR | Hypothetical | Hypothetical | Hypothetical | Influenza (H1N1 2009 pandemic) | Travel restriction | Number or proportion of cases |
| Wilder-Smith 2003 | Descriptive | WPR | Non-island state | Singapore | All SARS-affected regions | SARS | Entry/exit screening | Detection of high risk persons or cases |
| Wood 2007 | Inferential | WPR | Non-island state | Melbourne and Sydney | Sydney and Darwin | Influenza | Travel restriction: air | Temporal development of epidemic |
| Yang 2020 | Inferential | WPR | Non-island state | Other regions of China, particularly Guangdong and Zhjiang provinces | Hubei | COVID-19 | Multiple travel-related control measures | Number or proportion of cases; Temporal development of epidemic |
| Ying 2020 (pre-print) | Inferential | WPR | Non-island state | Other regions of China | Hubei | COVID-19 | Multiple travel-related control | Number or proportion of cases |

| Study[1] | Study type | WHO Region | Geographical setting | Region protected by travel-related measure | Region restricted by travel-related measure | Disease | Intervention category | Outcome type |
|---|---|---|---|---|---|---|---|---|
| | | | | | | | measures | |
| Yu 2012 | Inferential | WPR | Non-island state | China | All other countries | Influenza (H1N1 2009 pandemic) | Entry/exit screening | Number or proportion of cases |
| Yuan 2020 (pre-print) | Inferential | WPR | Non-island state | Other regions of China and countries | Wuhan | COVID-19 | Multiple travel-related control measures | Temporal development of epidemic |
| Yuan 2020a (pre-print) | Inferential | WPR | Non-island state | China | Wuhan | COVID-19 | Multiple travel-related control measures | Number or proportion of cases |
| Zhang 2012 | Descriptive | WPR | Non-island state | Beijing | All other countries | Influenza (H1N1 2009 pandemic) | Entry/exit screening | Number or proportion of screened identified as cases |
| Zhang 2014 | Inferential | n.a. | Hypothetical | Hypothetical | Hypothetical | Influenza (pandemic) | Entry/exit screening | Number or proportion of cases; Temporal development of epidemic |
| Zhang 2019 | Inferential | n.a. | Hypothetical | Hypothetical | Hypothetical | Influenza (H1N1 2009 pandemic) | Entry/exit screening; Reductions in human mobility | Temporal development of epidemic |
| Zhang 2020 (pre-print) | Inferential | WPR | Non-island state | Other regions of China | Wuhan and other cities in Hubei | COVID-19 | Multiple travel-related control measures | Number or proportion of cases |
| Zhang 2020a (pre-print) | Inferential | WPR | Non-island state | Other regions of China | Wuhan and other cities in Hubei | COVID-19 | Multiple travel-related control measures | Number or proportion of cases; Reproduction number |
| Zhao 2020 | Inferential | WPR | Non-island state | Regions of China | Regions of China | COVID-19 | Multiple travel-related control measures | Number or proportion of cases; Reproduction number; Temporal |

| Study[1] | Study type | WHO Region | Geographical setting | Region protected by travel-related measure | Region restricted by travel-related measure | Disease | Intervention category | Outcome type |
|---|---|---|---|---|---|---|---|---|
| | | | | | | | | development of epidemic |
| Zhou 2020 (pre-print) | Inferential | WPR | Non-island state | Other regions of China | Wuhan and other cities in Hubei | COVID-19 | Multiple travel-related control measures | Number or proportion of cases; Number or proportion of deaths |
| Zlojutro 2018 | Inferential | AMR | Non-island state | USA | All other countries | Influenza (H1N1 2009 pandemic) | Entry/exit screening | Number or proportion of cases; Cost; Other |

peer review only

## COVID-19 and other relevant respiratory infectious diseases

Included studies assessed the impact of interventions aiming to prevent or slow the transmission of COVID-19 and other infectious respiratory diseases. We identified a large number of studies focusing on COVID-19 (n=59); we also found studies focusing on SARS (n=11), MERS (n=1) and various strains of influenza (n=39). Other studies looked at multiple infectious respiratory diseases (n=5) or a hypothetical infectious disease with COVID-19-relevant properties (n=7).

Figure 2 illustrates how the number of publications concerned with each disease has developed over time, and explores where interventions have been implemented. As expected, there has been a rapid burst of research related to COVID-19 travel restrictions in 2020 (panel A), currently consisting mostly of non-peer-reviewed pre-prints. Research on SARS, MERS and influenza travel restrictions is more spread over time, but also clusters around specific outbreaks (e.g. SARS 2003; H1N1 influenza 2009) (panel B).

## Intervention categories and interventions

The 122 included studies assessed the impact of a wide range of travel-related control measures. We classified these according to broad intervention categories, including border closures (n=3), entry/exit screening (n=31), travel-related quarantine (n=6), travel bans, such as suspension of international flights (n=8), and travel restrictions (n=25).

For travel restrictions, a few studies described imposing restrictions in relation to the mode of travel (e.g. restricting air, land, or maritime travel); most used the term “travel restrictions” without providing any specification. However, most of the studies in this category were modeling studies, which commonly simulated “travel restrictions” as different percentage reductions in the travel volume (e.g. 50% and 90%).

We identified a relatively large number of studies assessing the impact of a bundle of different travel-related control measures (e.g. entry/exit screening and quarantining all arriving passengers) (n=49). More than half of these (n=29) assessed the impact of the lockdown of Wuhan (n=29), combining several travel-related measures. We classified all of these studies as assessing *multiple travel-related control measures* (see online supplementary file S6), as it was impossible to identify the specific measures assessed due to lack of reporting.

Figure 3 visualizes the body of evidence according to the respective disease, intervention implemented, as well as region in which an intervention was implemented. It becomes clear that the majority of the evidence assesses multiple travel-related control measures to delay or limit the progression of COVID-19. The second largest block of evidence emerges from studies focusing on various influenza strains and the impact of travel restrictions and entry/exit screening.

A critical aspect in relation to the likely impact of travel-related control measures is the timing of implementation. Figure 4 (panel A) visualizes during which phase of an epidemic or pandemic different types of interventions addressed by included studies were implemented. With respect to the timing of implementation we distinguish the following:

- Early phase: Interventions are implemented at a time when there are either no or only singular detected/notified cases (i.e. all cases are detected and quarantined). During this phase, imported cases represent the main source of infections.



For peer review only - http://bmjopen.bmj.com/site/about/guidelines.xhtml

- Local transmission phase: Interventions are implemented during more or less widespread human-to-human transmission of the disease. During this phase local transmission and imported cases represent sources of infections.
- Post-peak phase: Interventions are implemented during/after successful containment of an initial outbreak/epidemic/pandemic with the possibility of recurrence. During this phase imported cases may again represent a major source of infections.
- Unclear phase: The phase of the outbreak/epidemic/pandemic is not reported or is not directly relevant for implementation of the intervention.

Figure 4 (panel A) shows, for example, that many interventions, especially those comprising multiple measures, are implemented when local transmission of the disease has already been established. Travel restrictions are often employed in the early phase of an epidemic or pandemic while entry/exit screening measures tend to be implemented both in the early stages, as well as throughout the phases of an epidemic or pandemic. Further details on the specific travel-related control measures reported in each included study and their phase of implementation is presented in the online supplementary file S6.

## Outcome categories and outcomes

We considered studies assessing five broad categories of outcomes: infectious disease outcomes, screening outcomes, other health outcomes, economic outcomes, and social outcomes. Identified studies, however, largely assessed infectious disease (n=98) and/or screening outcomes (n=25). We identified no studies concerned with other health outcomes, and very few studies assessing economic (n=5) or social outcomes (n=1).

Infectious disease outcomes included several types of outcomes related to disease timing and transmission, including the number or proportion of cases, the number or proportion of deaths, the reproduction number, the probability of an epidemic, demand for healthcare resources, and the temporal development of the epidemic. Studies assessed various specific outcomes under these broader types; for example, ‘time to epidemic peak’ and ‘delay of epidemic’ both belong to the outcome type ‘temporal development of the epidemic’. Screening outcomes all comprised some form of measuring the number and/or proportion of high risk persons and/or cases detected. Economic outcomes covered costs and industry impact, and social outcomes examined the acceptability of travel-related control measures. These outcome types as assessed in each study are listed in Table 1. A comprehensive list of the specific outcomes reported for each category and type of outcome and how often these were utilized across the evidence map can be found in online supplementary file S7.

Figure 4 (panel B) illustrates which intervention categories were assessed by different outcome categories. Infectious disease outcomes such as the number or proportion of cases, the temporal development of an epidemic, and the reproduction number were well-represented across all intervention types. Conversely, screening outcomes were assessed primarily with respect to entry/exit screening. The lack of studies assessing economic and social outcomes is also clearly visible.

## Study types

We included all studies assessing the quantitative impact of travel-related control measures on infectious disease, screening, economic and social outcomes. As a consequence of this broad scope,



For peer review only - http://bmjopen.bmj.com/site/about/guidelines.xhtml

included studies employed a range of vastly different approaches drawing from the fields of infectious disease research, epidemiology, economics, biology, and mathematics, among others. We categorized each study as being either inferential or descriptive. Most of the studies we found were inferential in nature (n=103), aiming to retrospectively calculate or prospectively forecast the impact of one or multiple travel-related control measures on outcomes. The remaining studies were descriptive in nature (n=19), aiming to describe the impact of control measures through summary statistics and/or graphics.

Within these broad categories, however, included studies varied greatly with regard to the specific approach taken. Inferential studies applied numerous modeling and epidemiological techniques; compartmental models, such as SIR models (S: susceptible, I: infectious, R: recovered) or SIR model derivatives, were common. Several studies also applied spatial models to explore how disease transmission moves geographically. Epidemiological time-series models, as well as other epidemiological modeling and testing strategies, were also common. Descriptive studies comprised primarily observational studies and graphical summary studies, both of which measured and reported descriptive summary statistics related to intervention impact. The types of studies illustrated here are not exhaustive, and the methodological boundaries between the approaches employed are sometimes blurry. In fact, several studies apply multiple techniques, combining, for example, compartmental and spatial models or compartmental and time-series models. Online supplementary file S8 provides a more detailed overview of the study types included, along with examples from the included studies.

## Discussion

### Summary of findings

This evidence map provides a comprehensive overview of travel-related control measures available for the control of SARS-CoV-2/COVID-19.

Most of the included studies used infectious disease or epidemiological modeling methods to examine the impact of travel-related control measures on the current COVID-19 pandemic. We also identified studies on SARS-CoV-1/SARS and influenza, mostly undertaken in the context of previous epidemics/pandemics. We found very few studies addressing MERS-CoV/MERS. The identified studies assessing travel-related control measures in the context of SARS-CoV-2/COVID-19, were mostly pre-print publications which have not yet undergone peer review and are often characterized by poor reporting and conduct (12).

Studies were undertaken across the globe. The geographical region most represented was WPR, driven in part by a number of studies focusing on the Hubei region of China and in part by studies across the region during the previous SARS-CoV-1/SARS outbreak. AFR and EUR were the least represented; we did not identify any studies from EMR. Most studies operated at the level of countries and reported little about specific settings of interest (e.g. a named airport, a specific country border) or the broader implementation context (e.g. usual border arrangements when control measures are not in place).

Travel-related control measures can be classified as (i) border closure, (ii) travel bans, (iii) travel restrictions, (iv) entry/exit screening, (v) travel-related quarantine, and (vi) multiple travel-related



For peer review only - http://bmjopen.bmj.com/site/about/guidelines.xhtml

control measures. Studies were identified and mapped for all of these categories. We identified a relatively large body of evidence on entry/exit screening, as well as on travel restrictions. The latter also included sub-national measures, e.g. city-to-city travel restrictions across broader regions, making a clear distinction from general social distancing measures challenging. We also found many studies examining the impact of bundles of travel-related control measures. Interventions are often poorly described, both in relation to the measure itself (e.g. border closure) and in relation to how the measure is implemented or enforced (e.g. border patrols, fines, exceptions). Moreover, travel-related control measures rarely happen in a vacuum – the closer to real-life the intervention (and the study), the more co-interventions tend to be involved. This makes it very difficult to assess the unique impact of a specific measure.

The impact of travel-related control measures in controlling geographical spread and overall disease transmission likely varies between early, local transmission and post peak phases of an outbreak/epidemic/pandemic, yet reporting of the timing of implementation tends to be poor. Most studies focused on the early and local transmission phases; few studies were concerned with the post-peak phase.

The identified studies almost exclusively examined infectious disease and screening outcomes. Surprisingly, we did not identify any study reporting on other health outcomes, such as implications for the physical and psychosocial health of stranded travelers, of those unable to visit family members, of regular commuters unable to reach their workplace, of individuals quarantined, of people unable to obtain medical treatment and other collateral damage (e.g. suspended immunization programs and impacts on food supply due to limited air, maritime and land travel of either people or goods). Only three included studies were concerned with economic and social outcomes.

The bulk of the evidence derives from modeling studies. In an acute outbreak situation, the time and resources that can be dedicated to conducting timely, empirical research are limited. Modeling studies vary greatly in type (e.g. SIR-like models vs. time series models), and in the underlying assumptions regarding the disease, interventions or regions/settings. Additionally, many studies do not fall into clean categories, and a single study may combine compartmental infectious disease models with epidemiological spatial models. They also vary with respect to whether and how they have been validated through real-life applications.

## Gaps in the current evidence base

The evidence map identified several gaps in the evidence base related to travel-related control measures that could inform future research as well as evidence synthesis. First, some regions were underrepresented, specifically studies conducted or simulated for the countries in the African, Eastern Mediterranean and European Regions were lacking. Second, the most commonly reported travel-related control measures were entry/exit screening and restrictions, which in the context of modelling studies were simulated as different levels of travel reductions. Thus the evidence base regarding more stringent measures, such as travel bans and complete border closures remains sparse. Third, our evidence map identified lack of consideration of the impact of these measures on broader health outcomes, such as physical and mental health, as well as social and economic outcomes, such as cost and burden on communities and socio-economic inequalities. Finally, the key



For peer review only - http://bmjopen.bmj.com/site/about/guidelines.xhtml

gap relates to the lack of empirical studies assessing the impact of travel-related control measures, including experimental and quasi-experimental approaches.

## Methodological limitations of this study

This evidence map was put together over ten days, and the process is thus characterized by several limitations.

While we conducted searches in three major databases and two COVID-specific databases, these were mostly health-centric. We only searched Web of Science – comprising Science Citation Index Expanded (1900-present), Social Sciences Citation Index (1900-present) and Emerging Sources Citation Index (2015-present) – to identify social and economic studies. As we identified very few economic and social outcomes, it is likely that there is another body of evidence to be located with more focused searches in specific economic and social science databases. In addition, we did not search for grey literature sources. Furthermore, since COVID-19 initially started in Wuhan, China, many studies are published on the topic in Chinese journals and databases. Because of the language barrier, we did not consider these sources.

The unspecific and inconsistent reporting of primary studies with regard to interventions, especially when a package of control measures was investigated, sometimes made inclusion/exclusion decisions difficult. While we developed and calibrated screening guidance to ensure consistency among reviewers, it is nevertheless possible that we excluded some travel-related control measures that were not explicitly described as such. Also, we did not undertake double-screening of all studies but only re-assessed a sub-set of studies excluded at the full-text screening stage. In general, however, we applied a very conservative approach to title/abstract, as well as full-text screening, where any uncertainties associated with a study were marked for further checking by a second reviewer and/or for discussion among the whole review team.

We developed and calibrated data extraction guidance to be used consistently by reviewers. Nevertheless, we had to refine some categories post hoc. We also had a large number of reviewers extracting data, which created heterogeneity in the dataset. To address this, a second reviewer double-checked all extracted information for each of the main domains, i.e. all information regarding interventions, outcomes and study design. Overall, high-quality data extraction was limited by poor reporting of the travel-related control measures and the specific contexts in which they are implemented in primary studies (13).

Finally, we did not include travel warning or travel advice in the evidence map, which limits the scope of this map. While these may be classified as travel-related control measures (13), their inclusion would have likely broadened our search strategy and prolonged the timeline of its development. In general, lack of intervention specification in the included studies makes it challenging to draw clear categories of interventions without potential overlap (e.g. drawing clear distinction between a travel ban vs. a travel restriction, when these are used only as descriptors in the studies without further specification).



For peer review only - http://bmjopen.bmj.com/site/about/guidelines.xhtml

## Implications for moving forward

An evidence map is not designed to assess the effectiveness of interventions, in this case the effectiveness of travel-related control measures in controlling infectious disease spread. The present evidence map sheds light on the variety of evidence available with regards to the quantifiable impacts of travel-related control measures, the outcomes utilized, as well as the study types employed. It thus represents a stepping stone towards a systematic review on the effectiveness of all or a subset of travel-related control measures.

Given our health-centric searches, we feel confident that we have identified most of the available body of evidence regarding the quantifiable impacts of travel-related control measures on health. In terms of conducting a systematic review of effectiveness it would, however, be advisable to undertake additional forward-backward citation searches and/or similar studies searches with included studies. We identified a number of challenges related to the full analysis of this evidence base. These include: (i) classifying interventions in an appropriate and consistent manner; (ii) capturing the details of interventions (e.g. components, timing, implementation characteristics) and co-interventions through an analysis of linked sources of evidence; (iii) assessing the quality and usefulness of different types of studies ranging from simple observational studies to complex modeling studies; (iv) quantitatively synthesizing this extremely heterogeneous evidence base; and (v) dealing with the large number of pre-print studies and their varying quality. Moving forward with a full analysis, we suggest reviewers plan ahead and develop strategies on how these challenges can be adequately managed. For example, this might entail consideration of external sources of evidence beyond scientific databases, such as governmental websites on the implementation of different travel-related control measures (e.g., their timing and duration) and other measures in local contexts. To contain the COVID-19 pandemic, governments have employed a range of public health measures often in a bundle, which challenges assessment of the effectiveness of any single measure, such as a travel ban. It would therefore be important to explicitly document and, where possible, assess various combinations of measures implemented - including those related to travel - in future research.

Importantly, decisions to maintain or stop travel-related control measures are determined by a range of factors beyond effectiveness, including legal and human rights aspects, as well as considerations of broader health, economic and social implications, as well as socio-cultural and political acceptability. Gathering evidence about these broader factors in a systematic manner was beyond the scope of the current evidence map, however such aspects might be important to inform decision-making. Future evidence synthesis would therefore require a different scope and a broader search strategy, notably encompassing searches in economics and social science databases, as well as multidisciplinary databases (e.g. EconLit, PsycINFO and Scopus).

Contributors: JB, AM, ER, and OH defined the study scope and developed the study protocol with significant intellectual input from all review authors. JB and AM coordinated the entire study process. IK designed the search strategy and conducted all the searches. JB, AM, RB, MC, KG, LMP, PP, KS, BS, JMS, SV and ER conducted data screening and extraction. JB, AM, JMS, LMP and PP did data mapping. JB, AM, and ER prepared the first draft of the manuscript, which was further critically examined by all the authors and revised based on their feedback. All review authors reviewed and approved the final draft.

Competing interests: The authors declare they have no competing interests.


For peer review only - http://bmjopen.bmj.com/site/about/guidelines.xhtml

Funding: The conduct of this evidence map was funded by the World Health Organization (WHO; grand/award number: N/A).

Disclaimer: This study was commissioned and paid for by the World Health Organization (WHO). Copyright of the original work, a report submitted to WHO, belongs to WHO. This article was developed based on the WHO report and the authors have been given permission to publish. The authors alone are responsible for the views expressed in this publication and they do not necessarily represent the views, decisions or policies of WHO.

Data sharing statement: No original data were generated for this study. All studies included in the evidence map are presented in the supplementary file.

Patient consent: Not applicable.

Provenance and peer review: Commissioned by the World Health Organization (WHO) and externally peer reviewed.

Acknowledgements: We are grateful to Susan L Norris and Carmen Dolea for their guidance in scoping this evidence map.

For peer review only



For peer review only - http://bmjopen.bmj.com/site/about/guidelines.xhtml

## References

1. WHO. Novel Coronavirus (2019-nCoV) Situation Report - 1. World Health Organization; 2020.

2. WHO. Novel Coronavirus (2019-nCoV) Situation Report - 51. World Health Organization; 2020.

3. WHO. Weekly update on COVID-19, April 8–15, 2020. Health Emergencies Programme. Geneva: World Health Organization; 2020.

4. Mateus ALP, Otete HE, Beck CR, Dolan GP, Nguyen-Van-Tam JS. Effectiveness of travel restrictions in the rapid containment of human influenza: a systematic review. Bull WHO. 2014;92(12):868-80D.

5. Ryu S, Gao H, Wong JY, Shiu EYC, Xiao J, Fong MW, et al. Nonpharmaceutical Measures for Pandemic Influenza in Nonhealthcare Settings-International Travel-Related Measures. Emerging infectious diseases. 2020;26(5):961-6.

6. WHO. Non-pharmaceutical public health measures for mitigating the risk and impact of epidemic and pandemic influenza. Licence: CC BY-NC-SA 3.0 IGO.: Licence: CC BY-NC-SA 3.0 IGO.; 2019.

7. Mouchtouri VA, Christoforidou EP, An der Heiden M, Menel Lemos C, Fanos M, Rexroth U, et al. Exit and Entry Screening Practices for Infectious Diseases among Travelers at Points of Entry: Looking for Evidence on Public Health Impact. International journal of environmental research and public health. 2019;16(23):4638-.

8. Pasquini-Descomps H, Brender N, Maradan D. Value for Money in H1N1 Influenza: A Systematic Review of the Cost-Effectiveness of Pandemic Interventions. Value Health. 2017;20(6):819-27.

9. Perez Velasco R, Praditsitthikorn N, Wichmann K, Mohara A, Kotirum S, Tantivess S, et al. Systematic review of economic evaluations of preparedness strategies and interventions against influenza pandemics. PLoS ONE. 2012;7(2):e30333.

10. Errett NA, Sauer LM, Rutkow L. An integrative review of the limited evidence on international travel bans as an emerging infectious disease disaster control measure. Journal of emergency management (Weston, Mass). 2020;18(1):7-14.

11. Ouzzani M, Hammady H, Fedorowicz Z, Elmagarmid A. Rayyan-a web and mobile app for systematic reviews. Syst Rev. 2016;5(1):210.

12. Glaszio P. Waste in covid-19 research. BMJ. 2020;369:m1847.

13. Lee K, Worsnop CZ, Grepin KA, Kamradt-Scott A. Global coordination on cross-border travel and trade measures crucial to COVID-19 response. Lancet. 2020.

For peer review only

# Figures

**Figure 1.** Flowchart of studies identified and included during different stages of searching and screening

**Figure 2.** Illustration of the number of studies published over time; the top panel (2002-2020) shows studies focused on SARS, MERS, influenza and hypothetical disease with COVID-19 relevant properties, while the bottom panel (2020) shows studies focused on COVID-19. The specific disease is indicated by the single letter within the circle. Additionally, the color represents the WHO world region

**Figure 3.** Overview of the body of evidence, showing the frequency of studies investigating the specific diseases (left column), interventions (middle column), and the WHO world regions (right column)

**Figure 4.** Bubble plots illustrating in included studies (A) during which phase of an epidemic or pandemic different types of interventions were implemented, and (B) which intervention categories were assessed against different types of outcomes

For peer review only



For peer review only - http://bmjopen.bmj.com/site/about/guidelines.xhtml

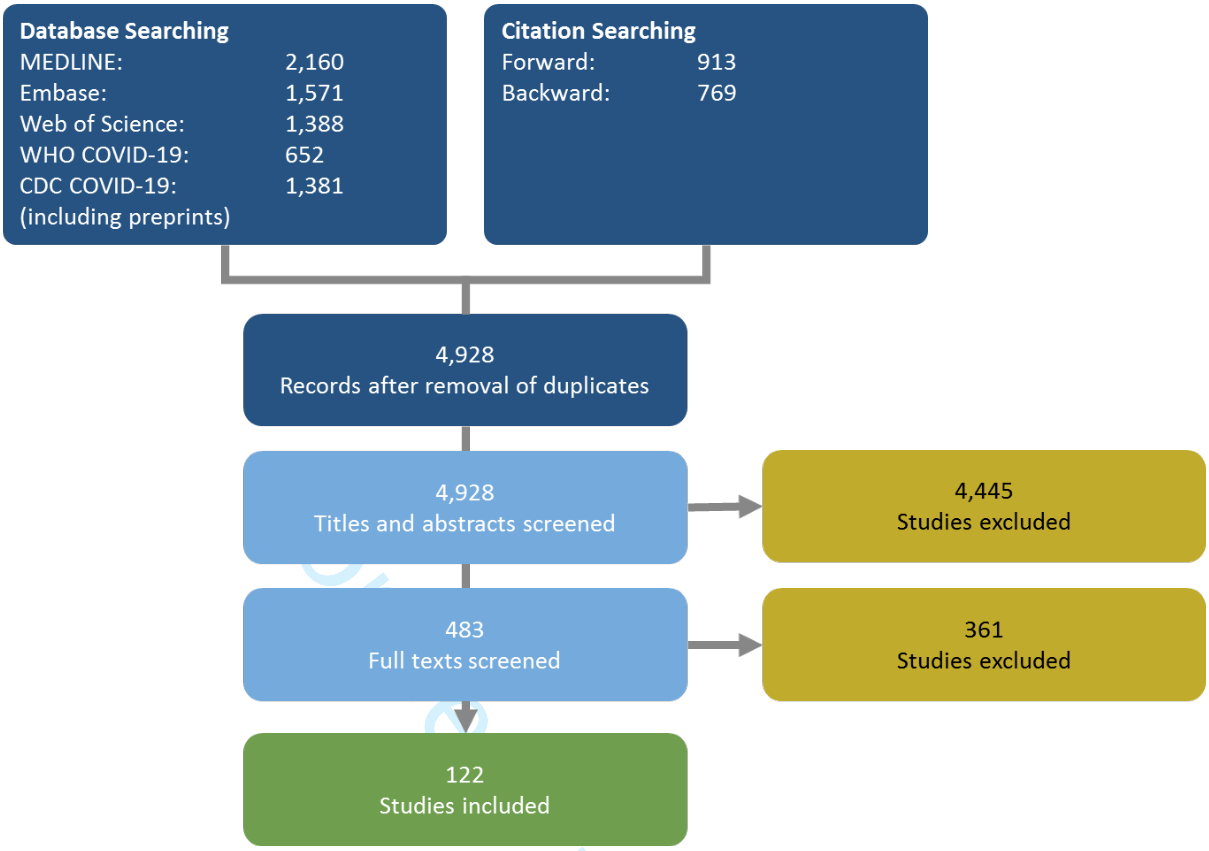
Database Searching
MEDLINE: 2,160
Embase: 1,571
Web of Science: 1,388
WHO COVID-19: 652
CDC COVID-19: 1,381
(including preprints)
Citation Searching
Forward: 913
Backward: 769
4,928
Records after removal of duplicates
4,928
Titles and abstracts screened
4,445
Studies excluded
483
Full texts screened
361
Studies excluded
122
Studies included

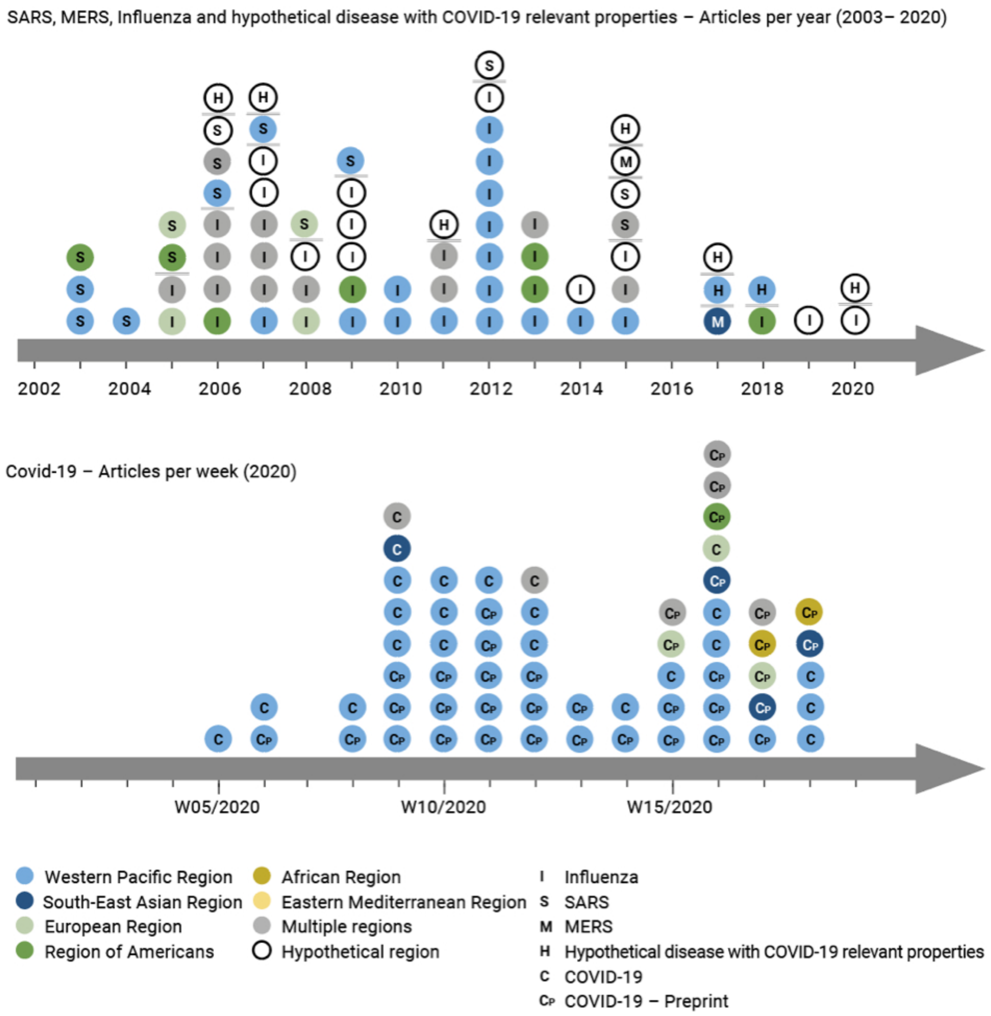


Figure 2. Illustration of the number of studies published over time; the top panel (2002-2020) shows studies focused on SARS, MERS, influenza and hypothetical disease with COVID-19 relevant properties, while the bottom panel (2020) shows studies focused on COVID-19. The specific disease is indicated by the single letter within the circle. Additionally, the color represents the WHO world region

338x345mm (72 x 72 DPI)

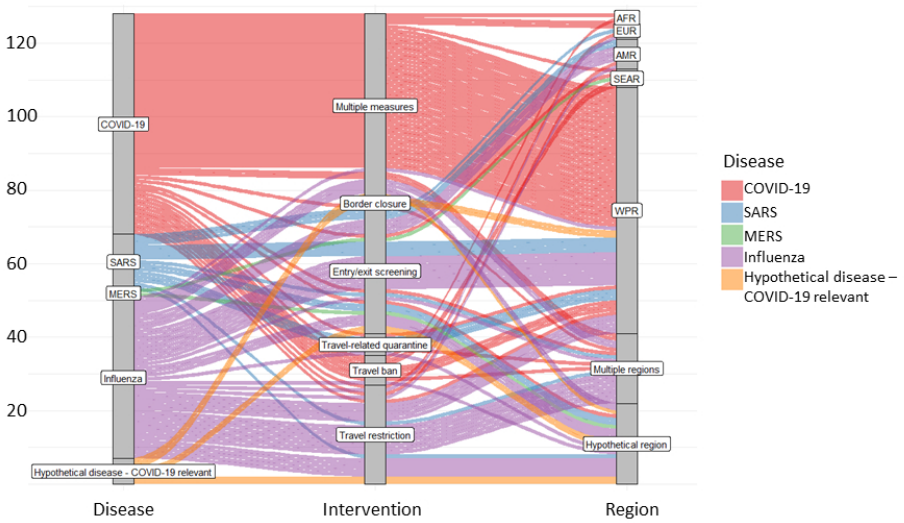


Figure 3. Overview of the body of evidence, showing the frequency of studies investigating the specific diseases (left column), interventions (middle column), and the WHO world regions (right column)

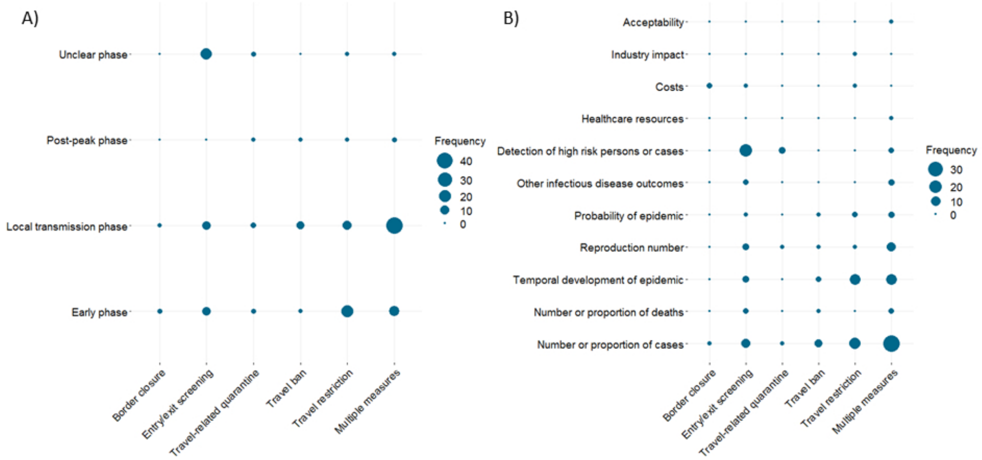


Figure 4. Bubble plots illustrating in included studies (A) during which phase of an epidemic or pandemic different types of interventions were implemented, and (B) which intervention categories were assessed against different types of outcomes

## Supplementary file

## S1: Preferred Reporting Items for Systematic reviews and Meta-Analyses extension for Scoping Reviews (PRISMA-ScR) Checklist

| SECTION | ITEM | PRISMA-ScR CHECKLIST ITEM | REPORTED ON PAGE # |
|---|---|---|---|
| **TITLE** | | | |
| Title | 1 | Identify the report as a scoping review. | |
| **ABSTRACT** | | | |
| Structured summary | 2 | Provide a structured summary that includes (as applicable): background, objectives, eligibility criteria, sources of evidence, charting methods, results, and conclusions that relate to the review questions and objectives. | |
| **INTRODUCTION** | | | |
| Rationale | 3 | Describe the rationale for the review in the context of what is already known. Explain why the review questions/objectives lend themselves to a scoping review approach. | |
| Objectives | 4 | Provide an explicit statement of the questions and objectives being addressed with reference to their key elements (e.g., population or participants, concepts, and context) or other relevant key elements used to conceptualize the review questions and/or objectives. | |
| **METHODS** | | | |
| Protocol and registration | 5 | Indicate whether a review protocol exists; state if and where it can be accessed (e.g., a Web address); and if available, provide registration information, including the registration number. | |
| Eligibility criteria | 6 | Specify characteristics of the sources of evidence used as eligibility criteria (e.g., years considered, language, and publication status), and provide a rationale. | |
| Information sources* | 7 | Describe all information sources in the search (e.g., databases with dates of coverage and contact with authors to identify additional sources), as well as the date the most recent search was executed. | |
| Search | 8 | Present the full electronic search strategy for at least 1 database, including any limits used, such that it could be repeated. | |
| Selection of sources of evidence† | 9 | State the process for selecting sources of evidence (i.e., screening and eligibility) included in the scoping review. | |
| Data charting process‡ | 10 | Describe the methods of charting data from the included sources of evidence (e.g., calibrated forms or forms that have been tested by the team before their use, and whether data charting was done independently or in duplicate) and any processes for obtaining and confirming data from investigators. | |
| Data items | 11 | List and define all variables for which data were sought and any assumptions and simplifications made. | |
| Critical appraisal of individual sources of evidence§ | 12 | If done, provide a rationale for conducting a critical appraisal of included sources of evidence; describe the methods used and how this information was used in any data synthesis (if appropriate). | |
| Synthesis of results | 13 | Describe the methods of handling and summarizing the data that were charted. | |

St. Michael's
Inspired Care.
Inspiring Science.



For peer review only - http://bmjopen.bmj.com/site/about/guidelines.xhtml

| SECTION | ITEM | PRISMA-ScR CHECKLIST ITEM | REPORTED ON PAGE # |
|---|---|---|---|
| **RESULTS** | | | |
| Selection of sources of evidence | 14 | Give numbers of sources of evidence screened, assessed for eligibility, and included in the review, with reasons for exclusions at each stage, ideally using a flow diagram. | |
| Characteristics of sources of evidence | 15 | For each source of evidence, present characteristics for which data were charted and provide the citations. | |
| Critical appraisal within sources of evidence | 16 | If done, present data on critical appraisal of included sources of evidence (see item 12). | |
| Results of individual sources of evidence | 17 | For each included source of evidence, present the relevant data that were charted that relate to the review questions and objectives. | |
| Synthesis of results | 18 | Summarize and/or present the charting results as they relate to the review questions and objectives. | |
| **DISCUSSION** | | | |
| Summary of evidence | 19 | Summarize the main results (including an overview of concepts, themes, and types of evidence available), link to the review questions and objectives, and consider the relevance to key groups. | |
| Limitations | 20 | Discuss the limitations of the scoping review process. | |
| Conclusions | 21 | Provide a general interpretation of the results with respect to the review questions and objectives, as well as potential implications and/or next steps. | |
| **FUNDING** | | | |
| Funding | 22 | Describe sources of funding for the included sources of evidence, as well as sources of funding for the scoping review. Describe the role of the funders of the scoping review. | |

JBI = Joanna Briggs Institute; PRISMA-ScR = Preferred Reporting Items for Systematic reviews and Meta-Analyses extension for Scoping Reviews.
* Where *sources of evidence* (see second footnote) are compiled from, such as bibliographic databases, social media platforms, and Web sites.
† A more inclusive/heterogeneous term used to account for the different types of evidence or data sources (e.g., quantitative and/or qualitative research, expert opinion, and policy documents) that may be eligible in a scoping review as opposed to only studies. This is not to be confused with *information sources* (see first footnote).
‡ The frameworks by Arksey and O'Malley (6) and Levac and colleagues (7) and the JBI guidance (4, 5) refer to the process of data extraction in a scoping review as data charting.
§ The process of systematically examining research evidence to assess its validity, results, and relevance before using it to inform a decision. This term is used for items 12 and 19 instead of "risk of bias" (which is more applicable to systematic reviews of interventions) to include and acknowledge the various sources of evidence that may be used in a scoping review (e.g., quantitative and/or qualitative research, expert opinion, and policy document).

*From:* Tricco AC, Lillie E, Zarin W, O'Brien KK, Colquhoun H, Levac D, et al. PRISMA Extension for Scoping Reviews (PRISMAScR): Checklist and Explanation. Ann Intern Med. 2018;169:467–473. doi: 10.7326/M18-0850.


For peer review only - http://bmjopen.bmj.com/site/about/guidelines.xhtml

**S2:** MEDLINE search strategy and results

| Ovid MEDLINE(R) ALL 1946 to May 01, 2020 | | |
|---|---|---|
| **#** | **Searches** | **Results** |
| 1 | exp Coronavirus/ | 12994 |
| 2 | exp Coronavirus Infections/ | 11480 |
| 3 | (coronavir* or coronovir*).ti,ab,kf. | 14864 |
| 4 | ((corona* or corono*) adj1 (virus* or viral* or virinae*)).ti,ab,kf. | 578 |
| 5 | (ncov or n-cov or 2019nCoV or nCoV2019 or CO?VID-19 or CO?VID19 or WN-CoV or WNCoV or HCoV-19 or HCoV19 or 2019 novel* or SARS-CoV-2 or SARSCoV-2 or SARSCoV2 or SARS-CoV2 or SARSCov19 or SARS-Cov19 or SARSCov-19 or SARS-Cov-19 or Ncovor or Ncorona* or Ncorono* or NcovWuhan* or NcovHubei* or NcovChina* or NcovChinese* or SARS2 or SARS-2 or SARScoron?virus2 or SARS-coron?virus-2 or SARScoron?virus 2 or SARS coron?virus2).ti,ab,kf. | 7647 |
| 6 | (Middle East Respiratory Syndrome or MERS or Severe Acute Respiratory Syndrome or SARS).ti,ab,kf. | 15761 |
| 7 | Influenza, Human/ | 48540 |
| 8 | exp influenzavirus a/ or exp influenzavirus b/ or exp influenzavirus c/ | 44601 |
| 9 | (influenza* not (h?em?phil* influenza* or "h influenza*")).ti,ab,kf. | 95326 |
| 10 | (flu or H1N1 or H2N2 or H3N2 or H1N12 or H5N1).ti,ab,kf. | 36447 |
| 11 | or/1-10 | 140838 |
| 12 | travel/ or air travel/ | 25098 |
| 13 | quarantine/ | 2164 |
| 14 | (travel* or border?).ti. | 25986 |
| 15 | (travel* adj3 (restrict* or reduc* or control* or limit* or lockdown? or ban*)).ab,kf. | 1461 |
| 16 | (travel adj4 (measure? or intervention? or NPI?)).ab,kf. | 401 |
| 17 | (border? adj3 (clos* or restrict* or control* or measure?)).ab,kf. | 1014 |
| 18 | ((screen* or surveil*) adj4 (traveller? or entry or exit or border? or airport?)).ti,ab,kf. | 1124 |
| 19 | visa?.ti,ab,kf. | 2044 |
| 20 | quarantin*.ti,ab,kf. | 4807 |
| 21 | (isolat* adj6 (exposed or suspected or travel* or airport? or border?)).ti,ab,kf. | 7659 |
| 22 | or/12-21 | 59213 |
| 23 | 11 and 22 | 2405 |
| 24 | limit 23 to "humans only (removes records about animals)" | 2247 |

| | | |
|---|---|---|
| 25 | 24 and (armenian or english or french or german or italian or russian).lg. | 2160 |

For peer review only



For peer review only - http://bmjopen.bmj.com/site/about/guidelines.xhtml

**S3:** List of (systematic) reviews used for forward and backward citation tracking


1. Ahmad A, Krumkamp R, Reintjes R. Controlling SARS: a review on China's response compared with other SARS-affected countries. Tropical Medicine & International Health. 2009;14(s1):36-45.

2. Aledort JE, Lurie N, Wasserman J, Bozzette SA. Non-pharmaceutical public health interventions for pandemic influenza: an evaluation of the evidence base. BMC Public Health. 2007;7(1):208-.

3. Bitar D, Goubar A, Desenclos JC. International travels and fever screening during epidemics: a literature review on the effectiveness and potential use of non-contact infrared thermometers. Eurosurveillance. 2009;14(6):19115.

4. Bradt DA, Drummond CM. Avian influenza pandemic threat and health systems response. Emergency Medicine Australasia. 2006;18(5-6):430-43.

5. Cowling BJ, Lau LLH, Wu P, Wong HWC, Fang VJ, Riley S, et al. Entry screening to delay local transmission of 2009 pandemic influenza A (H1N1). BMC Infectious Diseases. 2010;10(1):82-.

6. Errett NA, Sauer LM, Rutkow L. An integrative review of the limited evidence on nternational travel bans as an emerging infectious disease disaster control measure. Journal of emergency management (Weston, Mass). 2020;18(1):7-14.

7. Gössling S, Scott D, Hall CM. Pandemics, tourism and global change: a rapid assessment of COVID-19. Journal of Sustainable Tourism. 2020:1-20.

8. Group WHOW, Bell D, Nicoll A, Fukuda K, Horby P, Monto A, et al. Non-pharmaceutical interventions for pandemic influenza, national and community measures. Emerging infectious diseases. 2006;12(1):88-94.

9. Huizer YL, Swaan CM, Leitmeyer KC, Timen A. Usefulness and applicability of infectious disease control measures in air travel: A review. Travel Medicine and Infectious Disease. 2015;13(1):19-30.

10. Inglesby TV, Nuzzo JB, O'Toole T, Henderson DA. Disease Mitigation Measures in the Control of Pandemic Influenza. Biosecurity and Bioterrorism: Biodefense Strategy, Practice, and Science. 2006;4(4):366-75.

11. Jefferson T, Del Mar CB, Dooley L, Ferroni E, Al-Ansary LA, Bawazeer GA, et al. Physical interventions to interrupt or reduce the spread of respiratory viruses. Cochrane Database of Systematic Reviews. 2011(7).

12. Juneau C-E, Pueyo T, Bell M, Gee G, Potvin L. Evidence-based, cost-effective interventions to suppress the COVID-19 pandemic: a rapid systematic review. medRxiv. 2020:2020.04.20.20054726-2020.04.20.




For peer review only - http://bmjopen.bmj.com/site/about/guidelines.xhtml

13. Kelly HA, Priest PC, Mercer GN, Dowse GK. We should not be complacent about our population-based public health response to the first influenza pandemic of the 21st century. BMC public health. 2011;11:78-.

14. Lee VJ, Lye DC, Wilder-Smith A. Combination strategies for pandemic influenza response - a systematic review of mathematical modeling studies. BMC Medicine. 2009;7(1):76-.

15. Lugnér AK, Postma MJ. Mitigation of pandemic influenza: review of cost–effectiveness studies. Expert Review of Pharmacoeconomics & Outcomes Research. 2009;9(6):547-58.

16. Mateus ALP, Otete HE, Beck CR, Dolan GP, Nguyen-Van-Tam JS. Effectiveness of travel restrictions in the rapid containment of human influenza: a systematic review. Bulletin of the World Health Organization. 2014;92(12):868-80D.

17. Mouchtouri VA, Christoforidou EP, An der Heiden M, Menel Lemos C, Fanos M, Rexroth U, et al. Exit and Entry Screening Practices for Infectious Diseases among Travelers at Points of Entry: Looking for Evidence on Public Health Impact. International journal of environmental research and public health. 2019;16(23):4638-.

18. Nicola M, Alsafi Z, Sohrabi C, Kerwan A, Al-Jabir A, Iosifidis C, et al. The Socio-Economic Implications of the Coronavirus and COVID-19 Pandemic: A Review. International journal of surgery (London, England). 2020:S1743-9191(20)30316-2.

19. Nussbaumer-Streit B, Mayr V, Dobrescu AI, Chapman A, Persad E, Klerings I, et al. Quarantine alone or in combination with other public health measures to control COVID-19: a rapid review. Cochrane Database of Systematic Reviews. 2020(4).

20. Paudel S, Dangal G, Chalise A, Bhandari TR, Dangal O. The Coronavirus Pandemic: What Does the Evidence Show? Journal of Nepal Health Research Council. 2020;18(1):1-9.

21. Rashid H, Ridda I, King C, Begun M, Tekin H, Wood JG, et al. Evidence compendium and advice on social distancing and other related measures for response to an influenza pandemic. Paediatric Respiratory Reviews. 2015;16(2):119-26.

22. Röhr S, Müller F, Jung F, Apfelbacher C, Seidler A, Riedel-Heller SG. [Psychosocial Impact of Quarantine Measures During Serious Coronavirus Outbreaks: A Rapid Review]. Psychiatrische Praxis. 2020;47(4):179-89.

23. Ryu S, Gao H, Wong JY, Shiu EYC, Xiao J, Fong MW, et al. Nonpharmaceutical Measures for Pandemic Influenza in Nonhealthcare Settings-International Travel- Related Measures. Emerging infectious diseases. 2020;26(5):961-6.

24. Selvey LA, Antão C, Hall R. Entry screening for infectious diseases in humans. Emerging infectious diseases. 2015;21(2):197-201.

25. Taghrir MH, Akbarialiabad H, Ahmadi Marzaleh M. Efficacy of Mass Quarantine as Leverage of Health System Governance During COVID-19 Outbreak: A Mini Policy Review. Archives of Iranian medicine. 2020;23(4):265-7.



For peer review only - http://bmjopen.bmj.com/site/about/guidelines.xhtml

26. Vaidya R, Herten-Crabb A, Spencer J, Moon S, Lillywhite L. Travel restrictions and infectious disease outbreaks. Journal of Travel Medicine. 2020.

27. Walters CE, Meslé MMI, Hall IM. Modeling the global spread of diseases: A review of current practice and capability. Epidemics. 2018;25:1-8.

28. Wilder-Smith A. The severe acute respiratory syndrome: impact on travel and tourism. Travel medicine and infectious disease. 2006;4(2):53-60.

29. Wilder-Smith A, Paton NI, Goh KT. Experience of severe acute respiratory syndrome in singapore: importation of cases, and defense strategies at the airport. Journal of travel medicine. 2003;10(5):259-62.

30. WHO. Handbook for public health capacity-building at ground crossings and cross-border collaboration. World Health Organization; 2020.

For peer review only

**S4:** Data extraction form

Study information:

- Study ID
- Study title
- Publication year
- Study source (journal, report, pre-print publication)
- For pre-print publication only: date of publication

Study design:

- Study type (e.g. modeling study, cross-sectional study, econometric study)
- Verbal summary of study (e.g., stochastic discrete event simulation model)
- Comments

Population/context:

- Country in which measure is implemented
- Study setting (e.g., Auckland international airport)
- Region protected by travel-related measure
- Region restricted by travel-related measure
- Short description of population studied (e.g., international travelers including flight passenger and crew members arriving between 27 April and 22 June 2009)
- Interconnectedness of the region
- Comments

Characteristics of pathogen/disease:

- Pathogen/disease (e.g., COVID-19, influenza)
- For influenza only: subtype (e.g. H1N1), year, endemic vs. pandemic
- Real vs. hypothetical disease outbreak
- Comments

Intervention:

- Broad measure category (e.g., entry screening)
- Verbal summary of specific travel-related measure(s) (e.g., in-flight questionnaire/symptoms)
- Real vs. hypothetical intervention
- Implementation of travel-related measure
- Phase of disease/epidemic spread (e.g. early-stage vs. late-stage)
- Co-interventions (assessed in the study)
- Co-interventions (not assessed in the study)
- Comments

Primary outcomes (repeated for each primary outcome):

- Outcome category (i.e. infectious-disease related health outcome, other health outcome, economic outcome, social outcome)
- Description of outcome
- Outcome attributable to travel-related measures (yes/no)
- Length of follow-up



For peer review only - http://bmjopen.bmj.com/site/about/guidelines.xhtml

- Comments

Secondary outcomes

- Description of any secondary (non-quantitative) outcomes

For peer review only



For peer review only - http://bmjopen.bmj.com/site/about/guidelines.xhtml

**S5:** Studies included in the evidence map

1. Adekunle AI, Meehan M, Rojaz Alvarez D, Trauer J, McBryde E. Delaying the COVID-19 epidemic in Australia: Evaluating the effectiveness of international travel bans. medRxiv. 2020:2020.03.22.20041244.

2. Aleta A, Hu Q, Ye J, Ji P, Moreno Y. A data-driven assessment of early travel restrictions related to the spreading of the novel COVID-19 within mainland China. medRxiv. 2020:2020.03.05.20031740.

3. Anonymous. Thermal image scanners to detect fever in airline passengers, Vancouver and Toronto, 2003. Canada Communicable Disease Report.30(19):165-7.

4. Anzai A, Kobayashi T, Linton NM, Kinoshita R, Hayashi K, Suzuki A, et al. Assessing the Impact of Reduced Travel on Exportation Dynamics of Novel Coronavirus Infection (COVID-19). Journal of Clinical Medicine. 2020;9(2):24.

5. Arima Y, Shimada T, Suzuki M, Suzuki T, Kobayashi Y, Tsuchihashi Y, et al. Severe Acute Respiratory Syndrome Coronavirus 2 Infection among Returnees to Japan from Wuhan, China, 2020. Emerging Infectious Diseases. 2020;26(7):10.

6. Arino J, Jordan R, van den Driessche P. Quarantine in a multi-species epidemic model with spatial dynamics. Mathematical Biosciences. 2007;206(1):46-60.

7. Bajardi P, Poletto C, Ramasco JJ, Tizzoni M, Colizza V, Vespignani A. Human mobility networks, travel restrictions, and the global spread of 2009 H1N1 pandemic. PLoS ONE. 2011;6(1).

8. Banholzer N, van Weenen E, Kratzwald B, Seeliger A, Tschernutter D, Bottrighi P, et al. Estimating the impact of non-pharmaceutical interventions on documented infections with COVID-19: A cross-country analysis. medRxiv. 2020:2020.04.16.20062141.

9. Bolton KJ, McCaw JM, Moss R, Morris RS, Wang S, Burma A, et al. Likely effectiveness of pharmaceutical and non-pharmaceutical interventions for mitigating influenza virus transmission in Mongolia. Bull WHO. 2012;90(4):264-71.

10. Boyd M, Baker MG, Mansoor OD, Kvizhinadze G, Wilson N. Protecting an island nation from extreme pandemic threats: Proof-of-concept around border closure as an intervention. PLoS ONE [Electronic Resource]. 2017;12(6):e0178732.

11. Boyd M, Mansoor OD, Baker MG, Wilson N. Economic evaluation of border closure for a generic severe pandemic threat using New Zealand Treasury methods. Australian & New Zealand Journal of Public Health. 2018;42(5):444-6.

12. Caley P, Becker NG, Philip DJ. The waiting time for inter-country spread of pandemic influenza. PLoS ONE. 2007;2(1).

13. Chang M-C, Kahn R, Li Y-A, Lee C-S, Buckee CO, Chang H-H. Modeling the impact of human mobility and travel restrictions on the potential spread of SARS-CoV-2 in Taiwan. medRxiv. 2020:2020.04.07.20053439.

14. Cheng HY, Li SY, Yang CH. Initial rapid and proactive response for the COVID-19 outbreak - Taiwan's experience. Journal of the Formosan Medical Association. 2020;119(4):771-3.

For peer review only

15. Chinazzi M, Davis JT, Ajelli M, Gioannini C, Litvinova M, Merler S, et al. The effect of travel restrictions on the spread of the 2019 novel coronavirus (COVID-19) outbreak. Science. 2020;368(6489):395-400.

16. Chiyomaru K, Takemoto K. Global COVID-19 transmission rate is influenced by precipitation seasonality and the speed of climate temperature warming. medRxiv. 2020:2020.04.10.20060459.

17. Chong KC, Ying Zee BC. Modeling the impact of air, sea, and land travel restrictions supplemented by other interventions on the emergence of a new influenza pandemic virus. BMC Infect Dis. 2012;12.

18. Chung LH. Impact of pandemic control over airport economics: Reconciling public health with airport business through a streamlined approach in pandemic control. J Air Transp Manage. 2015;44-45:42-53.

19. Ciofi degli Atti ML, Merler S, Rizzo C, Ajelli M, Massari M, Manfredi P, et al. Mitigation measures for pandemic influenza in Italy: An individual based model considering different scenarios. PLoS ONE. 2008;3(3).

20. Clifford SJ, Pearson CAB, Klepac P, Z V, voort K, Quilty BJ, et al. Interventions targeting air travellers early in the pandemic may delay local outbreaks of SARS-CoV-2. medRxiv. 2020:2020.02.12.20022426.

21. Colizza V, Barrat A, Barthelemy M, Valleron AJ, Vespignani A. Modeling the worldwide spread of pandemic influenza: Baseline case and containment interventions. PLoS Med. 2007;4(1):0095-110.

22. Cooper BS, Pitman RJ, Edmunds WJ, Gay NJ. Delaying the international spread of pandemic influenza. PLoS Med. 2006;3(6):0845-55.

23. Costantino V, Heslop DJ, MacIntyre C, Raina i. The effectiveness of full and partial travel bans against COVID-19 spread in Australia for travellers from China. medRxiv. 2020:2020.03.09.20032045.

24. Cowling BJ, Ali ST, Ng TWY, Tsang TK, Li JCM, Fong MW, et al. Impact assessment of non-pharmaceutical interventions against coronavirus disease 2019 and influenza in Hong Kong: an observational study. The lancet Public Health. 2020;17:17.

25. Dandekar R, Barbastathis G. Quantifying the effect of quarantine control in Covid-19 infectious spread using machine learning. medRxiv. 2020:2020.04.03.20052084.

26. De Vlas SJ, Feng D, Cooper BS, Fang LQ, Cao WC, Richardus JH. The impact of public health control measures during the SARS epidemic in mainland China. Trop Med Int Health. 2009;14(SUPPL. 1):101-4.

27. Ediriweera DS, de Silva NR, Malavige NG, de Silva HJ. An epidemiological model to aid decision-making for COVID-19 control on Sri Lanka. medRxiv. 2020:2020.04.11.20061481.

28. Eichner M, Schwehm M, Wilson N, Baker MG. Small islands and pandemic influenza: Potential benefits and limitations of travel volume reduction as a border control measure. BMC Infect Dis. 2009;9:160.



For peer review only - http://bmjopen.bmj.com/site/about/guidelines.xhtml

29. Epstein JM, Goedecke DM, Yu F, Morris RJ, Wagener DK, Bobashev GV. Controlling pandemic flu: The value of international air travel restrictions. PLoS ONE. 2007;2(5).

30. Fang H, Wang L, Yang Y. Human Mobility Restrictions and the Spread of the Novel Coronavirus (2019-nCoV) in China. medRxiv. 2020:2020.03.24.20042424.

31. Ferguson NM, Cummings DAT, Fraser C, Cajka JC, Cooley PC, Burke DS. Strategies for mitigating an influenza pandemic. Nature. 2006;442(7101):448-52.

32. Flahault A, Vergu E, Coudeville L, Grais RF. Strategies for containing a global influenza pandemic. Vaccine. 2006;24(44-46):6751-5.

33. Fujita M, Sato H, Kaku K, Tokuno S, Kanatani Y, Suzuki S, et al. Airport quarantine inspection, follow-up observation, and the prevention of pandemic influenza. Aviat Space Environ Med. 2011;82(8):782-9.

34. Germann TC, Kadau K, Longini Jr IM, Macken CA. Mitigation strategies for pandemic influenza in the United States. Proc Natl Acad Sci U S A. 2006;103(15):5935-40.

35. Glass K, Becker NG. Evaluation of measures to reduce international spread of SARS. Epidemiol Infect. 2006;134(5):1092-101.

36. Gostic KM, Gomez ACR, Mummah RO, Kucharski AJ, Lloyd-Smith JO. Estimated effectiveness of symptom and risk screening to prevent the spread of COVID-19. eLife. 2020;9.

37. Gostic KM, Kucharski AJ, Lloyd-Smith JO. Effectiveness of traveller screening for emerging pathogens is shaped by epidemiology and natural history of infection. eLife. 2015;2015(4).

38. Goubar A, Bitar D, Cao WC, Feng D, Fang LQ, Desenclos JC. An approach to estimate the number of SARS cases imported by international air travel. Epidemiol Infect. 2009;137(7):1019-31.

39. Gunaratnam PJ, Tobin S, Seale H, Marich A, McAnulty J. Airport arrivals screening during pandemic (H1N1) 2009 influenza in New South Wales, Australia. Med J Aust. 2014;200(5):290-2.

40. Hale MJ, Hoskins RS, Baker MG. Screening for influenza A(H1N1)pdm09, Auckland International Airport, New Zealand. Emerging Infectious Diseases. 2012;18(5):866-8.

41. Hamidouche M. COVID-19 Epidemic in Algeria: Assessment of the implemented preventive strategy. medRxiv. 2020:2020.04.21.20074443.

42. He J, Chen G, Jiang Y, Jin R, He M, Shortridge A, et al. Comparative Analysis of COVID-19 Transmission Patterns in Three Chinese Regions vs. South Korea,Italy and Iran. medRxiv. 2020:2020.04.09.20053223.

43. Hien TT, Boni MF, Bryant JE, Ngan TT, Wolbers M, Nguyen TD, et al. Early pandemic influenza (2009 H1N1) in Ho Chi Minh City, Vietnam: a clinical virological and epidemiological analysis. PLoS Medicine / Public Library of Science.7(5):e1000277.

44. Hollingsworth TD, Ferguson NM, Anderson RM. Will travel restrictions control the international spread of pandemic influenza? [3]. Nat Med. 2006;12(5):497-9.



For peer review only - http://bmjopen.bmj.com/site/about/guidelines.xhtml

45. Hossain MP, Junus A, Zhu X, Jia P, Wen T-H, Pfeiffer D, et al. The effects of border control and quarantine measures on global spread of COVID-19. medRxiv. 2020:2020.03.13.20035261.

46. Hou J, Hong J, Ji B, Dong B, Chen Y, Ward MP, et al. Changing transmission dynamics of COVID-19 in China: a nationwide population-based piecewise mathematical modeling study. medRxiv. 2020:2020.03.27.20045757.

47. Hsieh YH, King CC, Chen CW, Ho MS, Hsu SB, Wu YC. Impact of quarantine on the 2003 SARS outbreak: a retrospective modeling study. Journal of Theoretical Biology. 2006;244(4):729-36.

48. Hsieh YH, Van Den Driessche P, Wang L. Impact of travel between patches for spatial spread of disease. Bulletin of Mathematical Biology. 2007;69(4):1355-75.

49. Jia JS, Lu X, Yuan Y, Xu G, Jia J, Christakis NA. Population flow drives spatio-temporal distribution of COVID-19 in China. Nature. 2020.

50. Jiang X, eng, Chang L, Shi Y. How does the outbreak of 2019-nCoV spread in mainland China? A retrospective analysis of the dynamic transmission routes. medRxiv. 2020:2020.03.01.20029645.

51. Kerneis S, Grais RF, Boelle PY, Flahault A, Vergu E. Does the effectiveness of control measures depend on the influenza pandemic profile? PLoS ONE [Electronic Resource]. 2008;3(1):e1478.

52. Khan K, Eckhardt R, Brownstein JS, Naqvi R, Hu W, Kossowsky D, et al. Entry and exit screening of airline travellers during the A(H1N1) 2009 pandemic: A retrospective evaluation. Bull WHO. 2013;91(5):368-76.

53. Kim S, Chang DE, editors. Border screening vs. community level disease control for infectious diseases: Timing and effectiveness2017: American Institute of Physics Inc.

54. Kong XS, Liu F, Wang HB, Yang RF, Chen DB, Wang XX, et al. Epidemic prevention and control measures in China significantly curbed the epidemic of COVID-19 and influenza. medRxiv. 2020:2020.04.09.20058859.

55. Kraemer MUG, Yang CH, Gutierrez B, Wu CH, Klein B, Pigott DM, et al. The effect of human mobility and control measures on the COVID-19 epidemic in China. Science. 2020;25:25.

56. Kuo JS, Lee YH, Heish JW, Lin MC, Yang SY. Initial evaluation on screening of novel influenza A (H1N1) at international ports in Taiwan. Taiwan Epidemiol Bull. 2009;25(9):254-67.

57. Lai S, Ruktanonchai NW, Zhou L, Prosper O, Luo W, Floyd JR, et al. Effect of non-pharmaceutical interventions for containing the COVID-19 outbreak in China. medRxiv. 2020:2020.03.03.20029843.

58. Lam EHY, Cowling BJ, Cook AR, Wong JYT, Lau MSY, Nishiura H. The feasibility of age-specific travel restrictions during influenza pandemics. Theor Biol Med Model. 2011;8(1).

59. Lau H et al.. Positive impact of lockdown in Wuhan on containing the COVID-19 outbreak in China ;Journal of Travel Medicine; Oxford Academic. Journal of Travel Medicine. 2020.

60. Lee JM, Choi D, Cho G, Kim Y. The effect of public health interventions on the spread of influenza among cities. Journal of Theoretical Biology. 2012;293:131-42.

For peer review only

61. Li D, Liu Z, Liu Q, Gao Z, Zhu J, Yang J, et al. Estimating the Efficacy of Traffic Blockage and Quarantine for the Epidemic Caused by 2019-nCoV (COVID-19). medRxiv. 2020:2020.02.14.20022913.

62. Lin H, Liu W, Gao H, Nie J, Fan Q. Trends in Transmissibility of 2019 Novel Coronavirus-infected Pneumonia in Wuhan and 29 Provinces in China. medRxiv. 2020:2020.02.21.20026468.

63. Linka K, Peirlinck M, Sahli Costabal F, Kuhl E. Outbreak dynamics of COVID-19 in Europe and the effect of travel restrictions. medRxiv. 2020:2020.04.18.20071035.

64. Liu H, Bai X, Shen H, Pang X, Liang Z, Liu Y. Synchronized travel restrictions across cities can be effective in COVID-19 control. medRxiv. 2020a:2020.04.02.20050781.

65. Liu K, Ai S, Song S, Zhu G, Tian F, Li H, et al. Population movement, city closure in Wuhan and geographical expansion of the 2019-nCoV pneumonia infection in China in January 2020. Clinical infectious diseases. 2020.

66. Liu XN, Takeuchi Y. Spread of disease with transport-related infection and entry screening. Journal of Theoretical Biology. 2006;242(2):517-28.

67. Malmberg H, Britton T. Inflow restrictions can prevent epidemics when contact tracing efforts are effective but have limited capacity. medRxiv. 2020:2020.04.01.20050401.

68. Malone JD, Brigantic R, Muller GA, Gadgil A, Delp W, McMahon BH, et al. U.S. airport entry screening in response to pandemic influenza: Modeling and analysis. Travel Med Infect Dis. 2009;7(4):181-91.

69. Mandal S, Bhatnagar T, Arinaminpathy N, Agarwal A, Chowdhury A, Murhekar M, et al. Prudent public health intervention strategies to control the coronavirus disease 2019 transmission in India: A mathematical model-based approach. Indian Journal of Medical Research. 2020;23:23.

70. Marcelino J, Kaiser M. Critical paths in a metapopulation model of H1N1: Efficiently delaying influenza spreading through flight cancellation. PLoS Currents. 2012.

71. Mbuvha R, Marwala T. Bayesian Inference of COVID-19 Spreading Rates in South Africa. medRxiv. 2020:2020.04.28.20083873.

72. Mondal S, Ghosh S. Searching the Sigmoid-type trend in Lock Down period covid19 data of India and its different states. medRxiv. 2020:2020.04.25.20079624.

73. Moriarty L. et al., Prevention, et al. Public Health Responses to COVID-19 Outbreaks on Cruise Ships — Worldwide, February–March 2020. MMWR Morb Mortal Wkly Rep. 2020.

74. Mummert A, Weiss H, Long LP, Amigo JM, Wan XF. A perspective on multiple waves of influenza pandemics. PLoS ONE [Electronic Resource]. 2013;8(4):e60343.

75. Muraduzzaman AKM, Khan MH, Parveen R, Sultana S, Alam AN, Akram A, et al. Event based surveillance of Middle East Respiratory Syndrome Coronavirus (MERS- CoV) in Bangladesh among pilgrims and travelers from the Middle East: An update for the period 2013-2016. PLoS ONE [Electronic Resource]. 2018;13(1):e0189914.




For peer review only - http://bmjopen.bmj.com/site/about/guidelines.xhtml

76. Nakata Y, Rost G. Global analysis for spread of infectious diseases via transportation networks. Journal of Mathematical Biology. 2015;70(6):1411-56.

77. Nigmatulina KR, Larson RC. Living with influenza: Impacts of government imposed and voluntarily selected interventions. European Journal of Operational Research. 2009;195(2):613-27.

78. Nishiura H, Wilson N, Baker MG. Quarantine for pandemic influenza control at the borders of small island nations. BMC Infect Dis. 2009;9.

79. Odendaal WG. A Method to Model Outbreaks of New Infectious Diseases with Pandemic Potential such as COVID-19. medRxiv. 2020:2020.03.11.20034512.

80. Pan J, Yao Y, Liu Z, Li M, Wang Y, Dong W, et al. Effectiveness of control strategies for Coronavirus Disease 2019: a SEIR dynamic modeling study. medRxiv. 2020:2020.02.19.20025387.

81. Pan X, Ojcius DM, Gao T, Li Z, Pan C, Pan C. Lessons learned from the 2019-nCoV epidemic on prevention of future infectious diseases. Microbes Infect. 2020a;22(2):86-91.

82. Pang X, Zhu Z, Xu F, Guo J, Gong X, Liu D, et al. Evaluation of Control Measures Implemented in the Severe Acute Respiratory Syndrome Outbreak in Beijing, 2003. J Am Med Assoc. 2003;290(24):3215-21.

83. Pinkas J, Jankowski M, Szumowski L, Lusawa A, Zgliczynski WS, Raciborski F, et al. Public Health Interventions to Mitigate Early Spread of SARS-CoV-2 in Poland. Medical Science Monitor. 2020;26:e924730.

84. Pitman RJ, Cooper BS, Trotter CL, Gay NJ, Edmunds WJ. Entry screening for severe acute respiratory syndrome (SARS) or influenza: Policy evaluation. Br Med J. 2005;331(7527):1242-3.

85. Priest PC, Jennings LC, Duncan AR, Brunton CR, Baker MG. Effectiveness of border screening for detecting influenza in arriving airline travelers. American Journal of Public Health. 2013;103(8):1412-8.

86. Pullano G, Pinotti F, Valdano E, Boelle PY, Poletto C, Colizza V. Novel coronavirus (2019-nCoV) early-stage importation risk to Europe, January 2020. Euro Surveillance: Bulletin Europeen sur les Maladies Transmissibles = European Communicable Disease Bulletin.25(4):01.

87. Qiu Y, Chen X, Shi W. Impacts of social and economic factors on the transmission of coronavirus disease (COVID-19) in China. medRxiv. 2020:2020.03.13.20035238.

88. Quilty BJ, Diamond C, Liu Y, Gibbs H, Russell TW, Jarvis CI, et al. The effect of inter-city travel restrictions on geographical spread of COVID-19: Evidence from Wuhan, China. medRxiv. 2020:2020.04.16.20067504.

89. Ray D, Salvatore M, Bhattacharyya R, Wang L, Mohammed S, Purkayastha S, et al. Predictions, role of interventions and effects of a historic national lockdown in India's response to the COVID-19 pandemic: data science call to arms. medRxiv. 2020:2020.04.15.20067256.



For peer review only - http://bmjopen.bmj.com/site/about/guidelines.xhtml

90. Sakaguchi H, Tsunoda M, Wada K, Ohta H, Kawashima M, Yoshino Y, et al. Assessment of border control measures and community containment measures used in japan during the early stages of Pandemic (H1N1) 2009. PLoS ONE. 2012;7(2).

91. Samaan G, Patel M, Spencer J, Roberts L. Border screening for SARS in Australia: What has been learnt? Med J Aust. 2004;180(5):220-4.

92. Sang Z, Qiu ZP, Yan XF, Zou Y. Assessing the effect of non-pharmaceutical interventions on containing an emerging disease.. Mathematical Biosciences and Engineering.9(1):147-64.

93. Scala A, Flori A, Spelta A, ro, Brugnoli E, Cinelli M, et al. Between Geography and Demography: Key Interdependencies and Exit Mechanisms for Covid-19. medRxiv. 2020:2020.04.09.20059592.

94. Scalia Tomba G, Wallinga J. A simple explanation for the low impact of border control as a countermeasure to the spread of an infectious disease. Mathematical Biosciences.214(1):70-2.

95. Shi Z, Fang Y. Temporal relationship between outbound traffic from Wuhan and the 2019 coronavirus disease (COVID-19) incidence in China. medRxiv. 2020:2020.03.15.20034199.

96. Song PX, Wang L, Zhou Y, He J, Zhu B, Wang F, et al. An epidemiological forecast model and software assessing interventions on COVID-19 epidemic in China. medRxiv. 2020:2020.02.29.20029421.

97. St. John RK, King A, De Jong D, Bodie-Collins M, Squires SG, Tam TWS. Border screening for SARS. Emerging Infectious Diseases. 2005;11(1):6-10.

98. Su L, Hong N, Zhou X, He J, Ma Y, Jiang H, et al. Evaluation of the secondary transmission pattern and epidemic prediction of COVID-19 in the four metropolitan areas of China. medRxiv. 2020.

99. Tang B, Wang X, Li Q, Bragazzi NL, Tang S, Xiao Y, et al. Estimation of the Transmission Risk of the 2019-nCoV and Its Implication for Public Health Interventions. Journal of Clinical Medicine.9(2):07.

100. Tian H, Liu Y, Li Y, Wu C-H, Chen B, Kraemer MUG, et al. The impact of transmission control measures during the first 50 days of the COVID-19 epidemic in China. medRxiv. 2020:2020.01.30.20019844.

101. Tsuboi M, Hachiya M, Noda S, Iso H, Umeda T. Epidemiology and quarantine measures during COVID-19 outbreak on the cruise ship Diamond Princess docked at Yokohama, Japan in 2020: a descriptive analysis. Global Health & Medicine. 2020:2020.01037-2020.

102. Wang F, Li Y, Tang D, Li Q, Liu G, Wang J, et al. Trend of the Coronavirus Disease-2019 Epidemic in China After the Lockdown of Wuhan City on January 23, 2020. SSRN. 2020.

103. Wang L, Zhang Y, Huang T, Li X. Estimating the value of containment strategies in delaying the arrival time of an influenza pandemic: a case study of travel restriction and patient isolation. Physical Review E Statistical, Nonlinear, & Soft Matter Physics. 2012;86(3):032901.

For peer review only



For peer review only - http://bmjopen.bmj.com/site/about/guidelines.xhtml

104. Wang TH, Wei KC, Hsiung CA, Maloney SA, Eidex RB, Posey DL, et al. Optimizing severe acute respiratory syndrome response strategies: lessons learned from quarantine. American Journal of Public Health. 2007;97:S98-100.

105. Wang X, Liu S, Wang L, Zhang W. An Epidemic Patchy Model with Entry-Exit Screening. Bulletin of Mathematical Biology. 2015;77(7):1237-55.

106. Wells CR, Sah P, Moghadas SM, Pandey A, Shoukat A, Wang Y, et al. Impact of international travel and border control measures on the global spread of the novel 2019 coronavirus outbreak. Proc Natl Acad Sci U S A. 2020;117(13):7504-9.

107. Weng W, Ni S. Evaluation of containment and mitigation strategies for an influenza A pandemic in China. Simulation. 2015;91(5):407-16.

108. Wilder-Smith A, Goh KT, Paton NI. Experience of Severe Acute Respiratory Syndrome in Singapore: Importation of Cases, and Defense Strategies at the Airport. Journal of Travel Medicine. 2003;10(5):259-62.

109. Wood JG, Zamani N, Raina MacIntyre C, Becker NG. Effects of internal border control on spread of pandemic influenza. Emerging Infectious Diseases. 2007;13(7):1038-45.

110. Yang Z, Zeng Z, Wang K, Wong SS, Liang W, Zanin M, et al. Modified SEIR and AI prediction of the epidemics trend of COVID-19 in China under public health interventions. Journal of Thoracic Disease. 2020;12(3):165-74.

111. Ying S, Li F, Geng X, Li Z, Du X, Chen H, et al. Spread and control of COVID-19 in China and their associations with population movement, public health emergency measures, and medical resources. medRxiv. 2020:2020.02.24.20027623.

112. Yu H, Cauchemez S, Donnelly CA, Zhou L, Feng L, Xiang N, et al. Transmission dynamics, border entry screening, and school holidays during the 2009 influenza A (H1N1) pandemic, China. Emerging Infectious Diseases. 2012;18(5):758-66.

113. Yuan H-Y, Hossain MP, Tsegaye MM, Zhu X, Jia P, Wen T-H, et al. Estimating the risk on outbreak spreading of 2019-nCoV in China using transportation data. medRxiv. 2020:2020.02.01.20019984.

114. Yuan Z, Xiao Y, Dai Z, Huang J, Chen Y. A simple model to assess Wuhan lock-down effect and region efforts during COVID-19 epidemic in China Mainland. medRxiv. 2020a;2020a:2020.02.29.20029561.

115. Zhang B, Zhou H, Zhou F. Study on SARS-COV-2 transmission and the effects of control measures in China. medRxiv. 2020a:2020.02.16.20023770.

116. Zhang C, Chen C, Shen W, Tang F, Lei H, Xie Y, et al. Impact of population movement on the spread of 2019-nCoV in China. Emerging Microbes & Infections. 2020:1-28.

117. Zhang N, Zhao PC, Li YG. Increased infection severity in downstream cities in infectious disease transmission and tourists surveillance analysis. Journal of Theoretical Biology. 2019;470:20-9.

For peer review only

118. Zhang WD, Zu ZH, Xu Q, Xu ZJ, Liu JJ, Zheng T. Optimized Strategy for the Control and Prevention of Newly Emerging Influenza Revealed by the Spread Dynamics Model. PLoS ONE. 2014;9(1):11.

119. Zhang Y, Yang P, Liyanage S, Seale H, Deng Y, Pang X, et al. The characteristics of imported cases and the effectiveness of outbreak control strategies of pandemic influenza A (H1N1) in China. Asia-Pac J Public Health. 2012;24(6):932-9.

120. Zhao S, Chen H. Modeling the epidemic dynamics and control of COVID-19 outbreak in China. Quantitative Biology. 2020:1-9.

121. Zhou X, Wu Z, Yu R, Cao S, Fang W, Jiang Z, et al. Modeling-based evaluation of the effect of quarantine control by the Chinese government in the coronavirus disease 2019 outbreak. medRxiv. 2020:2020.03.03.20030445.

122. Zlojutro A, Rey D, Gardner L. A decision-support framework to optimize border control for global outbreak mitigation. Sci Rep. 2019;9(1).

For peer review only

**S6:** Interventions considered in the included studies

| Study ID | Short description of intervention | Phase of implementation |
|---|---|---|
| **Border closure** | | |
| **Studies assessing COVID-19** | | |
| Banholzer 2020 | Closure of national borders for individuals | Local transmission phase |
| **Studies assessing a hypothetical disease (COVID-19 relevant)** | | |
| Boyd 2018 | n.r. | Early phase |
| Boyd 2017 | Closure of national borders (ending the arrival of all commercial passenger planes, private planes, cruise ships, and yachts) | Early phase |
| **Entry/exit screening** | | |
| **Studies assessing COVID-19** | | |
| Clifford 2020 | • Entry screening (at departure)<br>• Exit screening (on arrival)<br>• Follow-up quarantine of positive cases<br>• Follow-up information/handouts to all arriving passengers | Early phase |
| Gostic 2020 | • Entry screening (all arriving passengers; temperature screening followed by a questionnaire on exposure risk)<br>• Exit screening (all departing passengers; temperature screening followed by a questionnaire on exposure risk)<br>• Follow up detainment of potentially infected passengers | Unclear phase |

| Study ID | Short description of intervention | Phase of implementation |
|---|---|---|
| Mandal 2020 | • Entry screening (passengers from China, Hong Kong, Singapore, Thailand, Japan, South Korea, Iran, and Italy; focus on temperature screening; advise for symptomatic passengers to volunteer for screening)<br>· Follow-up quarantine of positive cases | Early phase |
| **Studies assessing influenza** | | |
| Fujita 2011 | · Entry screening (on-board screening at Narita International Airport; questionnaire and thermography)<br>· Follow-up medical assessment (rapid tests followed by RT-PCR)<br>· Follow-up quarantine of positive cases and those sitting nearby<br>· Follow-up daily monitoring of travelers with negative screening results | Unclear phase |
| Gunaratnam 2014 | · Entry screening (all international arrivals at Sydney Airport; health declaration and thermal scanning)<br>· Follow-up medical assessment (swab taken) | Unclear phase |
| Hale 2012 | · Entry screening (all international arrivals at Auckland International Airport; health card, self-presentation)<br>· Follow up medical assessment (ILI case definition, RT-PCR)<br>· Quarantine of positive cases | Unclear phase |
| Hien 2010 | · Entry screening (all international arrivals; temperature screening; symptom questionnaire)<br>· Follow up quarantine of suspected cases (in hospitals) | Local transmission phase |
| Khan 2013 | · Entry screening (targeted: passengers arriving at international airports after flying from Mexico on a direct flight or indiscriminate entry screening on a direct or a connecting flight)<br>· Exit screening (passengers departing from any international airport in Mexico) | Unclear phase |
| Kuo 2009 | · Entry screening (on-board screening of passengers arriving from epidemic affected areas, such as Mexico, USA, and Canada before passengers leave their seats; the procedure soon adjusted for only those airplanes reporting ill passengers on board)<br>· Follow-up quarantine of positive cases (hospital) | Unclear phase |

| Study ID | Short description of intervention | Phase of implementation |
|---|---|---|
| Malone 2009 | · Entry screening (all international passengers arriving at 18 US airports from Asia, Europe, South America and Canada: includes pre-arrival, primary active, and secondary active surveillance measures)<br>· Follow up quarantine of positive cases | Local transmission phase |
| Mummert 2013 | · Entry screening (thermal scanners)<br>· Follow-up quarantine of suspected cases and those seating nearby | Local transmission phase |
| Priest 2013 | · Entry screening (for all passengers arriving from Australia; all completed a health questionnaire, randomly chosen passengers (50%) screened for temperature and had throat and nasal swabs taken ) | Unclear phase |
| Sakaguchi 2012 | · Entry screening (all passengers arriving from other countries; infrared thermos-scanner, a health declaration form)<br>· Follow-up medical assessment (rapid influenza test, RT-PCR)<br>· Follow-up quarantine of positive cases and close contacts (seating next to them in the airplane)<br>· Follow-up information/handouts (all asymptomatic passengers)<br>· Follow-up health monitoring for fellow travelers of infected individuals identified during entry screening | Unclear phase |
| Wang 2015 | Different scenarios were considered in the model:<br>· Indiscriminate entry and exit screening in each patch<br>· Indiscriminate entry screening only in each patch<br>· Targeted entry screening (travel to and from high risk patch only)<br>· Selective entry screening (only the high risk patch and those closely connected patches)<br>· One-way entry screening in low-risk patches only | Local transmission phase |
| Yu 2012 | · Entry screening (all passengers at any point of entry into China from another country or from a neighboring region)<br>· Follow-up quarantine of suspected cases (in designated hospitals) | Local transmission phase; Unclear phase |
| Zhang 2014 | · Entry screening (temperature screening at ports of entry) | Early phase |
| Zhang 2012 | · Entry screening (temperature screening, questionnaire)<br>· Follow-up medical assessment (designated hospital)<br>· Quarantine of positive cases and close contacts | Unclear phase |

| Study ID | Short description of intervention | Phase of implementation |
|---|---|---|
| | · Medical follow-up of all travelers from overseas (daily contacts for 7 days)<br>· Follow-up ILI screening in hospitals | |
| Zlojutro 2018 | · Entry screening (in airports)<br>· Follow up quarantine of positive cases | Early phase |
| **Studies assessing SARS** | | |
| Anonymous | · Entry screening (all passengers arriving from affected areas; questionnaire, thermal image scanners)<br>· Exit screening (all departing passengers; thermal image scanners)<br>· Follow-up medical assessment (oral temperature screening, standardized questionnaire for SARS) | Local transmission phase;<br>Unclear phase |
| Glass 2006 | · Border screening (health declaration cards)<br>· Follow-up Information/handouts to travelers | Early phase;<br>Unclear phase |
| Goubar 2008 | · Entry screening (for symptomatic travelers only) | Early phase;<br>Unclear phase |
| Samaan 2004 | · Entry screening (identification through self-referral, detection by airline staff or airport custom staff; Australian Quarantine and Inspection Service conducting basic screening)<br>· Follow up screening (temperature screening by nursing staff)<br>· Follow-up screening (chief quarantine officer applies case definition)<br>· Follow-up medical assessment (in a hospital) | Early phase;<br>Local transmission phase |
| Pang 2003 | · Border screening (at the Beijing airport, major train stations, and 71 roads connecting Beijing to other areas) | Local transmission phase |
| Sang 2012 | · Entry screening | Unclear phase |
| St. John 2005 | · Entry screening (all arriving passengers; questionnaire, thermal scanners)<br>· Exit screening (all departing passengers; questionnaire, thermal scanners)<br>· Follow-up: Information/handouts to all arriving passengers<br>· Follow-up medical assessment<br>· Detainment and further medical assessment (in a hospital) | Unclear phase |

| Study ID | Short description of intervention | Phase of implementation |
|---|---|---|
| Wilder-Smith 2003 | Different entry and exit screening procedures rolled out at different times:<br>· Entry screening (all passengers arriving from SARS-affected areas; visual screening and further temperature screening; health declaration)<br>· Entry screening (all arriving passengers in the airport and road entry points and further ferry terminals; temperature screening)<br>· Exit screening (all departing passengers in the airport and road entry points and further ferry terminals; temperature screening)<br>· Entry screening (all arriving passengers screened for SARS)<br>· Follow-up medical assessment | Early phase; (or post-peak phase) |
| **Studies assessing MERS** | | |
| Muraduzzaman, 2017 | · Entry screening (Bangladeshi travelers/pilgrims returning from the Middle East)<br>· Follow-up medical assessment<br>· Follow up quarantine of suspected cases | Unclear phase |
| **Studies assessing multiple diseases (SARS, MERS, and/or Influenza)** | | |
| Gostic 2015 | · Entry screening (all arriving passengers; temperature screening followed by a questionnaire on exposure risk)<br>· Exit screening (all departing passengers; temperature screening followed by a questionnaire on exposure risk)<br>· Follow up detainment of potentially infected passengers | Unclear phase |
| Pitman 2005 | · Entry screening | Unclear phase |
| **Studies assessing a hypothetical disease (COVID-19 relevant)** | | |
| Kim 2017 | n.r. | Unclear phase |
| Liu 2006 | · Entry screening<br>· Exit screening<br>· Follow-up quarantine of positive cases | Local transmission phase |
| **Travel ban** | | |

| Study ID | Short description of intervention | Phase of implementation |
|---|---|---|
| **Studies assessing COVID-19** | | |
| Adekunle 2020 | Ban on China, Iran, South Korea, and Italy | Local transmission phase |
| Chiyomaru 2020 | Three scenarios considered:<br>· Global ban<br>· Global quarantine<br>· Non-global restrictions | Local transmission phase |
| Costantino 2020 | Three scenarios modelled:<br>· No ban<br>· Current ban followed by a full lifting<br>· Partial lifting of the current ban (allowing over 100,000 university students to enter Australia, but not tourists) | Local transmission phase;<br>Post-peak phase |
| Mbuvha 2020 | n.r. | Local transmission phase |
| Mondal 2020 | Shutting of airlines and rail transport in India | Local transmission phase |
| Pullano 2020 | Ban on international flights in Hubei (as an example) | Early phase |
| Ray 2020 | · Main scenario: national lockdown<br>· Alternative scenario A: mild action: social distancing and travel ban<br>· Alternative scenario B: no action | Local transmission phase |
| **Studies assessing influenza** | | |
| Hsieh 2007 | n.r. | Local transmission phase |
| **Travel-related quarantine** | | |
| **Studies assessing COVID-19** | | |



For peer review only - http://bmjopen.bmj.com/site/about/guidelines.xhtml

| Study ID | Short description of intervention | Phase of implementation |
|---|---|---|
| Moriatry 2020 | Quarantine of travelers (14 days either on the ship or on the land (port of Oakland) before disembarkation or onward travel) | Local transmission phase |
| Tsuboi 2020 | Quarantine of all travelers in their cabins (followed by targeted screening for COVID-19) | Unclear phase |
| **Studies assessing influenza** | | |
| Nishiura 2009 | Quarantine of travelers (all individuals arriving from the country in which the outbreak occurred) | Early phase |
| **Studies assessing SARS** | | |
| de Vlas 2009 | Quarantine of travelers (in airports and stations) | Early phase;<br>Local transmission phase;<br>Post-peak phase |
| Hsieh 2006 | Home quarantine of travelers (anyone who entered the country from affected countries) | Local transmission phase |
| Wang 2007 | Quarantine of travelers (those sitting on the same flight within 3 rows of a person infected with SARS or those returning from WHO–designated SARS-affected areas) | Unclear phase |
| **Travel restrictions** | | |
| **Studies assessing COVID-19** | | |
| Chang 2020 | n.r. | Early phase;<br>Local transmission phase |
| Odendaal 2020 | n.r. | Early phase |

| Study ID | Short description of intervention | Phase of implementation |
| --- | --- | --- |
| Scala 2020 | n.r. | Early phase;<br>Local transmission phase;<br>Post-peak phase |
| **Studies assessing influenza** | | |
| Bolton 2012 | n.r. | Local transmission phase |
| Chong 2012 | Restricting air, land, and maritime travel | Local transmission phase |
| Ciofi 2008 | Restricting air travel | Early phase |
| Colizza 2007 | Restricting air travel | Early phase |
| Cooper 2005 | Restricting air travel | Local transmission phase |
| Eichner 2009 | Restricting air travel | Early phase |
| Epstein 2007 | Restricting air travel | Early phase |
| Flahault 2006 | Restricting air travel | Early phase |
| Germann 2006 | Restricting air travel | Local transmission phase |
| Kerneis 2008 | Restricting air travel | Early phase |
| Lam 2011 | n.r. | Early phase |
| Lee 2012 | n.r. | Early phase |
| Marcelino 2013 | Restricting air travel | Early phase;<br>Local transmission phase |
| Nigmatulina 2009 | n.r. | Early phase;<br>Local transmission phase |



For peer review only - http://bmjopen.bmj.com/site/about/guidelines.xhtml

| Study ID | Short description of intervention | Phase of implementation |
|---|---|---|
| Scalia Tomba 2008 | n.r. | Early phase |
| Wang 2012 | Cancellation of non-essential trips | Early phase |
| Weng 2015 | n.r. | Early phase;<br>Local transmission phase |
| Wood 2007 | Restricting air travel | Early phase |
| **Studies assessing multiple diseases (SARS and influenza)** | | |
| Chung 2015 | Restricting (control measures on) air travel | Unclear phase |
| Hollingsworth 2006 | Restricting air travel | Early phase |
| **Studies assessing a hypothetical disease (COVID-19 relevant)** | | |
| Arino 2007 | n.r. | Early phase |
| Nakata 2015 | n.r. | Early phase;<br>Local transmission phase |
| **Multiple travel-related control measures** | | |
| **Studies assessing COVID-19** | | |
| Aleta 2020 | Lockdown of Wuhan (referred in the study as "Wuhan's quarantine") | Local transmission phase |
| Anzai 2020 | "Travel restriction and border control have been implemented by various countries, either as: (i) complete travel bans, (ii) travel restriction and quarantine—which allows for restriction of healthy individuals, (iii) entry screening for all incoming travelers, or some combination thereof" | Local transmission phase |
| Arima 2020 | Entry/exit screening | Unclear phase |



For peer review only - http://bmjopen.bmj.com/site/about/guidelines.xhtml

| Study ID | Short description of intervention | Phase of implementation |
|---|---|---|
| | · Entry screening (repatriation of nationals from China and oropharyngeal swab sample taken for all)<br>· Follow-up quarantine of positive cases (in a hospital)<br>Travel-related quarantine (all returning from China for 14 days) | |
| Cheng 2020 | Entry/exit screening<br>· Entry screening (on-board temperature screening for all travelers from Wuhan)<br>· Follow-up medical assessment (in designated hospitals)<br>Travel-related quarantine (all returning from Wuhan, Guangdong, Zheijiang, China, Hong Kong, Macau, and later from South Korea and Italy) | Local transmission phase |
| Chinazzi 2020 | n.r. | Local transmission phase |
| Cowling 2020 | Travel ban<br>· Ban on Hubei residents and visitors to Hubei province in the past 14 days<br>· Ban on flights (between Hong Kong and Wuhan)<br>· Ban on entry of non-Hong Kong residents from overseas countries and territories<br>Travel restrictions<br>· Restricting air travel<br>· Restricting land travel<br>Border closure<br>Travel-related quarantine (for travelers from Hubei, then China, South Korea, Iran, Italy, Germany, Japan and Spain, all Schengen area, all overseas countries, Macau and Taiwan). | Local transmission phase |
| Dandekar 2020 | n.r. | Local transmission phase |
| Ediriweera 2020 | · Lockdown (island-wide)<br>· Travel-related quarantine (all inbound passengers) | Local transmission phase |
| Fang 2020 | Lockdown of Wuhan | Local transmission phase |
| Hamidouche 2020 | Travel ban<br>· Ban: air travel (China, Italy, Morocco, France)<br>· Ban: local public transport including rail traffic | Local transmission phase |



For peer review only - http://bmjopen.bmj.com/site/about/guidelines.xhtml

| Study ID | Short description of intervention | Phase of implementation |
|---|---|---|
| | Closure of all land borders and air routes<br>Travel-related quarantine (14 days) | |
| He 2020 | n.r. | Local transmission phase |
| Hossain 2020 | Lockdown of Wuhan (referred in the study as "lockdown, border control, or quarantine measures"). | Local transmission phase |
| Hou 2020 | Lockdown of Wuhan | Local transmission phase |
| Jia 2020 | Lockdown of Wuhan (referred in the study as "quarantine of Wuhan") | Early phase;<br>Local transmission phase |
| Jiang 2020 | n.r. (referred in the study as “travel restrictions in Hubei”) | Local transmission phase |
| Kraemer 2020 | Lockdown of Wuhan (referred in the study as "cordon sanitaire imposed on Wuhan") | Local transmission phase |
| Lai 2020 | Lockdown of Wuhan | Early phase;<br>Local transmission phase |
| Lau 2020 | Lockdown of Wuhan | Early phase;<br>Local transmission phase |
| Li 2020 | Lockdown of Wuhan (referred in the study as "quarantine and traffic blockage") | Early phase;<br>Local transmission phase |
| Lin 2020 | Lockdown of Wuhan (referred in the study as "travel bans into and from Wuhan") | Local transmission phase |
| Linka 2020 | n.r. | Early phase;<br>Local transmission phase |
| Liu 2020 | Lockdown of Wuhan (referred in the study as "city closure") | Early phase;<br>Local transmission phase |

| Study ID | Short description of intervention | Phase of implementation |
|---|---|---|
| Liu 2020a | Lockdown of Wuhan and travel restrictions in other cities | Early phase;<br>Local transmission phase |
| Pan 2020 | Various interventions strategies in China, including travel restrictions and voluntary quarantine | Local transmission phase |
| Pan 2020a | “Strict traffic restrictions” implemented by the Chinese government. | Local transmission phase |
| Pinkas 2020 | · Border screening (passengers location cards, temperature screening)<br>· Closure of borders to non-citizens<br>· Travel ban: all non-essential travel; suspension of all international air and rail services<br>· Travel-related quarantine | Local transmission phase |
| Qiu 2020 | · Lockdown of Wuhan and other cities in Hubei<br>· Imposed travel restrictions, ranging from "canceling public events, stopping public transportation to limiting how often residents can leave home" | Early phase;<br>Local transmission phase |
| Quilty 2020 | · Lockdown of Wuhan (referred in the study as "cordon sanitaire on the city of Wuhan")<br>· Travel restrictions extended to the whole of Hubei | Local transmission phase |
| Shi 2020 | Lockdown of Wuhan (referred in the study as "Wuhan travel ban") | Local transmission phase |
| Song 2020 | Lockdown of Wuhan (referred in the study as "community-level quarantines such as hospitalization for infected cases, city blockade, traffic control and restricted social activities, and so on") | Local transmission phase |
| Su 2020 | “Official quarantine regulations and travel restrictions in China” | Local transmission phase |
| Tang 2020 | Lockdown of Wuhan and other cities in China (referred in the study as "travel restrictions affecting five cities (Wuhan, Huanggang, Ezhou, Chibi and Zhijiang), effectively shutting down the movement of more than 40 million people") | Local transmission phase |
| Tian 2020 | Lockdown of Wuhan and other cities in China ("China banned travel to and from Wuhan city on 23 January and implemented a national emergency response") | Local transmission phase |



For peer review only - http://bmjopen.bmj.com/site/about/guidelines.xhtml

| Study ID | Short description of intervention | Phase of implementation |
|---|---|---|
| Wang 2020 | Lockdown of Wuhan (referred in the study as "quarantine of Wuhan city") | Local transmission phase |
| Wells 2020 | · Lockdown of Wuhan and some other cities in China<br>· Entry/exit screening in airports (questionnaire, self-reporting) | Local transmission phase |
| Yang 2020 | Lockdown of Wuhan and other cities in China (referred in the study as "large-scale quarantine, strict controls on travel and extensive monitoring of suspected cases) | Local transmission phase |
| Ying 2020 | Lockdown of Wuhan and other cities in Hubei | Local transmission phase |
| Yuan 2020 | Lockdown of Wuhan<br>Two scenarios were considered:<br>· Border control measures without any other control (no mask wearing, isolation, or contact tracing)<br>· Border control with some other control measures | Early phase;<br>Local transmission phase |
| Yuan 2020a | Lockdown of Wuhan "combined with nationwide traffic restrictions" | Local transmission |
| **Studies assessing influenza** | | |
| Bajardi 2011 | Multiple scenarios modelled:<br>· No travel restrictions<br>· Travel ban: air travel (country-specific)<br>· Travel restrictions: land travel | Early phase |
| Caley 2007 | Entry/exit screening<br>· Exit screening (all passengers at departure)<br>· Follow up detainment (those positive are detained and prevented from traveling)<br>· Entry screening (all passengers at arrival)<br>· Follow-up quarantine of all travelers if a positive case is detected<br>Travel restriction: air travel | Early phase |
| Ferguson 2006 | Border controls<br>Travel restriction | Local transmission phase |
| Zhang 2019 | Entry/exit screening | Early phase |

| Study ID | Short description of intervention | Phase of implementation |
|---|---|---|
| | · Entry screening (city-entry surveillance)<br>· Exit screening (city-exit surveillance)<br>· Follow up quarantine of positive cases<br>Travel reduction across cities (decrease in human mobility) | |
| Zhang 2020 | Lockdown of Wuhan and other cities in Hubei (referred in the study as "citywide quarantine of Wuhan and several nearby cities") | Local transmission phase |
| Zhang 2020a | Lockdown of Wuhan and other cities in Hubei ("Chinese government and people moved quickly to take measures to control the sources of infection and block the routes of transmission") | Early phase;<br>Local transmission phase |
| Zhao 2020 | Lockdown of Wuhan (referred to in the study as "a quarantine") | Early phase;<br>Local transmission phase;<br>Post-peak phase |
| Zhou 2020 | Lockdown of Wuhan (referred in the study as "strict control policies, including lockdown of Wuhan city") | Local transmission phase |
| **Studies assessing multiple diseases (COVID-19 and influenza)** | | |
| Kong 2020 | Lockdown of Wuhan ("Wuhan was locked down and China raised the national public health response to the first state of emergency") | Local transmission phase |
| **Studies assessing a hypothetical disease (COVID-19 relevant)** | | |
| Malmberg 2020 | · Inflow travel restrictions<br>· Quarantine of travelers (all those arriving) | Local transmission phase;<br>Post-peak phase |


For peer review only - http://bmjopen.bmj.com/site/about/guidelines.xhtml

**S7:** Outcome categories, types and specific outcomes assessed in studies

| Type | Outcomes included | Number of studies |
|---|---|---|
| **Category 1. Infectious disease outcomes** | | |
| Number or proportion of cases | Number of cases (predicted, reported, detected) | 58 |
| | Number of cases as rates per e.g. 1000, 100000 population | 3 |
| | Number of imported/exported cases | 4 |
| | Number of cases at epidemic peak | 1 |
| | Attack rate | 7 |
| Number or proportion of deaths | Number of deaths | 6 |
| | Case fatality rate | 1 |
| Healthcare resources | Number of cases requiring ICU treatment | 1 |
| | Time until ICU capacity is reached | 1 |
| Reproduction number | Effective reproduction number | 12 |
| | Basic reproduction number | 5 |
| Temporal development of epidemic | Time to epidemic arrival | 22 |
| | Time to epidemic peak | 9 |
| | Time to 1000 cases | 2 |
| | Doubling rate | 1 |
| Probability of epidemic | Probability of epidemic | 4 |
| | Probability of avoiding epidemic | 1 |
| | Probability of sustained transmission | 1 |
| | Probability of releasing infected individuals | 1 |
| | Probability of developing symptoms | 1 |
| Other infectious disease outcomes | Transmission risk index | 1 |
| | Transmission routes | 1 |
| | Number of infected cities | 1 |
| **Category 2. Screening outcomes** | | |
| Detection of high risk persons or cases | Number or proportion screened that are identified as high risk | 10 |
| | Number or proportion screened that are diagnosed | 12 |
| | Number or proportion quarantined that are diagnosed | 3 |
| | Positive predictive value of screening measures/tests | 2 |
| | Probability of missing cases | 4 |
| **Category 3. Economic outcomes** | | |
| Costs | Value of measures | 2 |
| | Cost of measures as percentage of Gross Domestic Product | 1 |
| | Cost of preventing cases | 1 |
| Industry impact | Air traffic passenger volumes | 1 |
| **Category 4. Social outcomes** | | |
| Acceptability of measures | Attitudes towards travel restrictions | 1 |

**S8:** Overview of types of studies included in the evidence map

### Inferential studies

**Compartmental models**
Common in infectious disease research, these include, for example the SIR model (comprising three compartments, S: susceptible, I: infectious, R: recovered). Several included studies applied such models, and extended the SIR model by adding other compartments, such as E: exposed, NS: non-susceptible, L: latent, I: isolated, D: dead

Examples from included studies: Kim et al. 2017; Mandal et al. 2020; Mbuvha et al. 2020

**Spatial models**
Several included studies applied models to explore how disease transmission moves spatially. Such models use assumptions and/or data about how humans move to predict disease transmission. Included studies examined, for example, mobility data from smartphone usage or global flight patterns.

Examples from included studies: Bajardi et al. 2011; Chinazzi et al. 2020; Clifford et al. 2020; Colizza 2020; Kraemer et al. 2020; Pullano et al. 2020

**Epidemiological time-series models**
Many included studies investigated the temporal nature of the disease data, and applied time-series approaches common to epidemiology. They applied a range of approaches, including simple time-series analysis, auto-regressive integrated moving average models, and change-point analysis to explore changes over time.

Examples from included studies: Chung et al. 2015; Jiang et al. 2020; Lin 2020_trends; Yuan 2020a

**Other epidemiological techniques**
Included studies also used more simple epidemiological designs and and statistical methods, such as correlation analysis, linear regression and statistical testing (e.g. chi-square test).

Examples from included studies: Fujita et al. 2011; Lau et al. 2020; Wang et al. 2020d

**Mixed approaches**
Several included studies used a range of approaches; combinations included compartmental models and spatial models, compartmental models and time-series models.

Examples from included studies: Epstein et al. 2007; Linka et al. 2020; Longxiang et al. 2020; Marcelino et al. 2013; Wang et al. 2020

### Descriptive studies

**Observational studies**

Several included studies described the impact of control measures through summary statistics; these comprised, for example, the number or proportion of screened individuals that were classified as high risk and the number or proportion of screened individuals that were diagnosed.

Examples from included studies: Arima et al. 2020; Hale et al. 2020; Kuo et al. 2009; Sakaguchi et al. 2012

**Graphical summary studies**
A small number of included studies used graphical summary techniques to describe the impact of control measures. These illustrated, for example, how the number of cases developed in time relative to the introduction of control measures.

Examples from included studies: Cheng et al. 2020; de Vlas et al. 2009; Pinkas et al. 2020

For peer review only


For peer review only - http://bmjopen.bmj.com/site/about/guidelines.xhtml

## Supplementary file

## S1: Preferred Reporting Items for Systematic reviews and Meta-Analyses extension for Scoping Reviews (PRISMA-ScR) Checklist

| SECTION | ITEM | PRISMA-ScR CHECKLIST ITEM | REPORTED ON PAGE # |
|---|---|---|---|
| **TITLE** | | | |
| Title | 1 | Identify the report as a scoping review. | |
| **ABSTRACT** | | | |
| Structured summary | 2 | Provide a structured summary that includes (as applicable): background, objectives, eligibility criteria, sources of evidence, charting methods, results, and conclusions that relate to the review questions and objectives. | |
| **INTRODUCTION** | | | |
| Rationale | 3 | Describe the rationale for the review in the context of what is already known. Explain why the review questions/objectives lend themselves to a scoping review approach. | |
| Objectives | 4 | Provide an explicit statement of the questions and objectives being addressed with reference to their key elements (e.g., population or participants, concepts, and context) or other relevant key elements used to conceptualize the review questions and/or objectives. | |
| **METHODS** | | | |
| Protocol and registration | 5 | Indicate whether a review protocol exists; state if and where it can be accessed (e.g., a Web address); and if available, provide registration information, including the registration number. | |
| Eligibility criteria | 6 | Specify characteristics of the sources of evidence used as eligibility criteria (e.g., years considered, language, and publication status), and provide a rationale. | |
| Information sources* | 7 | Describe all information sources in the search (e.g., databases with dates of coverage and contact with authors to identify additional sources), as well as the date the most recent search was executed. | |
| Search | 8 | Present the full electronic search strategy for at least 1 database, including any limits used, such that it could be repeated. | |
| Selection of sources of evidence† | 9 | State the process for selecting sources of evidence (i.e., screening and eligibility) included in the scoping review. | |
| Data charting process‡ | 10 | Describe the methods of charting data from the included sources of evidence (e.g., calibrated forms or forms that have been tested by the team before their use, and whether data charting was done independently or in duplicate) and any processes for obtaining and confirming data from investigators. | |
| Data items | 11 | List and define all variables for which data were sought and any assumptions and simplifications made. | |
| Critical appraisal of individual sources of evidence§ | 12 | If done, provide a rationale for conducting a critical appraisal of included sources of evidence; describe the methods used and how this information was used in any data synthesis (if appropriate). | |
| Synthesis of results | 13 | Describe the methods of handling and summarizing the data that were charted. | |

St. Michael's
Inspired Care.
Inspiring Science.

For peer review only - http://bmjopen.bmj.com/site/about/guidelines.xhtml

| SECTION | ITEM | PRISMA-ScR CHECKLIST ITEM | REPORTED ON PAGE # |
|---|---|---|---|
| **RESULTS** | | | |
| Selection of sources of evidence | 14 | Give numbers of sources of evidence screened, assessed for eligibility, and included in the review, with reasons for exclusions at each stage, ideally using a flow diagram. | |
| Characteristics of sources of evidence | 15 | For each source of evidence, present characteristics for which data were charted and provide the citations. | |
| Critical appraisal within sources of evidence | 16 | If done, present data on critical appraisal of included sources of evidence (see item 12). | |
| Results of individual sources of evidence | 17 | For each included source of evidence, present the relevant data that were charted that relate to the review questions and objectives. | |
| Synthesis of results | 18 | Summarize and/or present the charting results as they relate to the review questions and objectives. | |
| **DISCUSSION** | | | |
| Summary of evidence | 19 | Summarize the main results (including an overview of concepts, themes, and types of evidence available), link to the review questions and objectives, and consider the relevance to key groups. | |
| Limitations | 20 | Discuss the limitations of the scoping review process. | |
| Conclusions | 21 | Provide a general interpretation of the results with respect to the review questions and objectives, as well as potential implications and/or next steps. | |
| **FUNDING** | | | |
| Funding | 22 | Describe sources of funding for the included sources of evidence, as well as sources of funding for the scoping review. Describe the role of the funders of the scoping review. | |

JBI = Joanna Briggs Institute; PRISMA-ScR = Preferred Reporting Items for Systematic reviews and Meta-Analyses extension for Scoping Reviews.

* Where *sources of evidence* (see second footnote) are compiled from, such as bibliographic databases, social media platforms, and Web sites.

† A more inclusive/heterogeneous term used to account for the different types of evidence or data sources (e.g., quantitative and/or qualitative research, expert opinion, and policy documents) that may be eligible in a scoping review as opposed to only studies. This is not to be confused with *information sources* (see first footnote).

‡ The frameworks by Arksey and O'Malley (6) and Levac and colleagues (7) and the JBI guidance (4, 5) refer to the process of data extraction in a scoping review as data charting.

§ The process of systematically examining research evidence to assess its validity, results, and relevance before using it to inform a decision. This term is used for items 12 and 19 instead of "risk of bias" (which is more applicable to systematic reviews of interventions) to include and acknowledge the various sources of evidence that may be used in a scoping review (e.g., quantitative and/or qualitative research, expert opinion, and policy document).

*From:* Tricco AC, Lillie E, Zarin W, O'Brien KK, Colquhoun H, Levac D, et al. PRISMA Extension for Scoping Reviews (PRISMAScR): Checklist and Explanation. Ann Intern Med. 2018;169:467–473. doi: 10.7326/M18-0850.



For peer review only - http://bmjopen.bmj.com/site/about/guidelines.xhtml
